# Supplementary material for: Expediting hit-to-lead progression in drug discovery through reaction prediction and multi-dimensional optimization
Source: Nat Commun. 2025 Nov 26;16:11646. doi: 10.1038/s41467-025-66324-4 (PMC12749626; doi:10.1038/s41467-025-66324-4)
Supplement: Supplementary file 1 — Supplementary Information [file 41467_2025_66324_MOESM1_ESM.pdf]

Supplementary information

Expediting hit-to-lead progression in drug discovery through reaction prediction and multi-dimensional optimization

David F. Nippa[1,†], Kenneth Atz[1,†], Yannick Stenzhorn[1], Alex T. Müller[1], Andreas Tosstorff[1], Jörg Benz[1], Hayley Binch[1], Markus Bürkler[1], Achi Haider[1], Dominik Heer[1], Remo Hochstrasser[1], Christian Kramer[1], Michael Reutlinger[1], Petra Schneider[2,3], Thierry Shema[4], Andreas Topp[1], Alexander Walter[1], Matthias B. Wittwer[1], Jens Wolfard[1], Bernd Kuhn[1], Mario van der Stelt[4], Rainer E. Martin[1,*], Uwe Grether[1,*] & Gisbert Schneider[2,3,*]

[1]Roche Pharma Research and Early Development (pRED), Roche Innovation Center Basel, F. Hoffmann-La Roche Ltd., Grenzacherstrasse 124, 4070 Basel, Switzerland.
[2]ETH Zurich, Department of Chemistry and Applied Biosciences, Vladimir-Prelog-Weg 4, 8093 Zurich, Switzerland.
[3]ETH Zurich, Department of Biosystems Science and Engineering, CURE, Klingelbergstrasse 48, 4056 Basel, Switzerland.
[4]Leiden University, Leiden Institute of Chemistry, Department of Molecular Physiology, Einsteinweg 55, 2333 CC Leiden, The Netherlands.
† These authors contributed equally to this work.
E-mail rainer_e.martin@roche.comm uwe.grether@roche.com, gisbert@ethz.ch

Contents

SI1 Machine learning model performance **2**

SI2 Biological data of synthesized compounds **2**
SI2.1 Potency data . . . 2
SI2.2 Physicochemical properties . . . 4
SI2.3 Off-target selectivity data (CEREP) . . . 8
SI2.4 Activity-based protein profiling (ABPP) data . . . 10
SI2.5 Protein-ligand co-crystallization . . . 14
SI2.5.1 Data collection and refinement statistics . . . 14
SI2.5.2 Electrondensity . . . 15

SI3 High-throughput experimentation (HTE) protocol for the generation of Minisci-type alkylation reaction data **16**

SI4 Fragment, carboxylic acid and lead molecule structures **17**
SI4.1 Structures of fragments . . . 17
SI4.2 Structures of carboxylic acids . . . 24

SI5 Scale-up reactions **43**
SI5.1 Reagent and purification information . . . 43
SI5.2 Analytical information . . . 43
SI5.3 Experimental procedures and analytical data . . . 44
SI5.3.1 Synthesis of head groups . . . 44
SI5.3.2 Synthesis of adaptable building block . . . 55
SI5.3.3 Synthesis of alkylated building blocks . . . 56
SI5.3.4 Synthesis of MAGL inhibitor molecules . . . 61

SI6 NMR spectra **75**

SI1 Machine learning model performance

In the context of machine learning model performance, the terms 0D, 1D, and 2D refer to different data splitting strategies used to evaluate the model's ability to generalize to new data. The 0D split involves predicting outcomes for known combinations of reactants, resulting in the highest performance metrics (e.g., MAE = 6.7%, $r = 0.831$). The 1D splits test the model's extrapolation to novel acids (1DA) or N-arenes (1DN), showing increased errors and lower correlations. The 2D split, which involves predicting outcomes for novel combinations of both acids and N-arenes, demonstrates the model's capacity to generalize to entirely new chemical spaces, albeit with slightly reduced performance metrics compared to the 0D split. The results are summarized in Table S1.

Table S1: Machine learning model performance.

**Dataset split**	**Mean absolute error (MAE) / %**	**Pearson correlation coefficient (r)**	**Accuracy**	**Recall**	**Precision**
0D	6.7 (± 0.1)	0.831 (± 0.009)	0.857 (± 0.004)	0.703 (± 0.006)	0.909 (± 0.007)
1DN	11.4 (± 0.6)	0.551 (± 0.1)	0.720 (± 0.05)	0.527 (± 0.12)	0.757 (± 0.06)
1DA	12.5 (± 0.8)	0.512 (± 0.08)	0.692 (± 0.07)	0.510 (± 0.09)	0.787 (± 0.03)
2D	12.6 (± 2.5)	0.527 (± 0.1)	0.702 (± 0.02)	0.515 (± 0.03)	0.854 (± 0.06)

SI2 Biological data of synthesized compounds

SI2.1 Potency data

Table S2: Biological data of synthesized compounds.

MAGL Inhibitor	IC_{50} human/ nM	IC_{50} mouse/ nM	IC_{50} rat/ nM	IC_{50} cyno/ nM	IC_{50} nanoBRET clone 43/ µM	IC_{50} nanoBRET clone 63/ µM	IC_{50} nanoBRET clone 21/ µM
**17**	445	557	79	531	$>$ 100	-	-
**18**	9	32	-	-	-	-	-
**19**	8	117	-	-	-	-	-
**20**	50	$>$ 10000	26	61	5.1	33.9	4.5
**21**	10	27	2	12	3.9	0.8	0.2
**22**	66	189	18	68	19.2	2.3	0.4
**23**	4 (±1.3)	109 (±1.1)	6 (±1.2)	5 (±1.1)	5.9	13.8	0.6
**24**	7 (±1.2)	71 (±1.2)	2 (±1.1)	7 (±1.1)	2.7	3.7	0.2
**25**	434 (±1.1)	$>$ 10000	206 (±1.0)	581 (±1.6)	$>$ 100	$>$ 100	39.7
**26**	348 (±1.3)	6426	155 (±1.3)	547 (±1.2)	$>$ 100	$>$ 100	28.6
**27**	2 (±1.2)	86 (±1.5)	8 (±1.2)	2 (±1.5)	14.5	55.4	1.0
**28**	19 (±1.3)	369 (±2.1)	13 (±1.2)	28 (±1.3)	10.9	29.9	0.7
**29**	0.1 (±1.6)	3 (±1.7)	0.7 (±1.2)	0.1 (±2.4)	0.3	0.5	0.2
**30**	60 (±1.3)	253 (±1.4)	99 (±1.2)	84 (±1.2)	11.8	16.9	10.0
**31**	0.6 (±1.3)	0.8 (±1.3)	1 (±1.1)	0.8 (±1.2)	0.1	0.06	0.05

SI2.2 Physicochemical properties

Parallel Artificial Membrane Permeability (PAMPA) Parallel Artificial Membrane Permeability was conducted as previously reported [1]. Briefly, permeability across a phospholipid-coated filter plate was assessed using the PAMPA. For each test, 15 µL of a 10 mM drug solution in DMSO (or DMSO blank as control) was added to 985 µL of donor buffer (50 mM MOPSO, pH 6.0, containing 0.5% [w/v] glycocholic acid) in a 2 mL 96-deepwell plate. The resulting mixture was filtered through a 0.45 µm filter plate into another 96-deepwell plate. UV absorption analysis was performed using a SPECTRAmax Plus 384 UV reader After an 18-hour incubation at room temperature, 150 µL of both donor and acceptor solutions were transferred to UV plates, and UV absorption was recorded between 246 and 500 nm. The effective permeability (P_{PAMPA}) was calculated following the equation:

$$P_{\mathrm{PAMPA}} = -\frac{V_A \cdot V_D}{V_A + V_D} \cdot \frac{1}{A \cdot T} \cdot \ln\left(1 - \frac{C_t}{C_0} \cdot \frac{V_A + V_D}{V_D}\right) \tag{1}$$

where VD and VA represent the volumes of the donor and acceptor compartments, A is the membrane area adjusted for filter porosity, t is the incubation time, and C_t and C_0 are the concentrations in the acceptor and donor compartments, respectively. Each compound permeability was evaluated in two independent experiments with two samples. Average values and standard deviations were calculated from the four individual logarithmic P_{PAMPA} values (or three, if one measurement failed).

P-glycoprotein (P-gp) Assay P-glycoprotein (P-gp) efflux ratios were obtained in porcine kidney epithelial LLC-PK1 cells stably transfected with ABCB1 (MDR1, human P-gp). As previously reported [2], in vitro transport studies were performed in triplicate in the apical-to-basolateral directions on a Tecan robotic pipetting device, whereby compound concentrations were analyzed by LC-MS/MS. Experiments were conducted with and without P-gp inhibitor (zosuquidar). Edoxaban - a known P-gp substrate - was used as a positive control. Apparent permeability (Papp) of the test compounds was determined as follows:

$$P_{\mathrm{app}} = \frac{\Delta Q}{\Delta t} \cdot \frac{1}{C_0} \cdot S \tag{2}$$

where $\frac{\Delta Q}{\Delta t}$ is the amount of test compound transported per time period, C_0 is the initial concentration, and S is the inset surface area.

$$\frac{P_{\mathrm{appA \to B}} \text{ (with inhibitor)}}{P_{\mathrm{appA \to B}} \text{ (without inhibitor)}} \tag{3}$$

where $P_{\mathrm{appA \to B}}$ equals the apparent permeability in the apical-to-basolateral direction.

Hepatocyte clearance Hepatocyte clearance was assessed as previously reported [3]. Briefly, cryopreserved hepatocytes of human origin were thawed and reconstituted (hepatocyte viability remained at least 80% throughout the study). Primary pooled cryopreserved hepatocytes were reconstituted in pre-warmed William's E medium, supplemented with 10% FCS, 0.05 mg/mL streptomycin, 50 U/mL penicillin, 0.4 mM L-glutamine, 0.01 mg/mL gentamicin, 0.048 mg/mL hydrocortisone, and 0.004 mg/mL insulin, to achieve a final suspension density of 1×10^6 cells/mL. Incubations were conducted using a fully automated Liquid Handling System (Tecan) equipped with a CO_2 incubator and orbital shaker. Test compounds (1 µM) were added to the wells (1×10^5 cells/well), and the 96-well hepatocyte suspension plates were incubated at 37°C in 5% CO_2. At designated time points (up to 2 hours), samples were quenched by adding MeCN as well as an internal standard to each incubation well. All samples were centrifuged and the supernatant analyzed via LC-MS/MS.
The in vitro intrinsic clearance (CL_{int}) was calculated using the slope of the linear regression for log (%- initial drug concentration) vs. time as follows:

Table S3: ADMET property data of synthesized compounds.

MAGL Inhibitor	CYP2C9 Inhibition / %	CYP2D6 Inhibition / %	CYP3A4 Inhibition / %	Solubility in 0.05M PO_4^-/ µg/mL	Solubility in FESSIF/ µg/mL
**17**	2	-24	16	4.2	-
**18**	62	45	> 90	< 0.05	-
**19**	49	23	44	< 1.1	-
**20**	24	16	49	< 0.05	-
**21**	74	41	4	< 0.05	-
**22**	23	62	-59	< 0.05	-
**23**	45	38	> 90	< 0.05	1.9
**24**	45	26	8	< 0.05	2.5
**25**	-5	34	70	84	-
**26**	-23	18	12	> 590	-
**27**	73	79	> 90	< 0.6	-
**28**	-	-	-	-	-
**29**	70	34	84	< 0.05	25
**30**	-19	3	-40	< 0.05	-
**31**	43	22	> 90	-	-

$$\begin{aligned}&\text{In vitro } CL_{\text{int}} \left(\frac{\text{L}}{\text{min} \cdot 10^6 \text{ hepatocytes}}\right) \\ &= -\text{slope} \cdot \frac{V_{\text{incubation}}}{10^6 \text{ hepatocytes per incubation}}\end{aligned} \tag{4}$$

Midazolam (CYP3A4 substrate) and dextromethorphan (CYP2D6) were used as controls for human hepatocyte clearance, whereas midazolam (CYP3A4 substrate) and bupropion (CYP2B6) were used as controls for the mouse hepatocyte assay.

Microsomal clearance Microsomal clearance was assessed as previously described [3]. Briefly, a high throughput microsomal stability assay was performed at 37°C on a TECAN automated liquid handling system. In an initial step, cryopreserved mouse or human microsomes were thawed and re-suspended in phosphate buffer. The target compounds were added to the microsomes to give a final incubation concentration of 1 µM (test compound) and 0.5 mg/mL (microsomes). After a 10-minute pre-incubation period at 4°C, the enzymatic reaction was started by adding the cofactor NADPH. Aliquots of the incubations were removed at 1, 3, 6, 9, 15, 25, 35, and 45 minutes, and quenched with 1:3 (v/v) acetonitrile containing internal standard. Samples were then cooled and centrifuged before analysis of the supernatant by LC-MS/MS. Midazolam for CYP3A4, Bupropion for CYP2B6, Dextromethorphan for CYP2D6, and Diclofenac for CYP2C9 were used as controls.

Cytochrome P450 (CYP) inhibition assay Cytochrome P450 (CYP) inhibition assays were conducted as previously reported [4]. Briefly, the target compound was added to a suspension of mouse or human liver microsomes (HLM) in 100 mM sodium phosphate buffer (pH 7.4), followed by the addition of the respective CYP probe substrate at around the reported K_m. The microsomes were pre-incubated for 3 minutes at 37°C. The reaction was initiated by adding 10 µL of 10 mM NADPH (final concentration of 1 mM). Samples were incubated for the appropriate time to allow the probe substrate reaction. Reversible inhibitors included: alpha-Naphthoflavone (CYP1A2), tamoxifen (CYP2B6), quercetin (CYP2C8), sulphaphenazole (CYP2C9), N-3-benzylnirvanol (CYP2C19), quinidine (CYP2D6), and ketoconazole (CYP3A4/5).

Table S4: ADMET property data of synthesized compounds.

MAGL Inhibitor	Microsomal clearance human / µL/min/mg	Microsomal clearance mouse / µL/min/mg	Hepatocyte clearance / µL/min/10^6 cells	PAMPA permeability/ 10^{-6} cm/s	P-gp / nm/s
**17**	10	10	7.3	4.0	117
**18**	853	-	-	-	-
**19**	17	47	4.6	-	-
**20**	-	-	84.2	-	-
**21**	53	114	47.7	3.7	100
**22**	142	-	104.5	2.7	81
**23**	415	176	178.8	4.0	89
**24**	69	61	42.2	8.5	93
**25**	10	10	1.0	0.02	-
**26**	10	10	1.0	0.01	-
**27**	1000	1000	101.0	0.9	71
**28**	-	-	200.0	5.8	-
**29**	425	536	180.6	5.9	132
**30**	563	-	187.2	4.4	-
**31**	431	-	162.6	12.1	-

Lipohpylicity-corrected ligand efficiency and permeability optimization

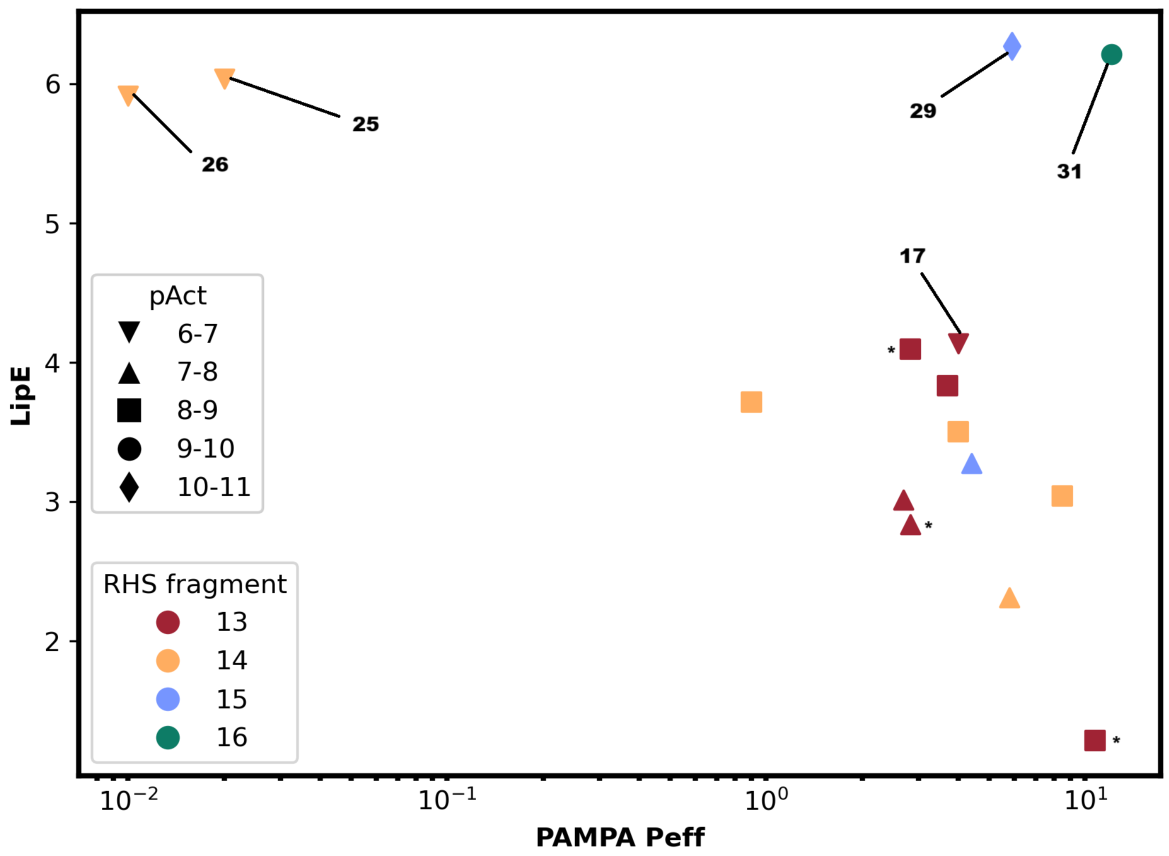

Figure S1: Lead optimization plot showing PAMPA permeability (Peff $cm/s * 10^{-6}$) vs. lipophilicity corrected ligand efficiency (LipE). * predicted values.

SI2.3 Off-target selectivity data (CEREP)

Representative off-target assessment. The data was generated at Eurofins Cerep (France). The data shows the mean percentage of inhibition for binding assays and the mean percentage of inhibition for enzyme and cell-based assays at a test concentration of 10 µM (n=2). **ABPP sample preparation**: Mouse brain was homogenized with glass beads (3/4x 1 min, bullet blender, speed 8) using cold lysis buffer (20 mM Hepes pH 7.2, 1 mM $MgCl_2$, 5 U/mL Benzonase). Membrane and cytosol was separated by centrifugation. Membrane fraction was resuspended in Hepes/DTT buffer. Protein concentration was determined with Bradford Assay and samples were diluted to a final concentration of 2.0 mg/mL. Samples were snap frozen in liquid nitrogen and stored at -80 °C until further use. **Gel-based ABPP**: Samples of cytosol and membrane fraction were thawed on ice. Samples were divided over tubes and incubated with either vehicle (2.5% DMSO), JZL-184 (10 µM, final concentration), or one of the three compounds (10 µM, final concentration) for 30 min at RT. Samples were then incubated with the probe cocktail (MB064 100 nM final and FP-Bodipy 100 nM final) for 10 minutes at RT. The reaction was quenched with 4x Laemlli buffer for 30 minutes at RT. Samples were resolved by SDS-PAGE (10% AA gel, 15 slots, 0.75 mm, 75 min, 180 V) along with a protein marker. In-gel fluorescence was measured with the Cy3-(MB064), Cy2- (FP-Bodipy) and Cy5 (Marker, 10s) channel. Exposure time in Cy3 was 120s and Cy2 (80s). Gels were stained with Coomassie for 10 min after scanning and destained overnight in DEMI water for protein loading control.

Table S5: Secondary pharmacology profiling results obtained from Eurofins CEREP. Values indicate the percent activity or inhibition compared to the assay reference at 10 uM. Values above 50% are marked in bold. H: human, R: rat, B: bovine, REC.: receptor, ENZ.: enzyme.

**CEREP Assay**	**Species**	**17**	**23**	**27**	**29**
5HT1A	H	1	11	28	19
5HT2A	H	7	2	12	10
**5HT2B**	H	28	3	10	**55**
5HT3	H	0	-6	-3	-2
ABL1	H	2	-8	-3	-5
ACETYLCHOLINESTERASE	H	3	-1	-4	0
**ADENOSINE A1 REC.**	H	**70**	24	13	9
ADENOSINE A3 REC.	H	13	21	7	28
**α1A-ADRENOCEPTOR**	H	13	4	5	**50**
α2A-ADRENOCEPTOR	H	7	3	0	18
ANDROGEN REC.	H	6	-10	-6	7
ANGIOTENSIN CONVERT. ENZ.	H	16	-17	-12	-13
ANGIOTENSIN II REC. 1	H	-3	11	-1	15
β1-ADRENOCEPTOR	H	4	2	4	7
β2-ADRENOCEPTOR	H	1	4	-2	7
**CA2+ CHANNEL (L-TYPE)**	R	40	**73**	**90**	32
CANNABINOID REC. CB1	H	2	2	18	9
CDK2	H	3	8	0	-6
CHOLECYSTOKININ 1 REC.	H	-12	9	4	8
CYCLO OXYGENASE 2	H	20	4	6	23
DOPAMINE D1 REC.	H	3	-1	-2	7
DOPAMINE D2 REC. (short)	H	-10	-15	13	4
ESTROGEN REC. α	H	0	5	-20	-3
**GABA-A**	R	-12	16	**69**	**61**
GABA-A (BENZODIAZAPINE SITE)	R	2	10	9	-1
GLUCOCORTICOID REC.	H	-13	-2	5	-6
GLYCINE REC.	R	-7	7	3	-2
GSK-3A	H	8	1	30	16
GSK-3B	H	-21	-20	-8	-19
HISTAMINE H1 REC.	H	13	11	48	18
HISTAMINE H2 REC.	H	1	-1	-4	11
HISTAMINE H3 REC.	H	14	4	15	12
HIV-1 PROTEASE	HIV	11	31	34	31
κ OPIOID REC.	H	15	18	48	32
MONOAMINE OXIDASE-A	H	7	2	-2	0
μ OPIOID REC.	H	48	21	16	30
**MUSCARINIC REC. M1**	H	9	10	**52**	8
**MUSCARINIC REC. M2**	H	27	40	**76**	33
MUSCULAR NICOTINIC REC.	H	31	0	2	24
NEURONAL NICOTINIC REC.	H	23	-3	-8	6
NOREPINEPHRINE TRANSP.	H	15	15	28	22
PCP NMDA REC.	R	7	7	8	-1
PHOSPHODIESTERASE 3B	H	2	-8	1	6
**PHOSPHODIESTERASE 4D2**	H	21	20	-7	**65**
PPARγ	H	-4	7	9	16
PROSTAGLANDIN F REC.	H	-2	-3	0	21
SEROTONIN TRANSPORTER	H	-5	10	1	-11
XANTHINE OXIDASE	B	2	6	6	3
ZAP70	H	-2	-27	-15	5

SI2.4 Activity-based protein profiling (ABPP) data

Mouse brain proteome – Cytosol and Membrane fraction

Control compounds: JZL-184

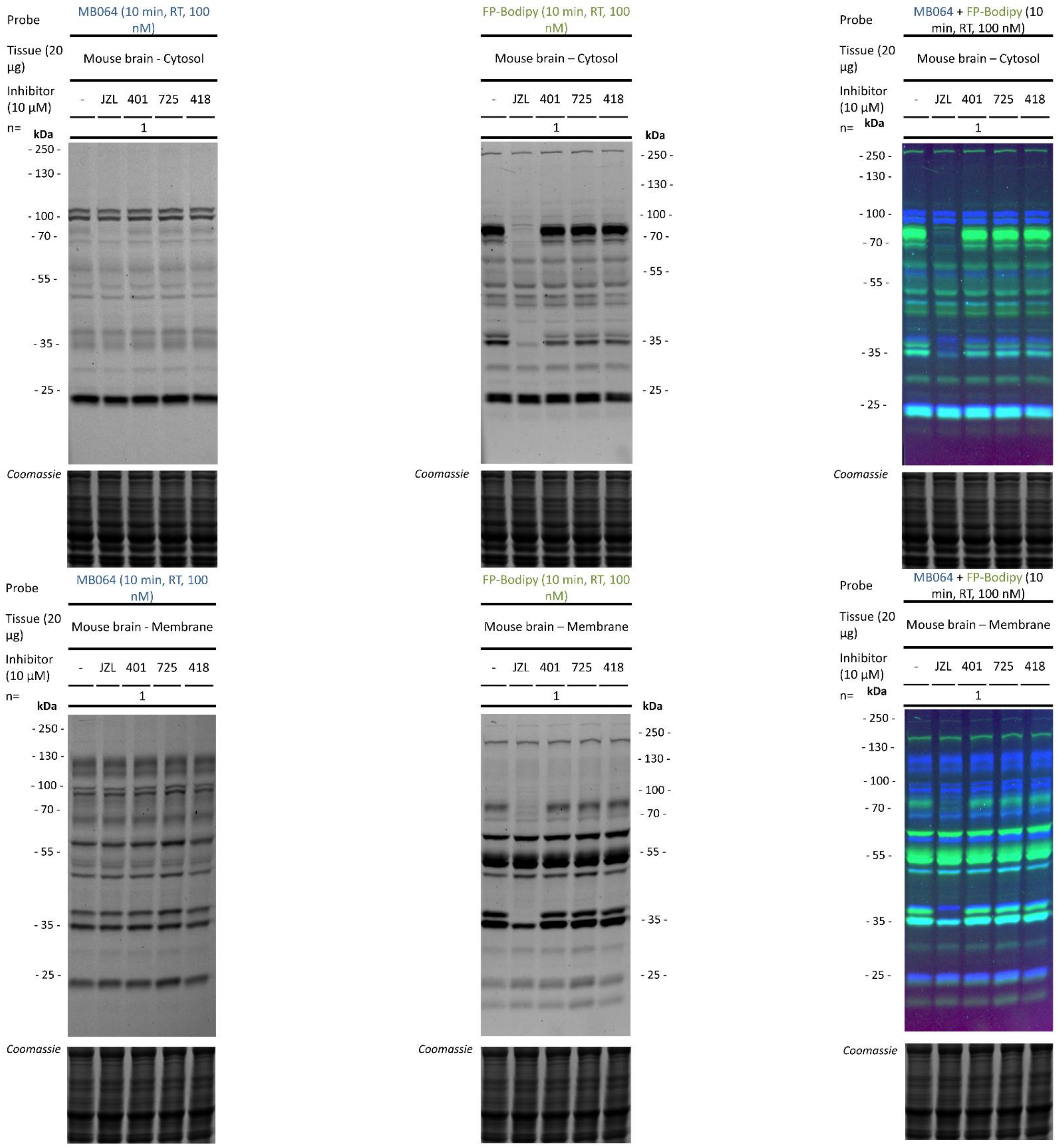

Sample #	Full name
JZL	JZL-184
401	RO7248401-000-002
725	RO7809725-000-001
418	RO7810418-000-001

Channel	Probe	Exposure time
Cy3	MB064	120s
Cy2	FP-Bodipy	80s
Cy5	Marker	10s

Mouse brain proteome – Cytosol and Membrane fraction

Control compounds: JZL-184

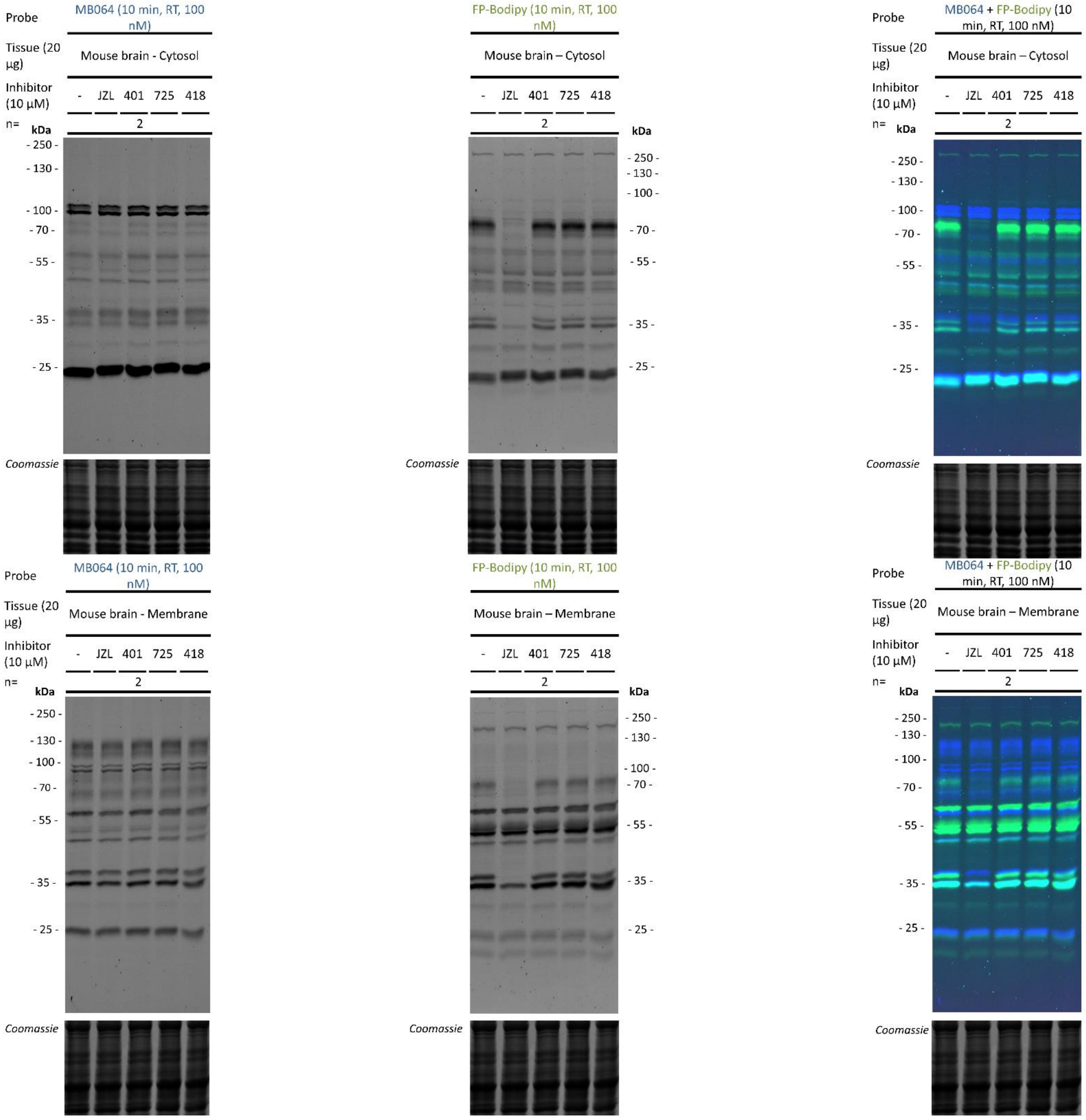

Sample #	Full name
JZL	JZL-184
401	RO7248401-000-002
725	RO7809725-000-001
418	RO7810418-000-001

Channel	Probe	Exposure time
Cy3	MB064	120s
Cy2	FP-Bodipy	80s
Cy5	Marker	10s

Mouse brain proteome – Percentage of inhibition (PCT) FP-Bodipy

Percentage of inhibition (PCT) in cytosol N=2

	JZL	401	725	418
PCT OF MAGL ± SEM	97,4 ± 0,2	44,3 ± 8,7	34,7 ± 10,5	42,1 ± 12,5
PCT OF MAGL/ABHD6 ± SEM	86,2 ± 2,0	34,9 ± 7,5	25,4 ± 8,2	31,0 ± 9,6

Percentage of inhibition (PCT) in membrane N =2

	JZL	401	725	418
PCT OF MAGL ± SEM	98,4 ± 0,4	11,1 ± 6,0	7,6 ± 4,0	10,3 ± 1,1
PCT OF MAGL/ABHD6 ± SEM	67,6 ± 4,0	5,9 ± 2,4	1,4 ± 3,7	10,0 ± 3,6

Mouse brain proteome – % FP-Bodipy labeling

% FP-Bodipy labeling in cytosol N=2

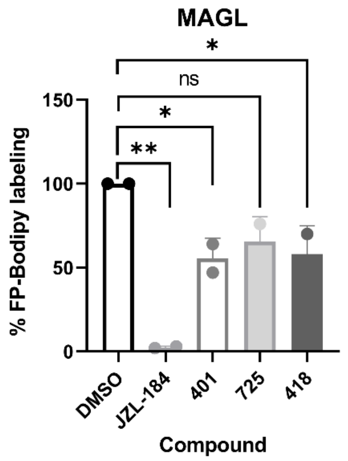

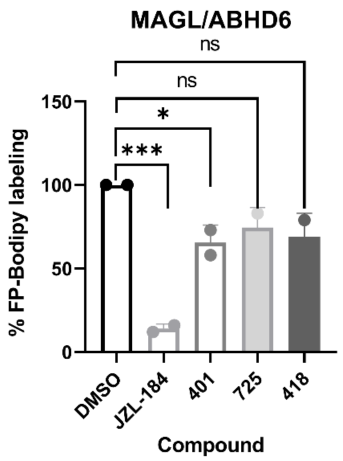

% FP-Bodipy labeling in Membrane N=2

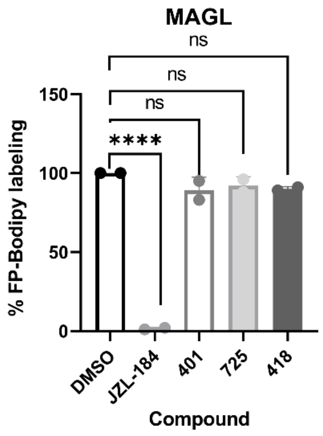

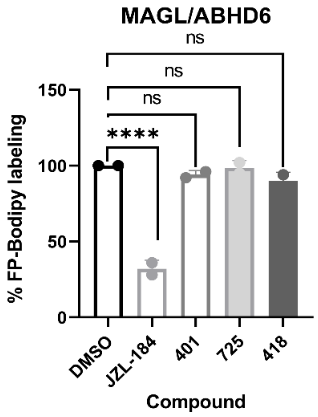

SI2.5 Protein-ligand co-crystallization

SI2.5.1 Data collection and refinement statistics

Table S6: Data collection and refinement statistics.

	Compound 17	Compound 23	Compound 27	Compound 29
**Data collection**				
Space group	C2221	C2221	C2221	C2221
Cell dimensions (Å)	89.86, 126.92, 62.75	89.05, 127.38, 62.61	89.18, 127.12, 62.53	89.26, 127.39, 62.55
α, β, γ (°)	90, 90, 90	90, 90, 90	90, 90, 90	90, 90, 90
Resolution (Å)	1.45 (1.55–1.45)	1.49 (1.59–1.49)	1.48 (1.58–1.48)	1.49 (1.59–1.49)
Total reflections	421 669	586 967	598 657	589 261
Unique reflections	63 798	58 359	59 049	58 455
R_{sym}	0.06 (0.98)	0.10 (0.98)	0.09 (0.97)	0.11 (0.94)
$I/\sigma I$	8.40 (0.63)	7.77 (0.69)	9.09 (0.75)	7.64 (0.74)
CC(1/2)	0.999 (0.676)	0.997 (0.623)	0.998 (0.535)	0.998 (0.604)
Completeness (%)	99.9 (100.0)	99.9 (99.9)	99.2 (98.5)	99.9 (99.9)
Redundancy	6.61 (6.85)	10.05 (10.28)	10.06 (9.95)	10.07 (9.87)
**Refinement**				
Resolution (Å)	44.62–1.45	32.69–1.49	38.27–1.48	38.35–1.49
No. reflections	63 499	58 079	58 812	58 282
$R_{\mathrm{work}}/R_{\mathrm{free}}$	17.86/21.23	19.62/22.80	18.89/20.51	17.61/21.32
**No. atoms**				
Protein	2 295	2 294	2 295	2 306
Water	222	34	210	38
Ligand	222	40	207	37
**B-factors**				
Protein	37.81	31.59	30.83	30.01
Water	48.06	35.19	41.36	37.30
Ligand	42.13	31.32	40.84	24.99
**R.m.s. deviations**				
Bond lengths (Å)	0.005	0.006	0.006	0.005
Bond angles (°)	0.753	0.871	0.808	0.820
PDB codes	9I3Y	9I56	9I5J	9I9C

SI2.5.2 Electrondensity

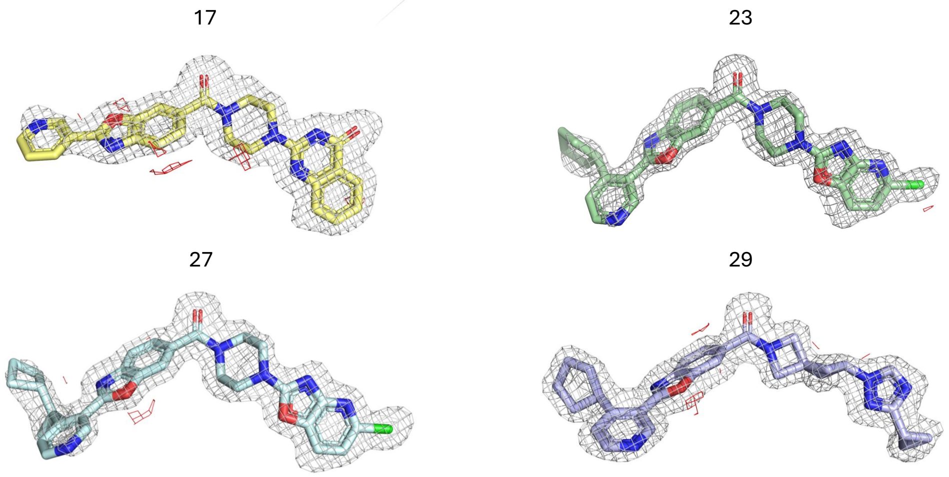

Figure S2: Omit map showing the electron density around the MAGL inhibitors **17**, **23**, **27** and **29**. Simulated-annealing omit Fo-Fc maps were calculated using Phenix. Electron density contoured at $+3\sigma$ is shown as grey mesh, contoured at -3σ shown as red mesh.

SI3 High-throughput experimentation (HTE) protocol for the generation of Minisci-type alkylation reaction data

All generated screening data used the plate design depicted in the paper (Figure 2F) and the procedure below. All solid reaction components were dissolved in an appropriate solvent to give stock solutions. Then according to the plate design, the stock solutions were transferred into 1 mL glass vials from Analytical Sales (Flanders, US) on a 24- or 96-well plate from Analytical Sales (Flanders, US) using a liquid handler EVO100 from Tecan (Männedorf, CH). The plate was heated on stirring plates from from IKA (Staufen, DE) or Radleys (Saffron Walden, UK) and VP 721F-1 Parylene Encapsulated Stainless Steel Stir Discs from V&P Scientific Inc. (San Diego, US) were used to stir the reaction mixture. Only one internal process control (IPC) was taken after 18 hours. Reaction solvents were removed using a Genevac centrifugal evaporator. Next, the crude was re-suspended using a Freedom EVO 100 liquid handler from Tecan (Männedorf, CH) with MeCN/H_2O (4:1) to a defined concentration (1 mmol/L). After stirring on stirring plates from IKA (Staufen, DE) or Radleys (Saffron Walden, UK), the samples were transferred onto a 96-deep-well plate (1 mL) from Eppendorf (Hamburg, DE). The plates were analyzed on a Waters (Milford, US) UPLC-MS system equipped with a Waters Acquity sample manager with a flow-through needle, a Waters Acquity sample organizer and a Waters QDa single quadrupole mass spectrometer. The separation was achieved on a ZORBAX RRHT Eclipse Plus C18, 95 Å, 2.1 x 30 mm, 1.8 µm column (P/N 959731-902, LOT: USUXY02479) from Agilent (Santa Clara, USA) at 50 °C. A 2-minute gradient was used and the injection volume accounted for 2 µL. 2 min gradient: A: 0.1% HCOOH in H_2O; B: 0.07% HCOOH in MeCN at flow 1 mL/min. Gradient: 0 min, 3% B; 0.2 min, 3% B; 1.5 min, 97% B; 0.3 min, 97% B; 0.1 min 3% B. The raw data were processed with MassLynx V4.2 and the obtained .rpt file underwent parsing with a customized script, before being subjected to the automated reaction data analysis pipeline. [5]

SI4 Fragment, carboxylic acid and lead molecule structures

SI4.1 Structures of fragments

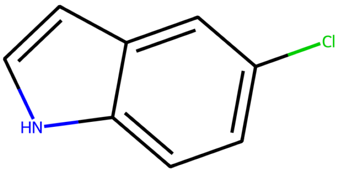

F1

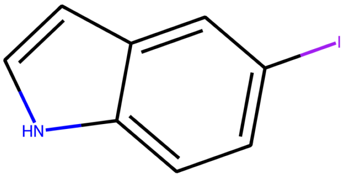

F2

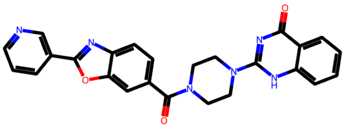

F3

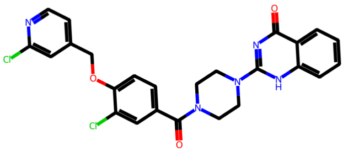

F4

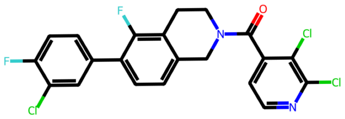

F5

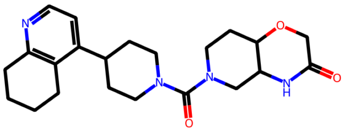

F6

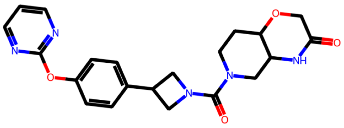

F7

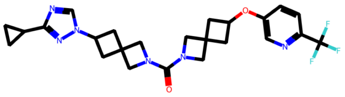

F8

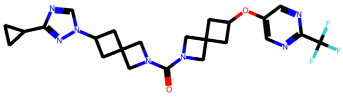

F9

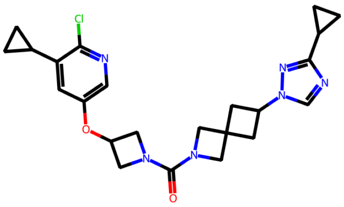

F10

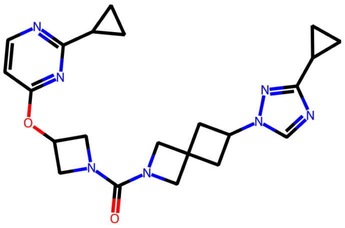

F11

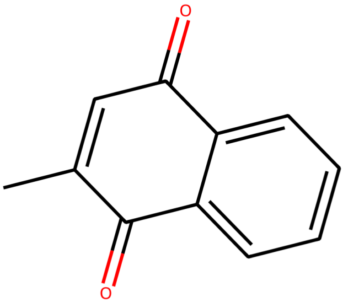

F12

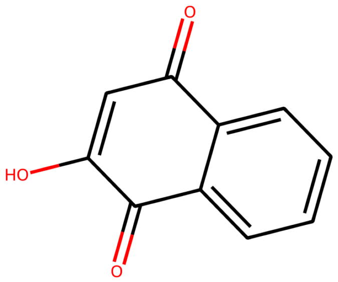

F13

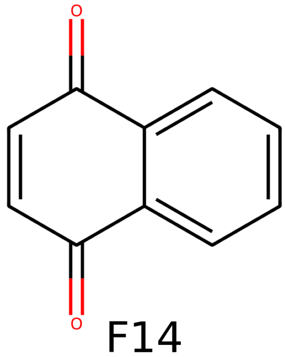

F14

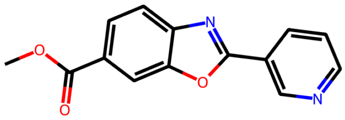

F15

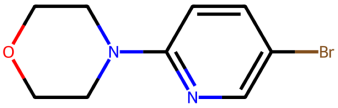

F16

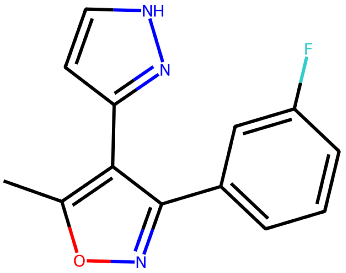

F17

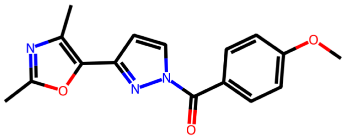

F18

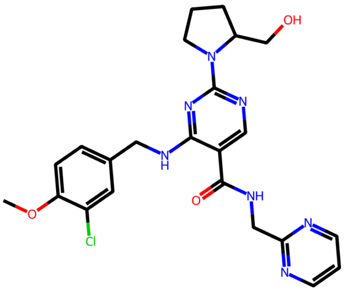

F19

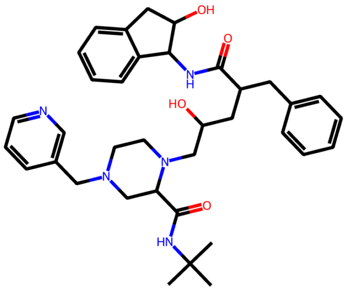

F20

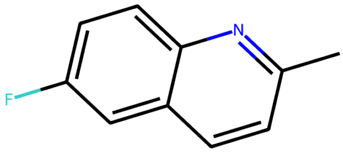

F21

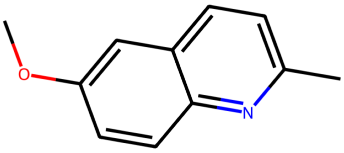

F22

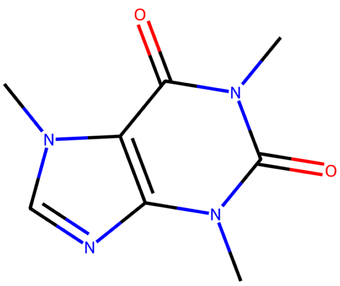

F23

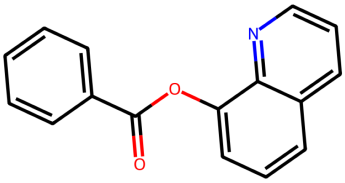

F24

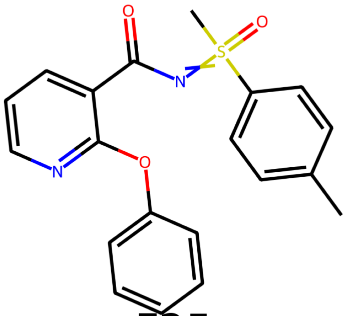

F25

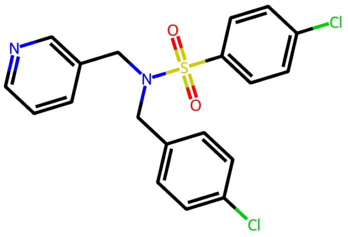

F26

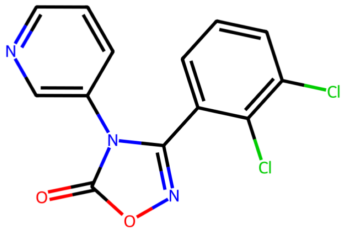

F27

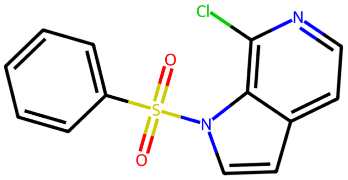

F28

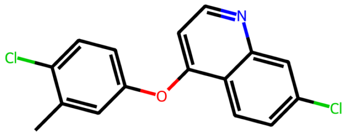

F29

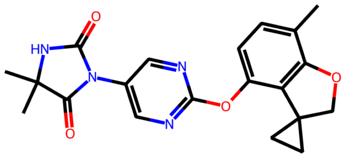

F30

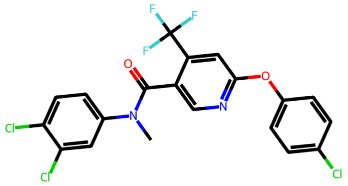

F31

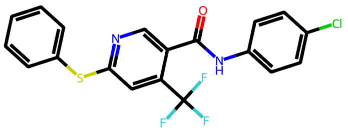

F32

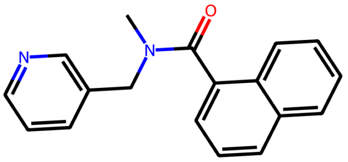

F33

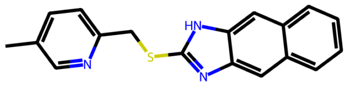

F34

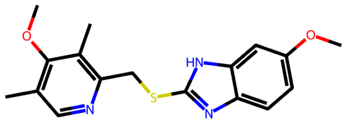

F35

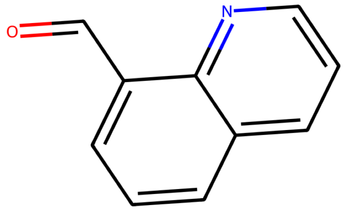

F36

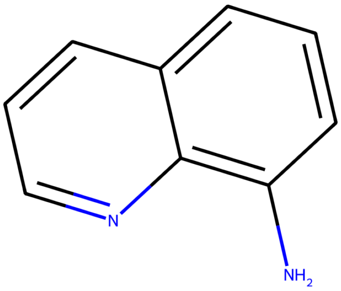

F37

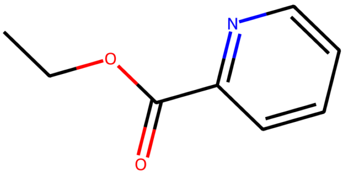

F38

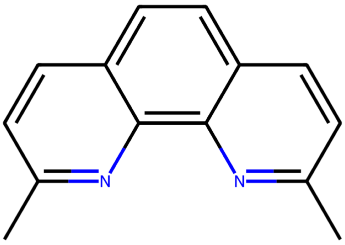

F39

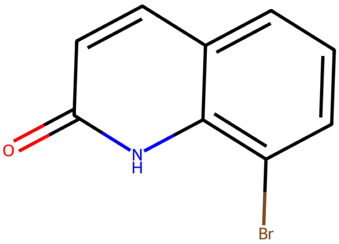

F40

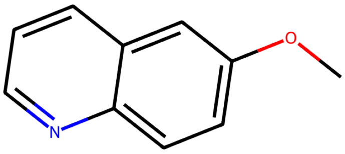

F41

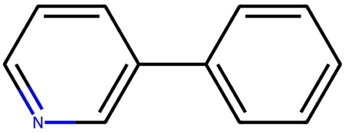

F42

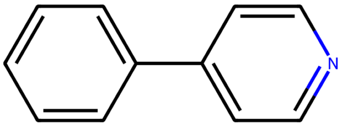

F43

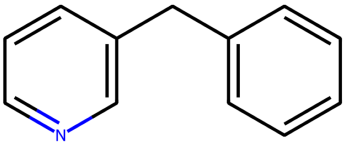

F44

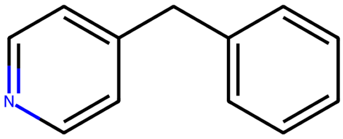

F45

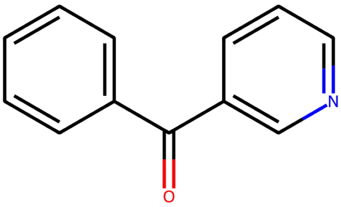

F46

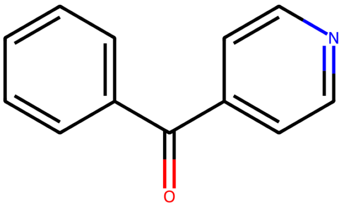

F47

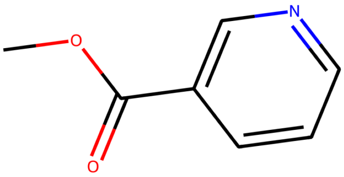

F48

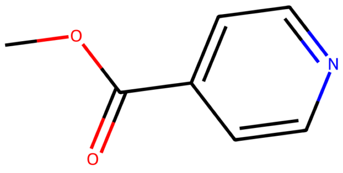

F49

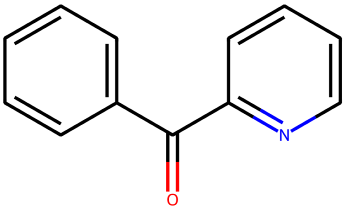

F50

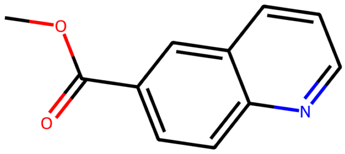

F51

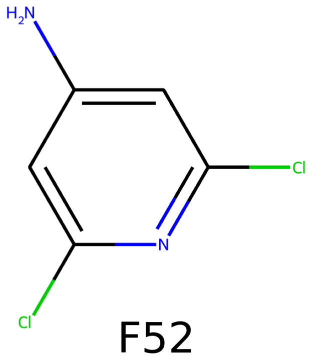

F52

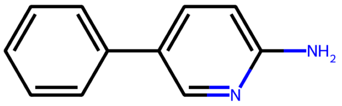

F53

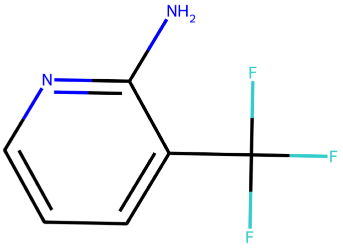

F54

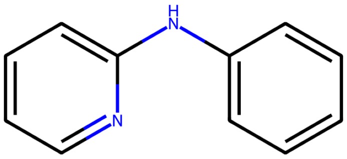

F55

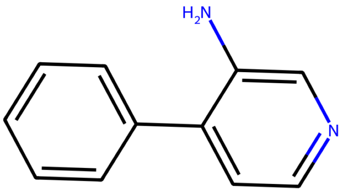

F56

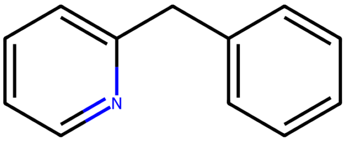

F57

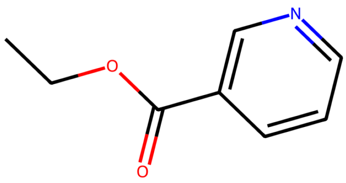

F58

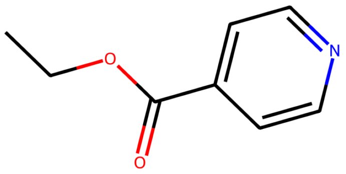

F59

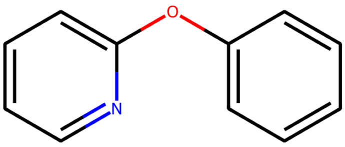

F60

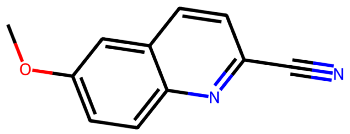

F61

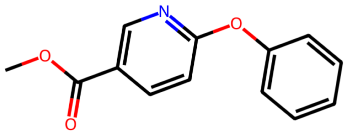

F62

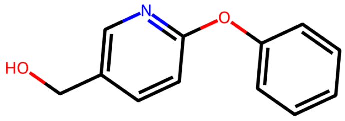

F63

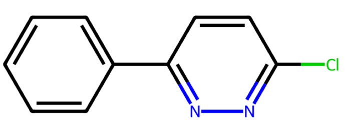

F64

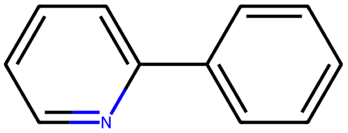

F65

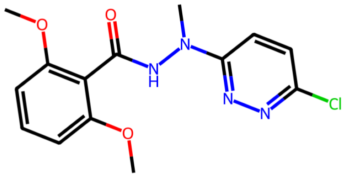

F66

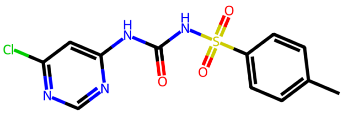

F67

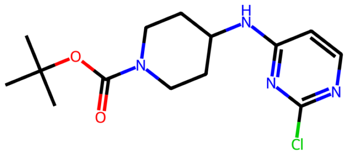

F68

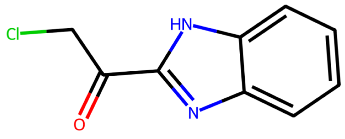

F69

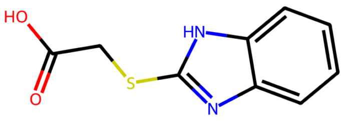

F70

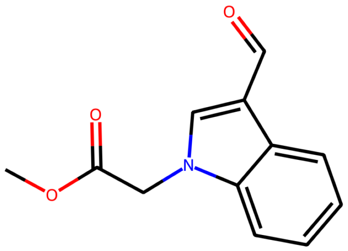

F71

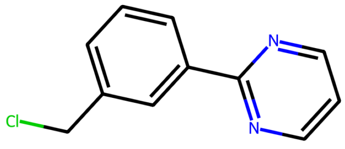

F72

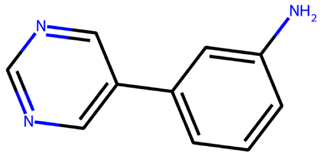

F73

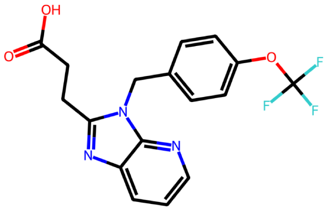

F74

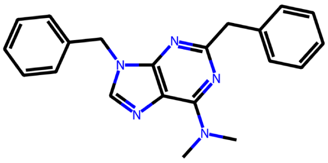

F75

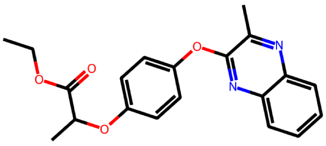

F76

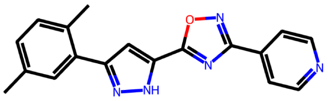

F77

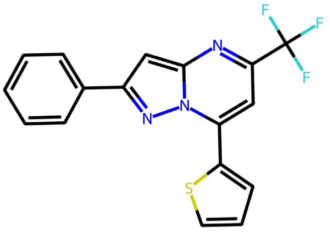

F78

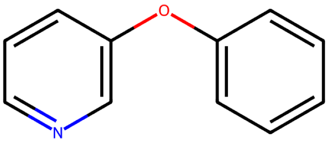

F79

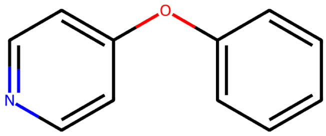

F80

SI4.2 Structures of carboxylic acids

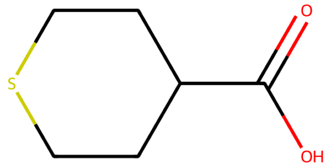

A1

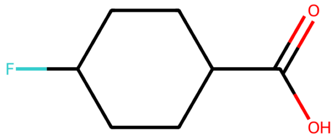

A2

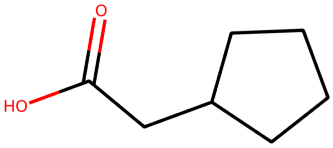

A3

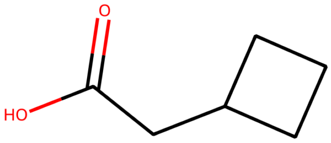

A4

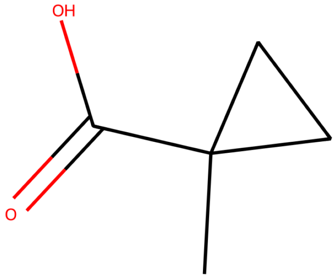

A5

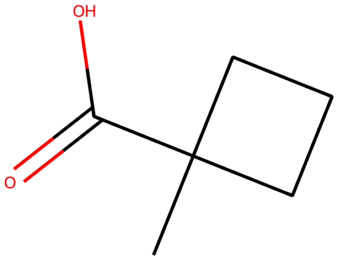

A6

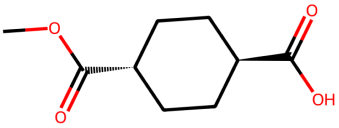

A7

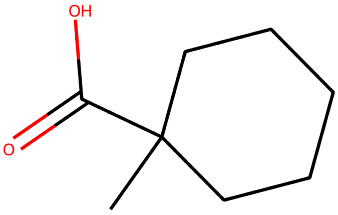

A8

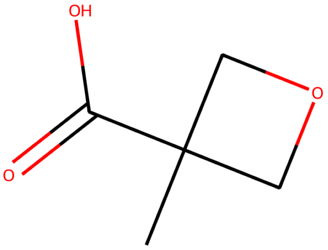

A9

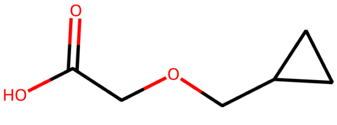

A10

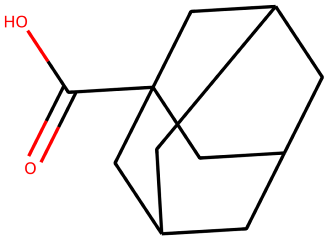

A11

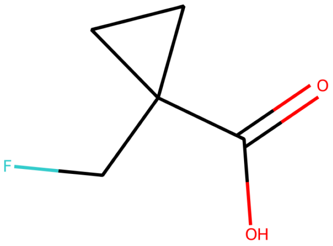

A12

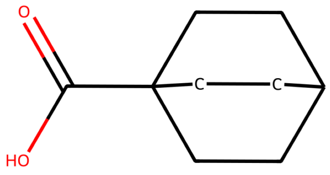

A13

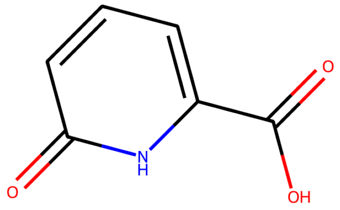

A14

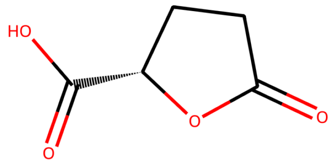

A15

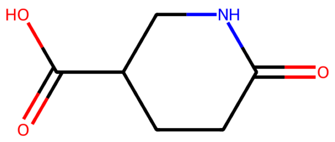

A16

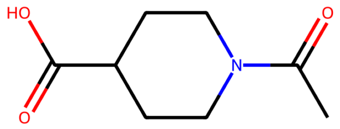

A17

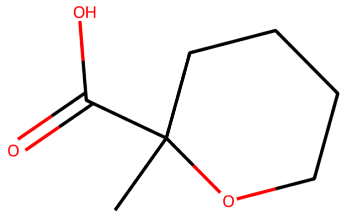

A18

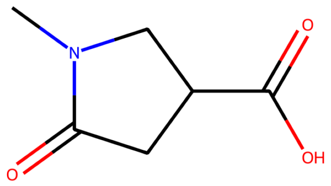

A19

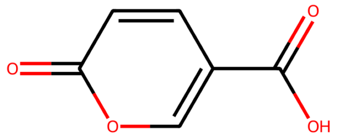

A20

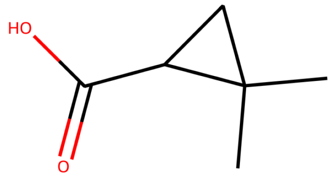

A21

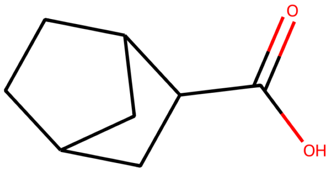

A22

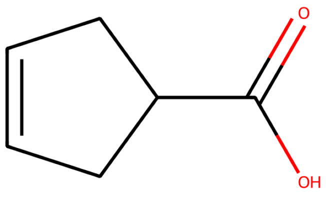

A23

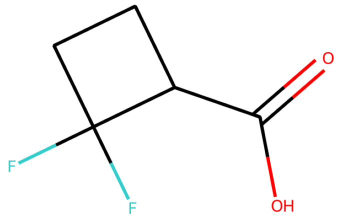

A24

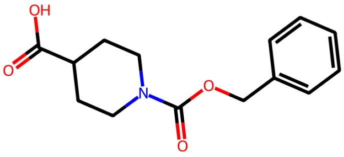

A25

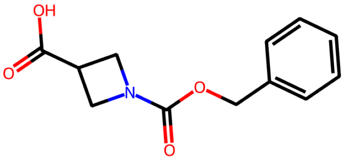

A26

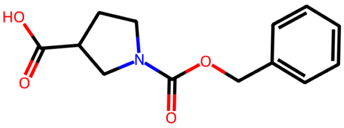

A27

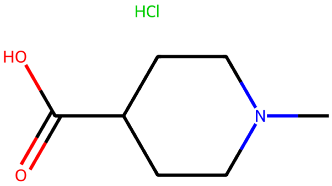

A28

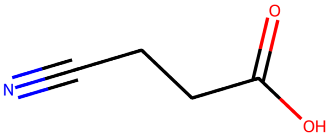

A29

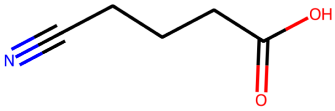

A30

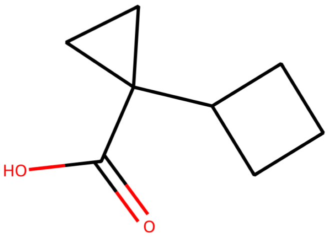

A31

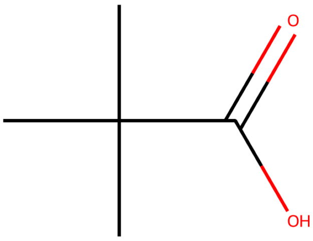

A32

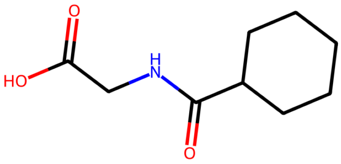

A33

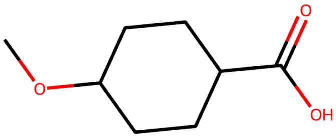

A34

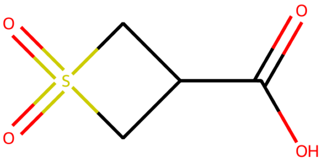

A35

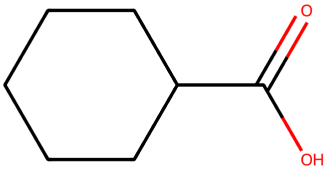

A36

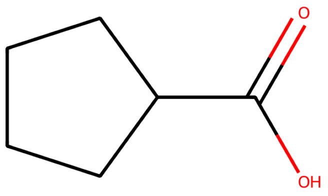

A37

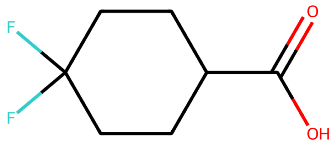

A38

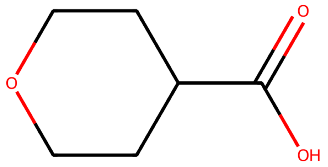

A39

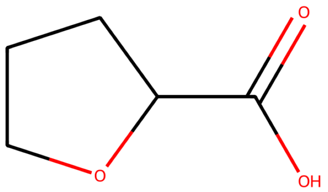

A40

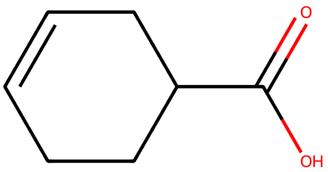

A41

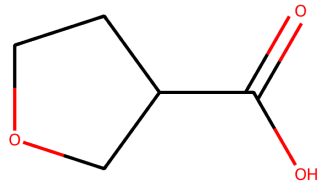

A42

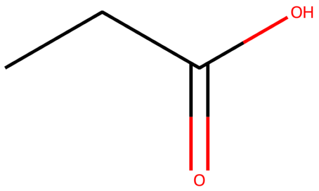

A43

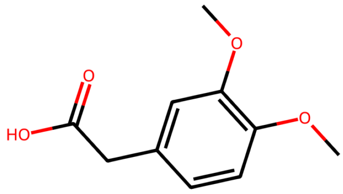

A44

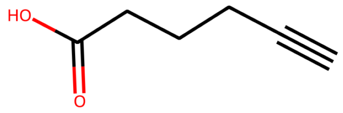

A45

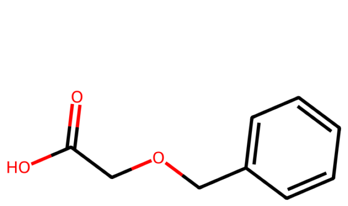

A46

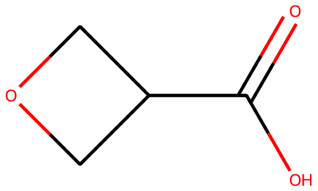

A47

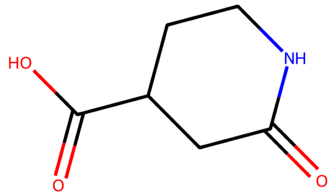

A48

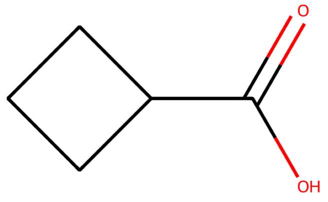

A49

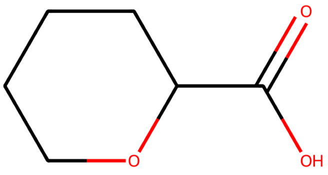

A50

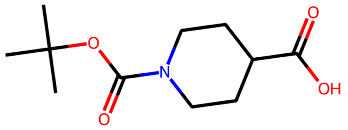

A51

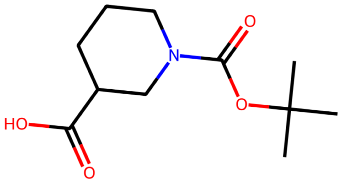

A52

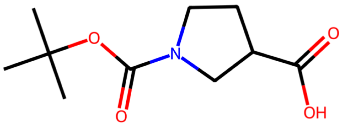

A53

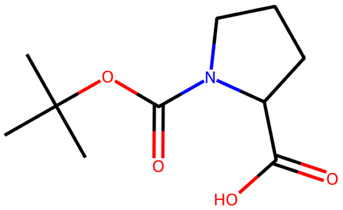

A54

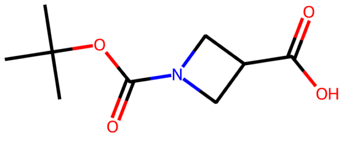

A55

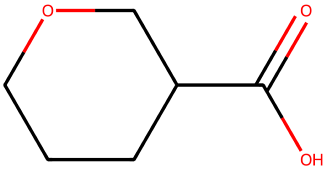

A56

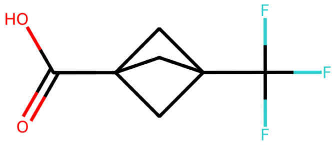

A57

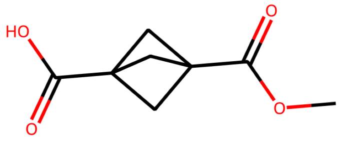

A58

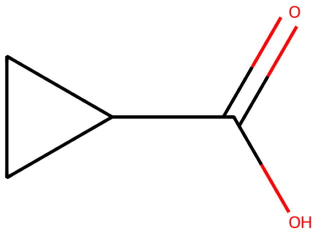

A59

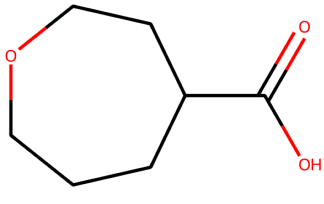

A60

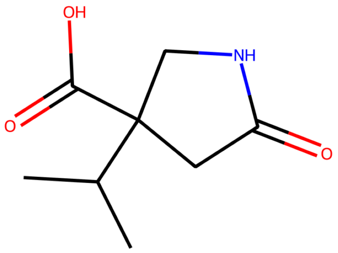

A61

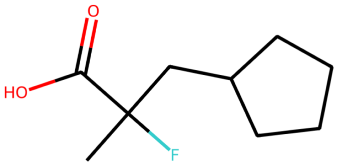

A62

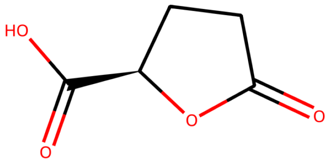

A63

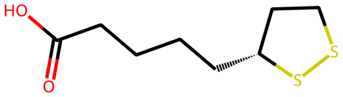

A64

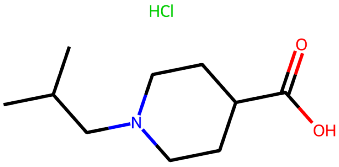

A65

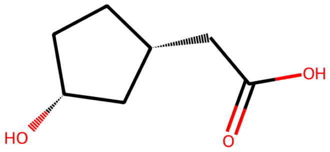

A66

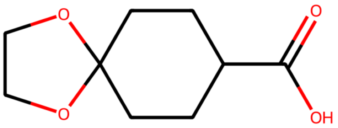

A67

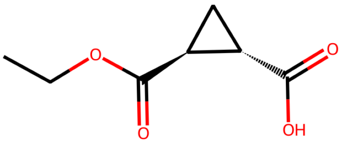

A68

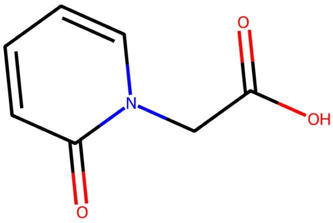

A69

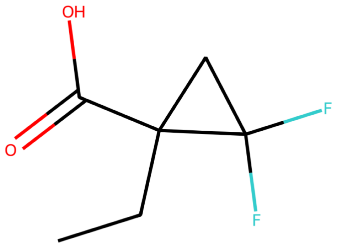

A70

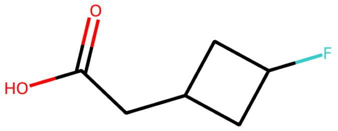

A71

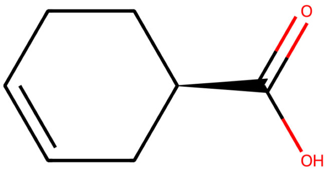

A72

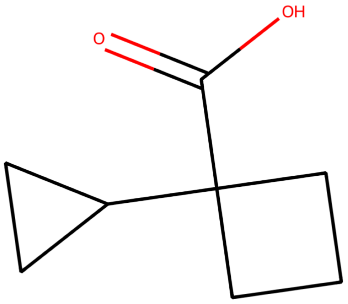

A73

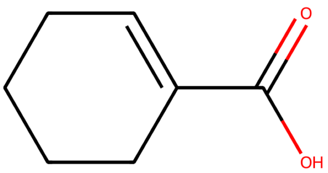

A74

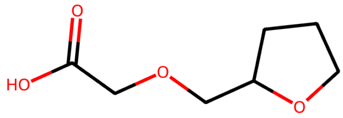

A75

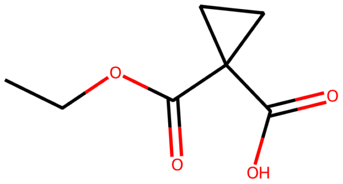

A76

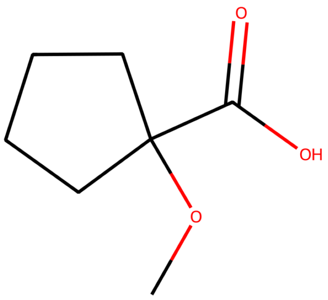

A77

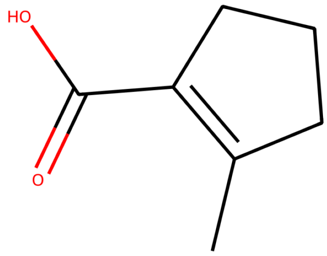

A78

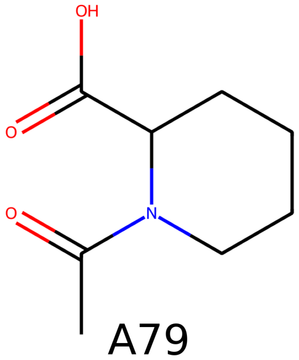

A79

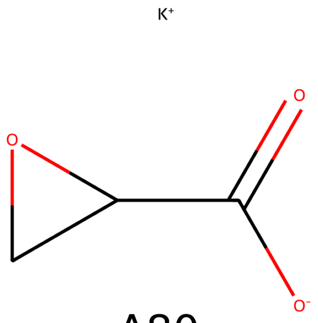

A80

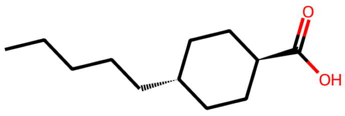

A81

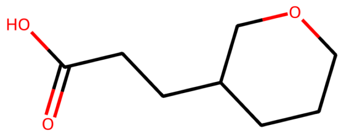

A82

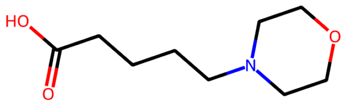

A83

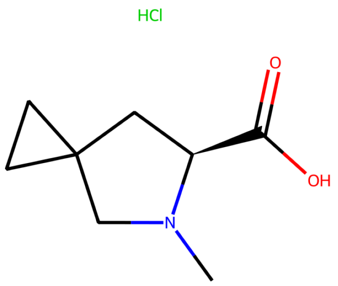

A84

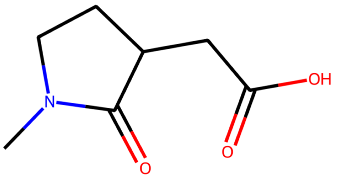

A85

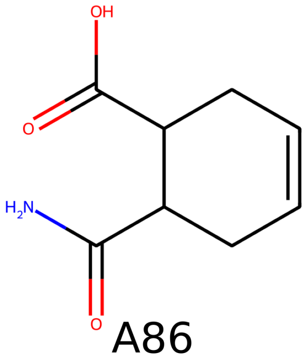

A86

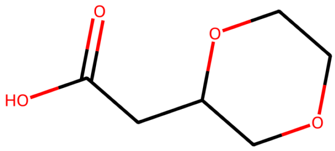

A87

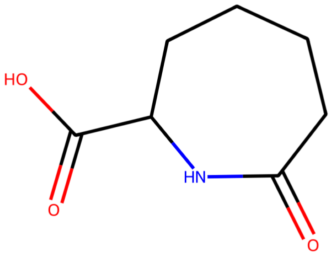

A88

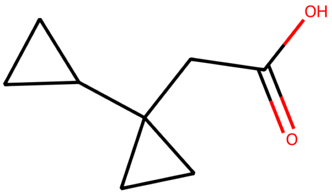

A89

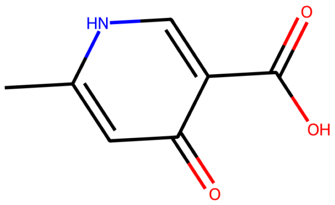

A90

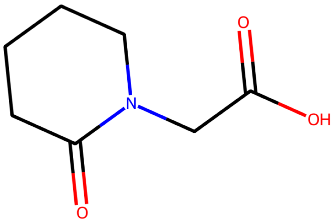

A91

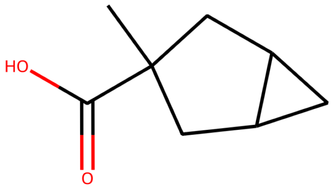

A92

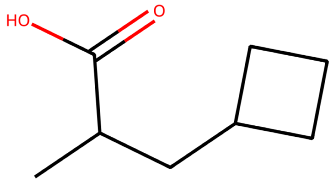

A93

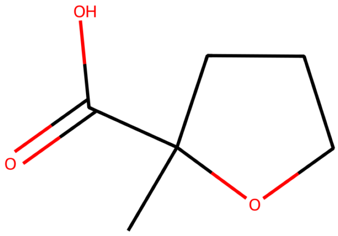

A94

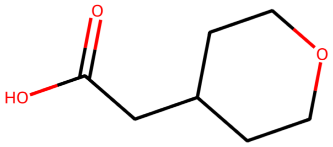

A95

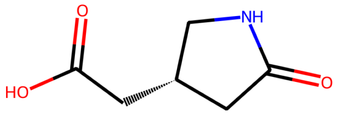

A96

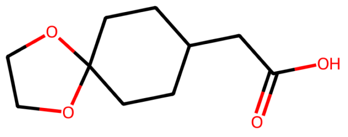

A97

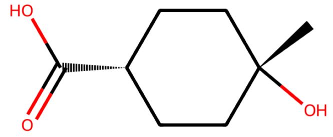

A98

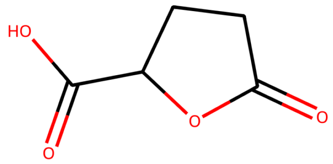

A99

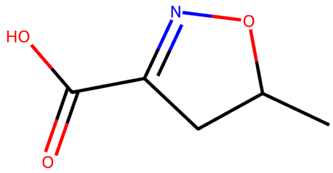

A100

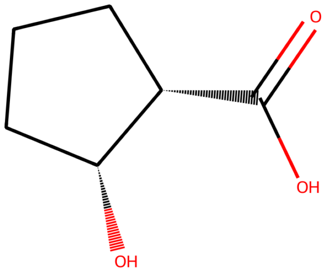

A101

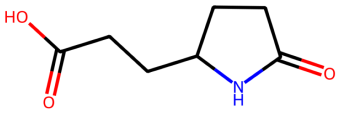

A102

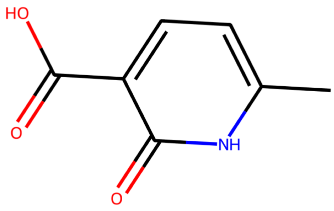

A103

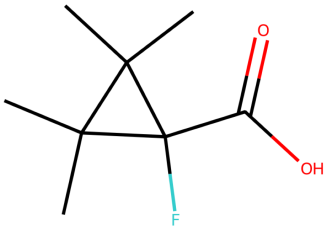

A104

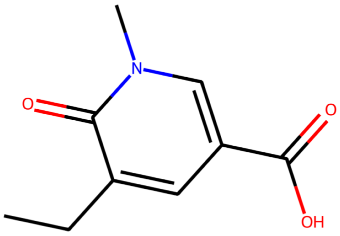

A105

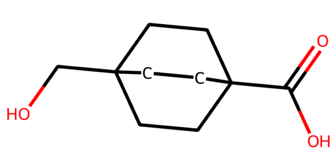

A106

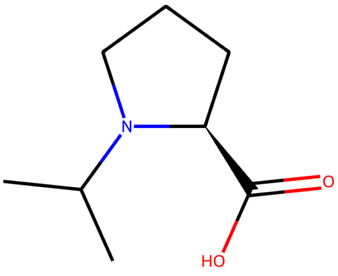

A107

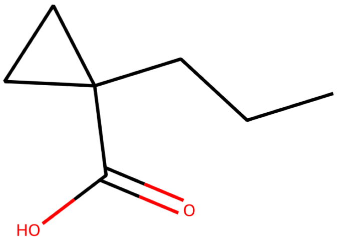

A108

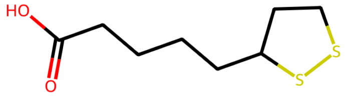

A109

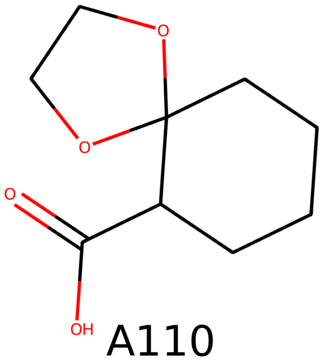

A110

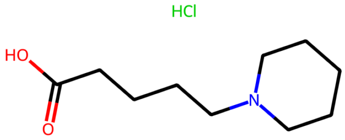

A111

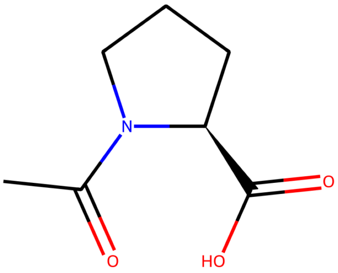

A112

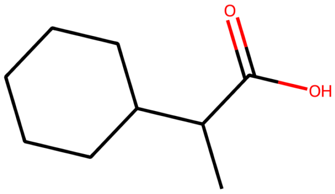

A113

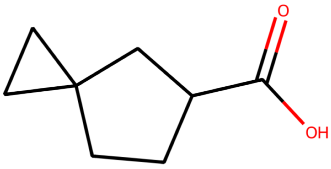

A114

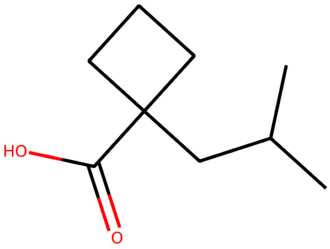

A115

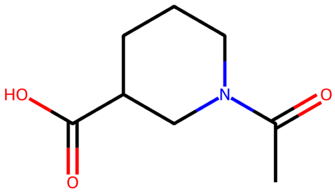

A116

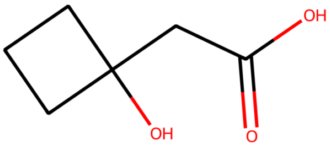

A117

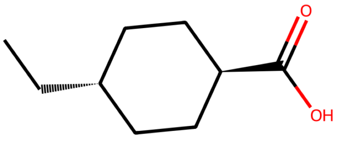

A118

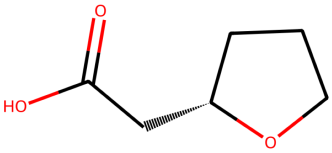

A119

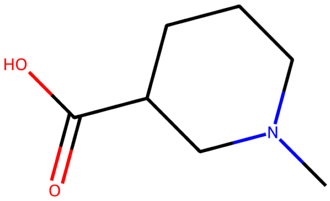

A120

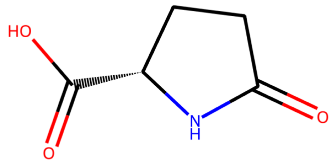

A121

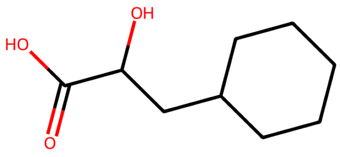

A122

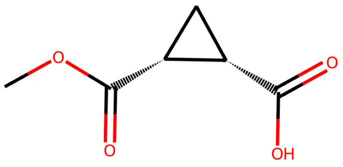

A123

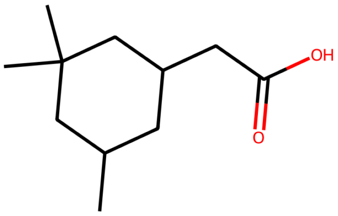

A124

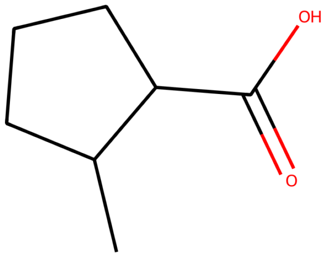

A125

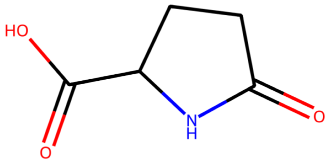

A126

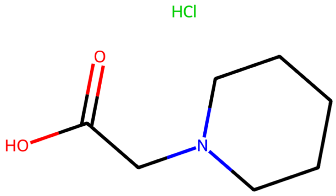

A127

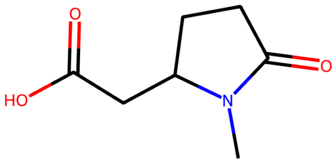

A128

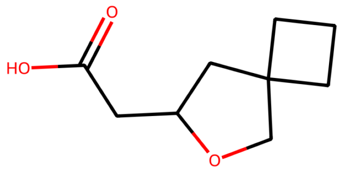

A129

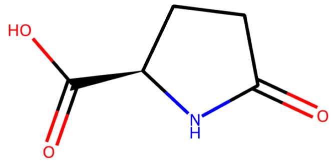

A130

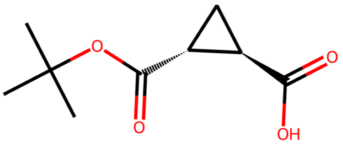

A131

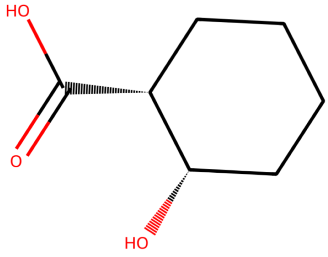

A132

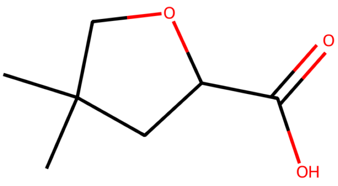

A133

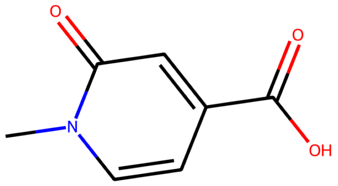

A134

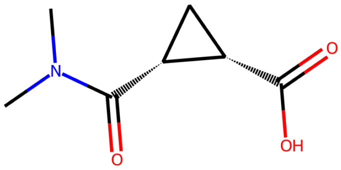

A135

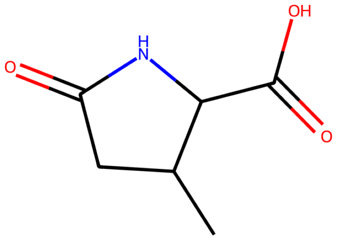

A136

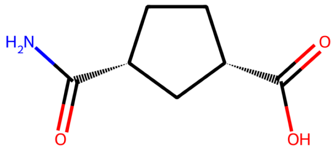

A137

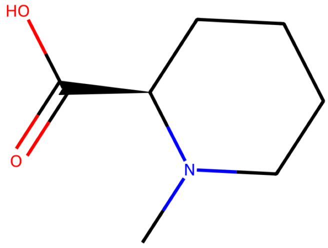

A138

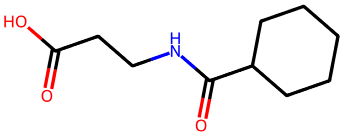

A139

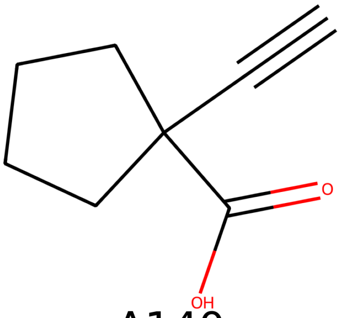

A140

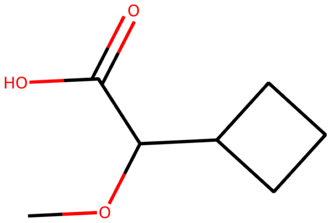

A141

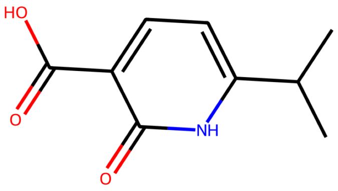

A142

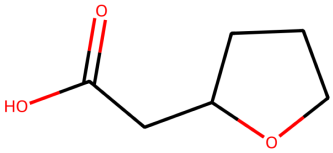

A143

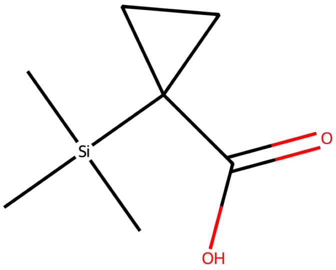

A144

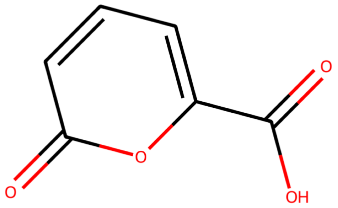

A145

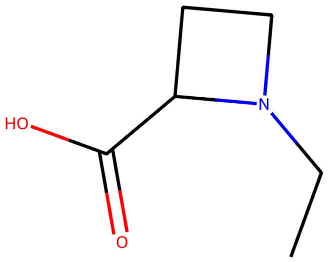

A146

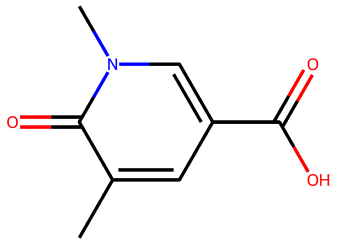

A147

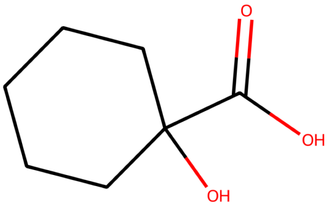

A148

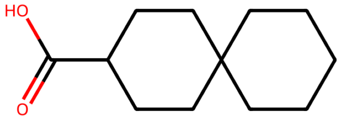

A149

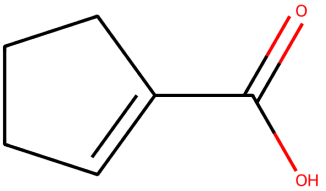

A150

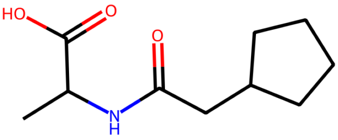

A151

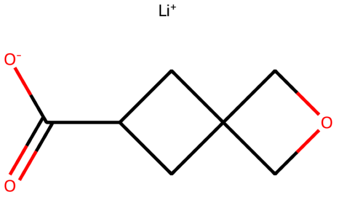

A152

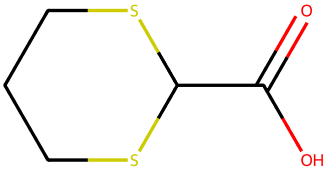

A153

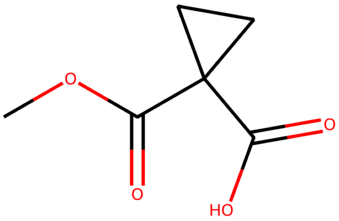

A154

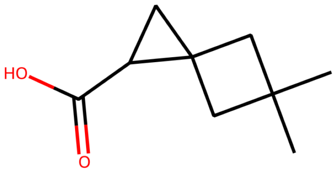

A155

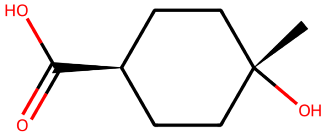

A156

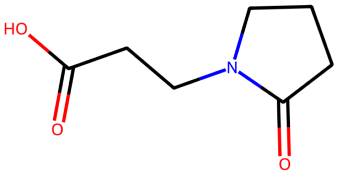

A157

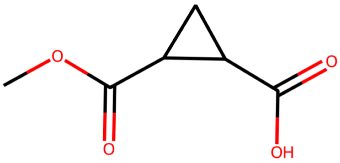

A158

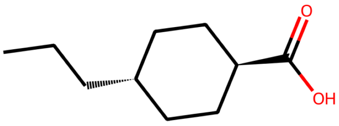

A159

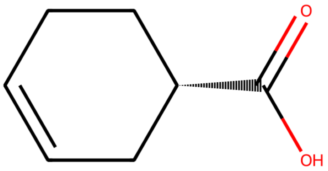

A160

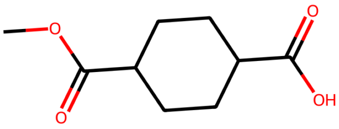

A161

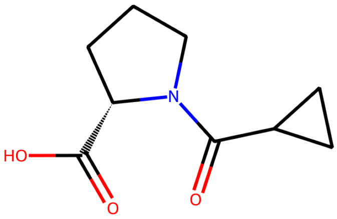

A162

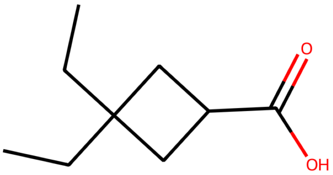

A163

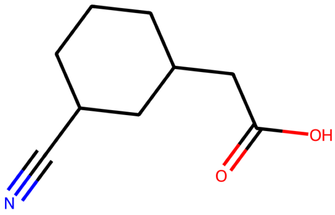

A164

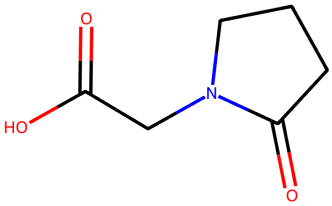

A165

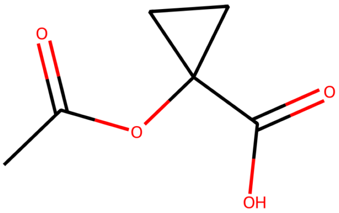

A166

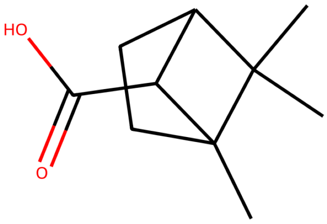

A167

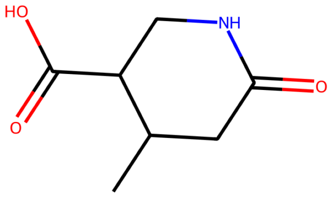

A168

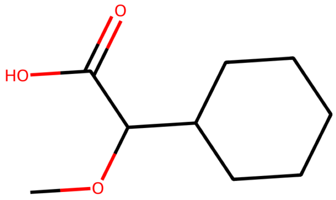

A169

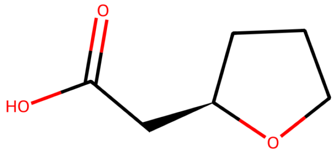

A170

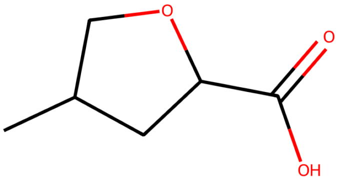

A171

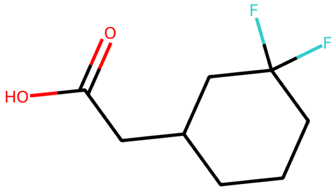

A172

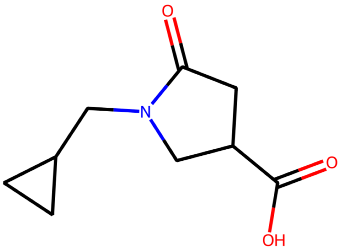

A173

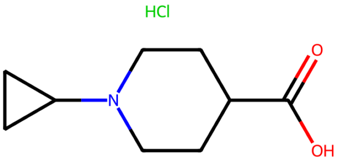

A174

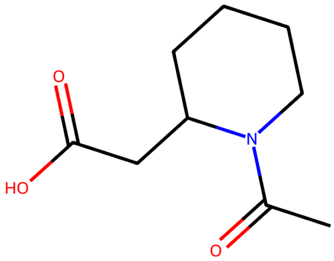

A175

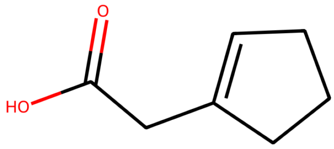

A176

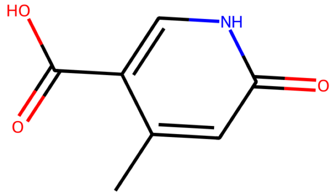

A177

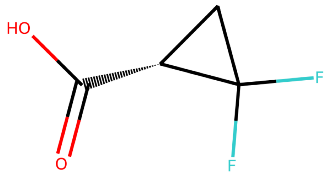

A178

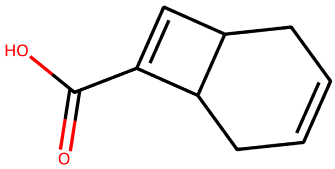

A179

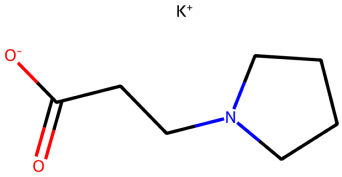

A180

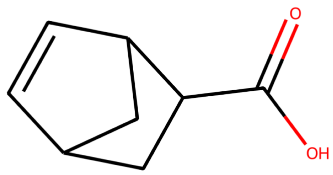

A181

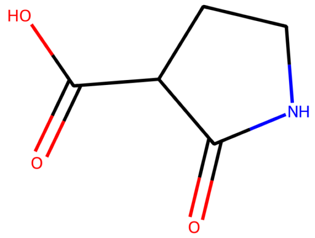

A182

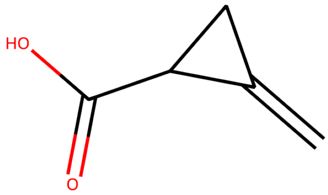

A183

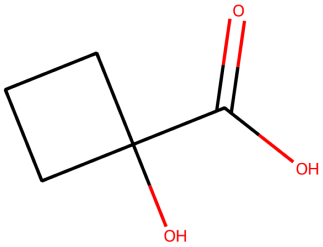

A184

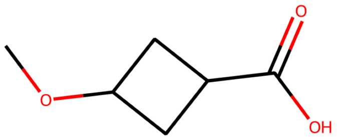

A185

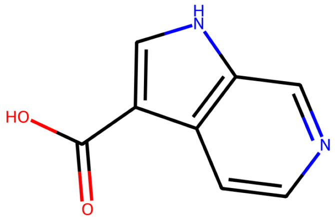

A186

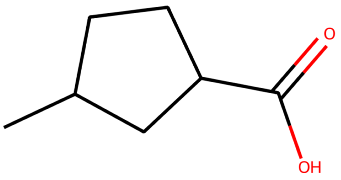

A187

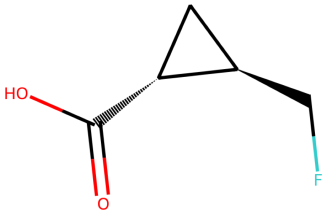

A188

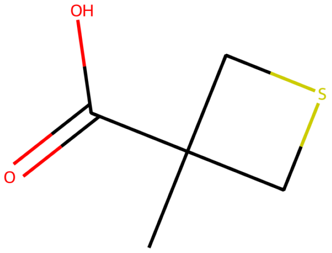

A189

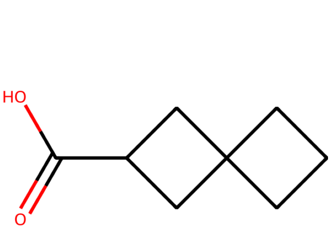

A190

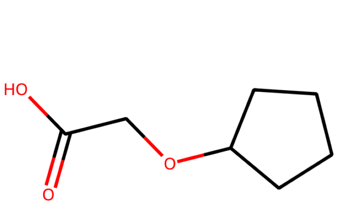

A191

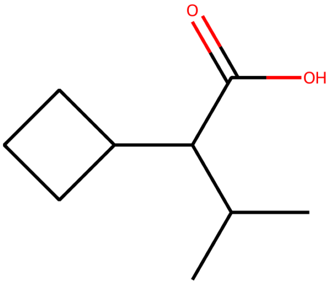

A192

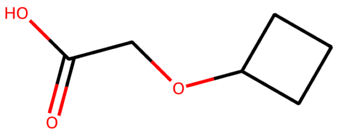

A193

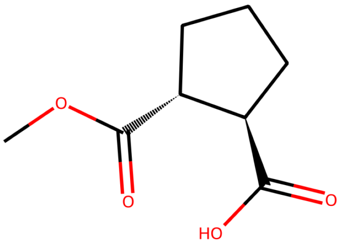

A194

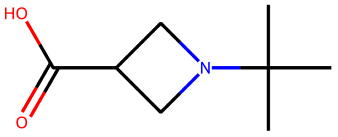

A195

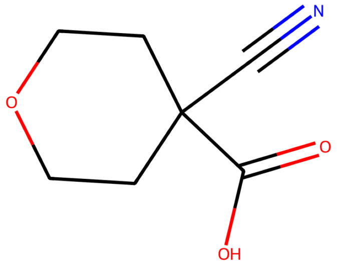

A196

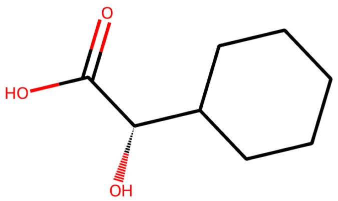

A197

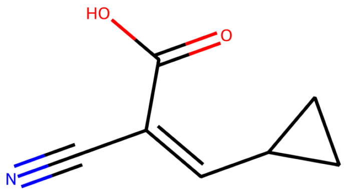

A198

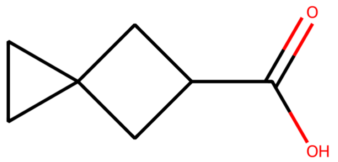

A199

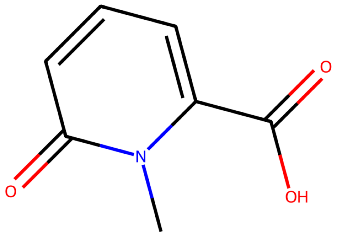

A200

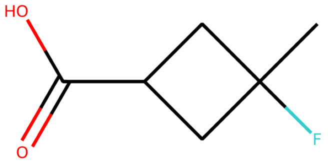

A201

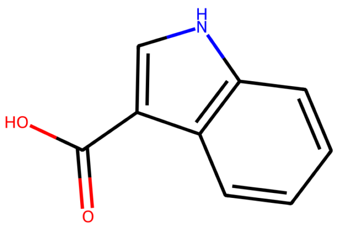

A202

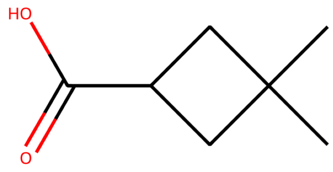

A203

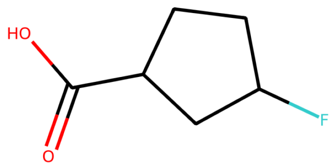

A204

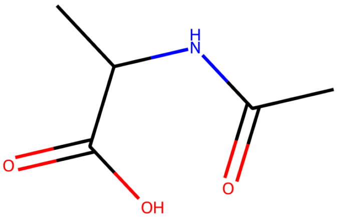

A205

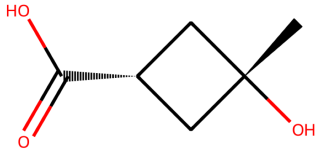

A206

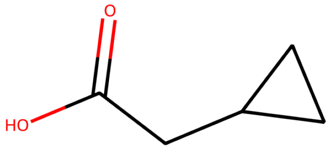

A207

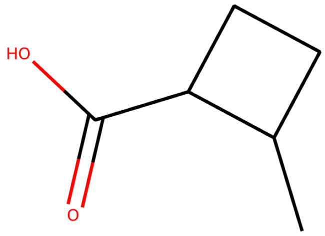

A208

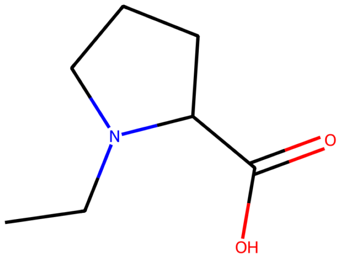

A209

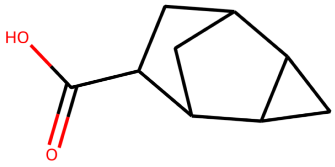

A210

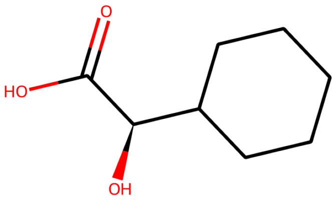

A211

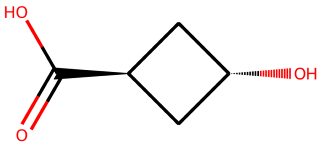

A212

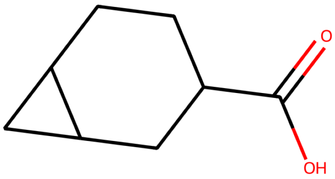

A213

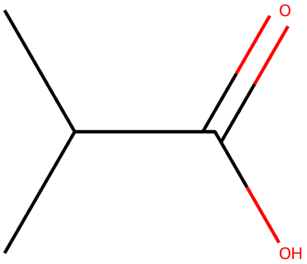

A214

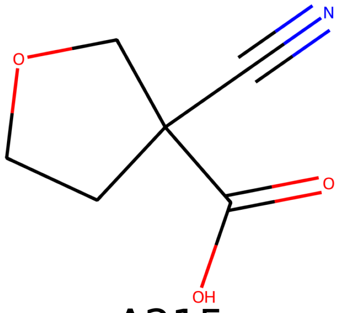

A215

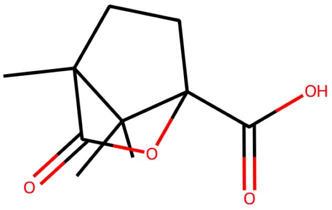

A216

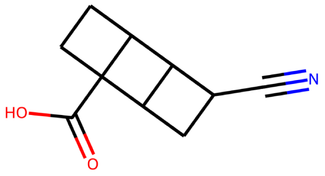

A217

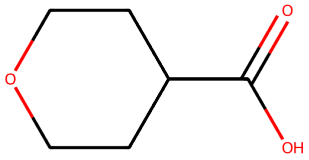

A218

SI5 Scale-up reactions

SI5.1 Reagent and purification information

Unless otherwise noted, reactions were conducted under air in a normal atmosphere. All chemicals were purchased from Sigma Aldrich (St. Louis, US), AstaTech (Bristol, US), Combi-Blocks (San Diego, US), TRC (Toronto, CA), Thermo Scientific (Waltham, US) or obtained from the Roche compound library and used as received. The vials were heated on stirring plates from IKA (Staufen, DE) or Radleys (Saffron Walden, UK) and the reaction mixture was stirred by VP 721F-1 Parylene Encapsulated Stainless Steel Stir Discs from V&P Scientific Inc. (San Diego, US). Purification by flash column chromatography was performed using SiliaSep Premium Flash Cartridges from Silicycle (Quebec, CA) on a Combi Flash Rf from Teledyne ISCO (Nebraska, US) or by reversed-phase high-pressure liquid chromatography (RP-HPLC) on a Gilson (Middleton, USA) GX-281 liquid handler equipped with a Shimadzu (Kyoto, JP) LC-20AP dual pump, a Thermo Fisher Scientific (Waltham, US) UV/VIS-Thermo ultimate 300 Detector, a VWR (Radnor, US) ELSD90 ELSD detector and a Thermo Fisher Scientific (Waltham, US) Thermo MSQ Plus MS Single Quadrupole using a Phenomenex (Torrance, US) Gemini NX C18 column (12 nm, 5 um silica, 30 mm diameter, 100 mm length, flow rate of 40 mL/min) or YMC (Kyoto, JP) Triart C18 (12 nm, 5 um, 100x30 mm) column. The eluent solvents, gradients, and cartridge sizes used for flash chromatography and RP-HPLC are described individually for each experiment.

SI5.2 Analytical information

All novel compounds were characterized by nuclear magnetic resonance (NMR) spectroscopy and (flow injection analysis (FIA)) high-resolution mass spectrometry (HRMS) or gas-chromatography mass spectrometry (GCMS). NMR spectra were recorded on a Bruker Avance III, 600 MHz spectrometer equipped with a 5 mm TCI, Z-gradient CryoProbe, a Bruker Avance Neo, 400 MHz spectrometer equipped with a 5 mm Z-gradient iProbe or a Bruker Avance III HD, 300 MHz spectrometer equipped with a 5 mm BBI-Probe. NMR data are reported as follows: chemical shift in reference to the residual solvent peak (δ ppm), mutiplicity (s = singlet, d = doublet, br d = broad doublet, dd = doublet of doublet, br dd = broad doublet of doublet, t = triplet, br t = broad triplet, m = mutiplet), coupling constant (Hz), and integration. ^{1}H NMR residual solvent peaks in respective deuterated solvents for $CHCl_3$ at 7.26 ppm and DMSO at 2.50 ppm. ^{13}C NMR residual solvent peaks in respective deuterated solvents for $CHCl_3$ at 77.16 ppm and DMSO at 39.52 ppm.

LC-MS high-resolution spectra were recorded with an Agilent LC system consisting of an Agilent 1290 high-pressure gradient system, and an Agilent 6545 QTOF. The separation was achieved on a Zorbax Eclipse Plus C18 1.7 µm 2.1 x 50 mm column (P/N 959731-902) at 55 °C; A: 0.01% HCOOH in H_2O; B: MeCN at flow 0.8 mL/min. Gradient: 0 min 5% B, 0.3 min 5% B, 4.5 min 99% B, 5 min 99% B. The injection volume was 2 µL. Ionization was performed in an Agilent Mutimode source. The mass spectrometer was run in “2 GHz extended dynamic range” mode, resuting in a resolution of about 20,000 at m/z = 922. Mass accuracy was ensured by internal drift correction. GC-MS spectra were recorded on an Agilent 5975B single quadrupole mass spectrometer. Separation was achieved on an Agilent 7890A using an HP-1ms column (15 m ID: 250 µm and 0.25 µm film) with He as carrier gas. Sample introduction was done via a Split injector at 270 °C. After 0.5 min at a constant temperature, the temperature was ramped from 100 °C or 45 °C to 320 °C with 35 °C/min. The mass spectrometer was operated in EI (electron ionization) mode at 70 eV. FIA-HRMS spectra were recorded with an Agilent LC system consisting of an Agilent 1290 high-pressure gradient system, and an Agilent 6540 QTOF. No separation was intended and the injected sample was flushed directly into the Agilent Jetstream source. The mass spectrometer was run in “2 GHz extended dynamic range” mode, resuting in a resolution of about 20,000 at m/z 922. Mass accuracy was ensured by internal drift correction.

SI5.3 Experimental procedures and analytical data

SI5.3.1 Synthesis of head groups

***tert*-Butyl 4-(4-oxo-3 H-quinazolin-2-yl)piperazine-1-carboxylate** (**13a**):

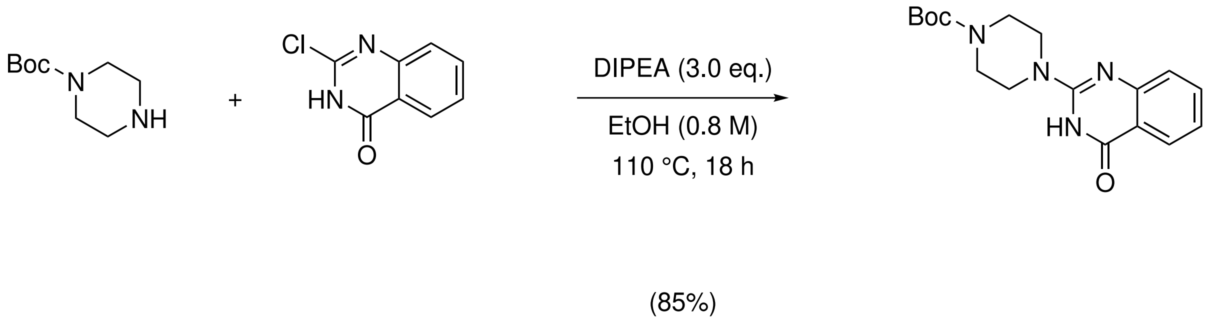

13a

Figure S3: Synthesis of precursor for **13** (**13a**).

2-Chloroquinazolin-4(3H)-one (3.0 g, 13.3 mmol, 1.0 eq.) was suspended in EtOH (16.9 mL) at RT, whereupon *tert*-Butyl piperazine-1-carboxylate (2.53 g, 13.3 mmol, 1.0 eq.) and DIPEA (5.15 g, 6.96 mL, 39.9 mmol, 3.0 eq.) were added. The reaction mixture was purged with argon and stirred in a sealed tube at 110 °C for 18 hours. The reaction mixture was concentrated *in vacuo* with silica and purified by flash column chromatography (SiO_2, 120 g, Eluent: DCM:MeOH, 100:0 to 90:10 over 40 min). Fractions containing the product were combined and concentrated *in vacuo* to yield *tert*-Butyl 4-(4-oxo-3 H-quinazolin-2-yl)piperazine-1-carboxylate (**13a**, 3.75 g, 85%) as an off-white solid.

^{1}H NMR (300 MHz, DMSO-d_6) δ (ppm) 11.38 (br s, 1H), 7.91 (dd, J = 8.0, 1.3 Hz, 1H), 7.59 (ddd, J = 8.4, 7.1, 1.7 Hz, 1H), 7.30 (d, J = 7.9 Hz, 1H), 7.16 (t, J = 7.5 Hz, 1H), 3.61 (dd, J = 6.3, 3.9 Hz, 4H), 3.46 – 3.36 (m, 4H), 1.42 (s, 9H).
LC-MS $C_{17}H_{22}N_4O_3$; calc. for (M+H$^+$): 331.2, found: 331.3.

2-Piperazin-1-yl-3 H-quinazolin-4-one dihydrochloride (13):

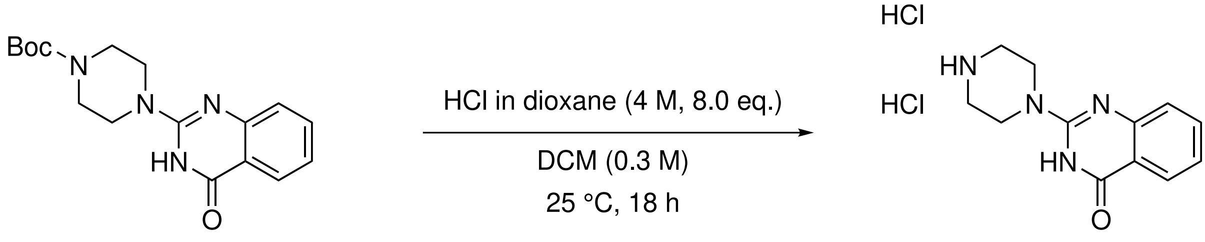

(97%)

13a **13**

Figure S4: Synthesis of head group 1 (**13**).

tert-Butyl 4-(4-oxo-3,4-dihydroquinazolin-2-yl)piperazine-1-carboxylate (**13a**, 3.74 g, 11.3 mmol, 1.0 eq.) was suspended in DCM (40 mL) at RT, whereupon HCl in ioxane (22.6 mL, 90.6 mmol, 8.0 eq.) was added. The reaction mixture was then stirred at RT for 18 hours. The reaction mixture was concentrated *in vacuo* and triturated with hexane, filtered and dried over high vacuum to give 2-piperazin-1-yl-3 H-quinazolin-4-one dihydrochloride (**13**, 3.34 g, 97%) as a light brown solid.

^{1}H NMR (600 MHz, DMSO-*d$_6$*) δ (ppm) 9.31 (br s, 1H), 7.97 (dd, J = 7.9, 1.3 Hz, 1H), 7.69 (br t, J = 7.7 Hz, 1H), 7.52 (br s, 1H), 7.28 (t, J = 6.9 Hz, 1H), 3.92 (br s, 4H), 3.22 (br s, 4H).
HRMS $C_{12}H_{14}N_4O$; calc. for $(M+H^+)$: 231.1240, found: 231.1244.

5-Chloro-2-methylsufanyl-oxazolo[4,5-b]pyridine (14a):

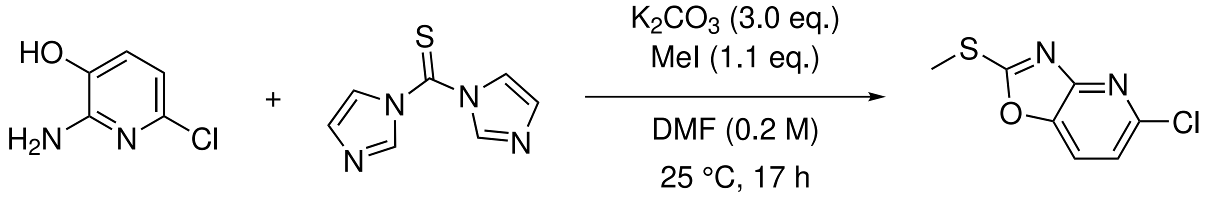

(20%)

14a

Figure S5: Synthesis of precursor 1 for head group 2 (**14a**).

To a solution of di(1H-Imidazol-1-yl)methanethione (1.24 g, 6.94 mmol, 1.0 eq.) in DMF (17.3 mL) under argon atmosphere was added a solution of 2-Amino-6-chloropyridin-3-ol (1003 mg, 6.94 mmol, 1.0 eq.) in DMF and the brown reaction mixture was stirred at rt for 16 hours. K_2CO_3 (1.92 g, 13.9 mmol, 2.0 eq.) and methyl iodide (1.08 g, 477 µL, 7.63 mmol, 1.1 eq.) were added and the reaction mixture was stirred at rt for 1 h. The reaction mixture was concentrated in vacuo (60 °C, 20 mbar) and the resulting residue was dissolved in dichloromethane and washed with water. The aqueous phase was extracted three times with DCM. The combined organic phases were washed with brine, dried over sodium sulfate, stored overnight and evaporated to dryness to give a brown solid (590 mg). The crude material was purified by flash chromatography with a 40 g SiO_2 column, eluent mixture of heptane and ethyl acetate (0% to 30%) to afford 5-Chloro-2-(methylthio)oxazolo[4,5-b]pyridine (**14a**, 275 mg, 1.36 mmol, 20% yield) as a white solid.

^{1}H NMR (300 MHz, $CDCl_3$) δ (ppm) 7.66 (d, J = 8.5 Hz, 1H), 7.21 (d, J = 8.5 Hz, 1H), 2.81 (s, 3H).
LC-MS $C_7H_5ClN_2OS$; calc. for $(M+H^+)$: 201.0, found: 201.1.

***tert*-Butyl 4-(5-chlorooxazolo[4,5-b]pyridin-2-yl)piperazine-1-carboxylate** (**14b**):

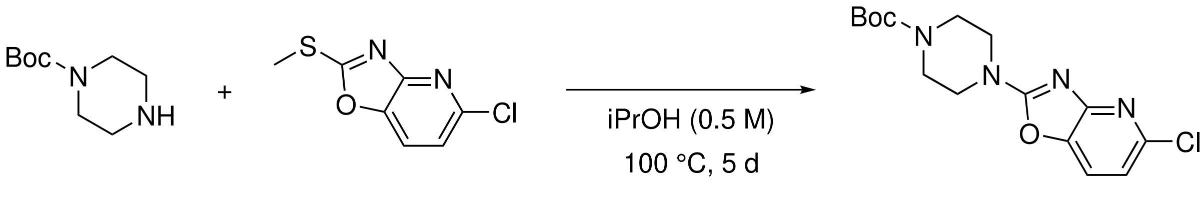

(89%)

14a **14b**

Figure S6: Synthesis of precursor 2 for head group 2 (**14b**).

To a solution of 5-Chloro-2-(methylthio)oxazolo[4,5-b]pyridine (**14a**, 150 mg, 748 µmol, 1.0 eq.) in iPrOH (1.5 mL) was added *tert*-Butyl piperazine-1-carboxylate (209 mg, 1.12 mmol, 1.5 eq.) and the reaction mixture was stirred at 100 °C for 4 days in a sealed vial. The volatiles were removed in vacuo and the resulting light-yellow solid (314 mg) was directly purified by flash chromatography with a 25 g SiO_2 column, eluent mixture of dichloromethane and methanol (0% to 10%) to afford *tert*-Butyl 4-(5-chlorooxazolo[4,5-b]pyridin-2-yl)piperazine-1-carboxylate (**14b**, 228.0 mg, 666 µmol, 89% yield) as an off-white solid.

^{1}H NMR (300 MHz, $CDCl_3$) δ (ppm) 7.39 (d, J = 8.3 Hz, 1H), 6.95 (d, J = 8.1 Hz, 1H), 3.81 – 3.67 (m, 4H), 3.64 – 3.53 (m, 4H), 1.50 (s, 9H).
LC-MS $C_{15}H_{19}ClN_4O_3$; calc. for $(M+H^+)$: 339.1, found: 339.2.

5-Chloro-2-piperazin-1-yl-oxazolo[4,5-b]pyridine hydrochloride (**14**):

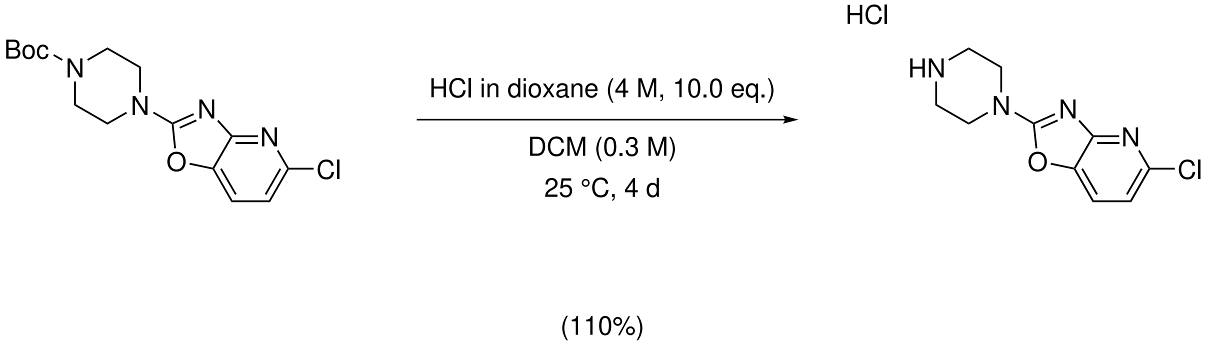

14b **14**

Figure S7: Synthesis of head group 2 (**14**).

To *tert*-Butyl 4-(5-Chlorooxazolo[4,5-b]pyridin-2-yl)piperazine-1-carboxylate (**H2b**, 228 mg, 673 µmol, 1.0 eq.) in dioxane (2.24 mL) was added hydrochloric acid solution 4M in dioxane (1.35 mL, 5.38 mmol, 8.0 eq.) at rt, and the reaction mixture was stirred for 4 days. A non-stirrable slurry formed shortly after addition and dioxane (1.12 mL) was added. 4M Hydrochloric acid solution in dioxane (336 u, 1.35 mmol, 2.0 eq.) was added. The volatiles were removed to afford 5-Chloro-2-(piperazin-1-yl)oxazolo[4,5-b]pyridine hydrochloride (**14**, 215 mg, 742 µmol, 110% yield) as a white solid. The crude was used without further purification.

^{1}H NMR (300 MHz, DMSO-d$_6$) δ (ppm) 9.54 (br s, 2H), 7.87 (d, J = 8.3 Hz, 1H), 7.13 (d, J = 8.1 Hz, 1H), 6.57 (br s, 3H), 3.98 – 3.87 (m, 4H), 3.27 (br s, 4H).
LC-MS $C_{10}H_{11}ClN_4O$; calc. for (M+H$^+$): 239.0794, found: 239.1.

***tert*-Butyl 6-((methylsulfonyl)oxy)-2-azaspiro[3.3]heptane-2-carboxylate** (**15a**):

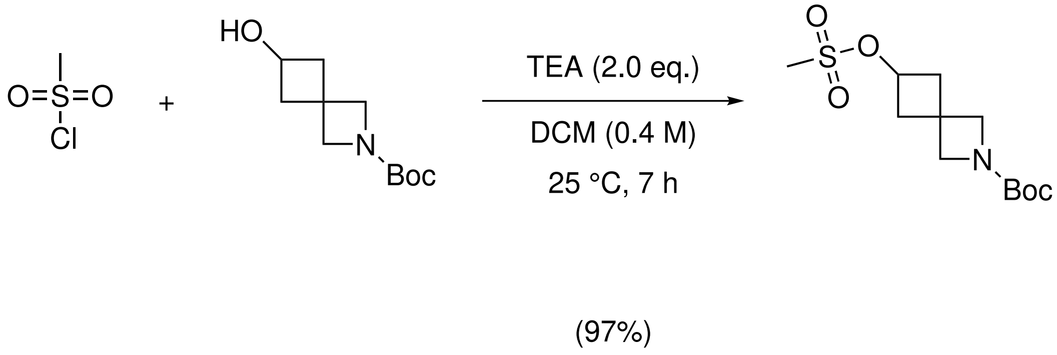

15a

Figure S8: Synthesis of precursor 1 for head group 3 (**15a**).

To a solution of *tert*-Butyl 6-hydroxy-2-azaspiro[3.3]heptane-2-carboxylate (5.00 g, 23.4 mmol, 1.0 eq.) in dry DCM (59.0 mL) was added triethylamine (4.74 g, 6.54 mL, 46.9 mmol, 2.0 eq.). The mixture was cooled to 0 °C and methanesulfonyl chloride (2.95 g, 2.00 mL, 25.8 mmol, 1.1 eq.) was added. The reaction mixture was stirred for 7 h. The mixture was washed with water (2x) and brine (1x). The organic phases were combined and dried over Na_2SO_4. Evaporation of the solvent gave the title compound *tert*-Butyl 6-((methylsulfonyl)oxy)-2-azaspiro[3.3]heptane-2-carboxylate (**15a**, 6.58 g, 22.6 mmol, 97%), which was obtained as a yellow-orange solid.

^{1}H NMR (300 MHz, $CDCl_3$) δ (ppm) 4.89 (quin, J = 7.3 Hz, 1H), 3.93 (s, 4H), 2.99 (s, 3H), 2.76 – 2.65 (m, 2H), 2.53 – 2.42 (m, 2H), 1.43 (s, 9H).
LC-MS $C_{12}H_{22}NO_5S^+$; calc. for ($M+H^+$): 292.1, found: 236.2 $[M-C_4H_9+H]^+$

***tert*-Butyl 6-(3-cyclopropyl-1H-1,2,4-triazol-1-yl)-2-azaspiro[3.3]heptane-2- carboxylate** (**15b**):

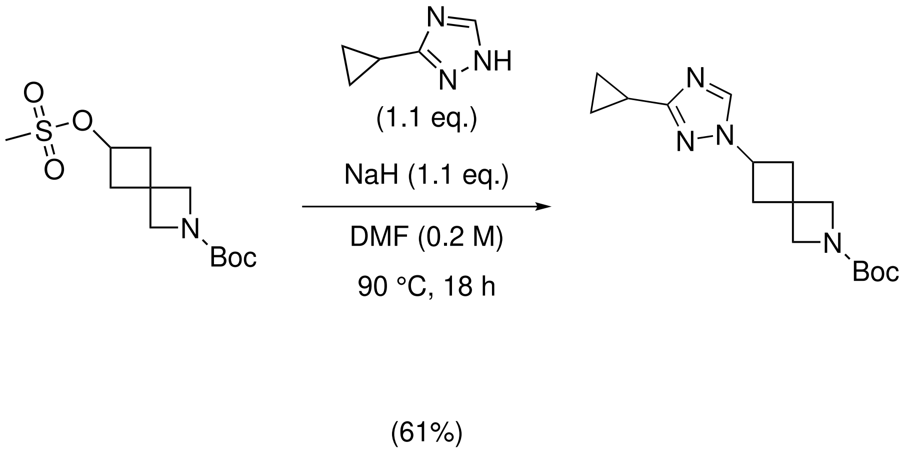

15a **15b**

Figure S9: Synthesis of precursor 2 for head group 3 (**15b**).

A solution of 3-Cyclopropyl-1H-1,2,4-triazole (948 mg, 8.68 mmol, 1.1 eq.) in dry DMF (36.7 mL) was stirred at 0 °C for 5 min, followed by addition of NaH (347 mg, 8.68 mmol, 1.1 eq.). The reaction mixture was stirred at rt for 20 min followed by addition of *tert*-Butyl 6-((methylsulfonyl)oxy)-2-azaspiro[3.3]heptane-2-carboxylate (**15a**, 2.30 g, 7.89 mmol, 1.0 eq.). The reaction mixture was stirred at 90 °C for 18 h, quenched by addition of sat. aq. NH_4Cl, diluted with EtOAc and washed with 1M aq. $NaHCO_3$ solution. The organic phase was collected and the aqueous phase was back extracted with EtOAc. The combined organic phases were dried over Na_2SO_4 and evaporated to dryness. To separate the two regioisomers, the crude material was purified by SFC (7-20% MeOH, t_{ret} = 2.44 min). Evaporation of the solvent gave the title compound, *tert*-butyl 6-(3-cyclopropyl-1H-1,2,4-triazol-1-yl)-2-azaspiro[3.3]heptane-2-carboxylate (**15b**, 1.46 g, 4.80 mmol, 61%), which was obtained as a yellow viscous oil .

^{1}H NMR (300 MHz, $CDCl_3$) δ (ppm) 7.84 (s, 1H), 4.61 (t, J = 8.0 Hz, 1H), 4.00 (d, J = 13.9 Hz, 4H), 2.82 – 2.66 (m, 4H), 2.05 (quin, J = 1.0 Hz, 1H), 1.45 (s, 9H), 0.99 – 0.92 (m, 4H).
LC-MS $C_{16}H_{25}N_4O_2$; calc. for (M+H$^+$): 305.2, found: 305.3.

6-(3-Cyclopropyl-1H-1,2,4-triazol-1-yl)-2-azaspiro[3.3]heptane 2,2,2-trifluoroacetate (**15**):

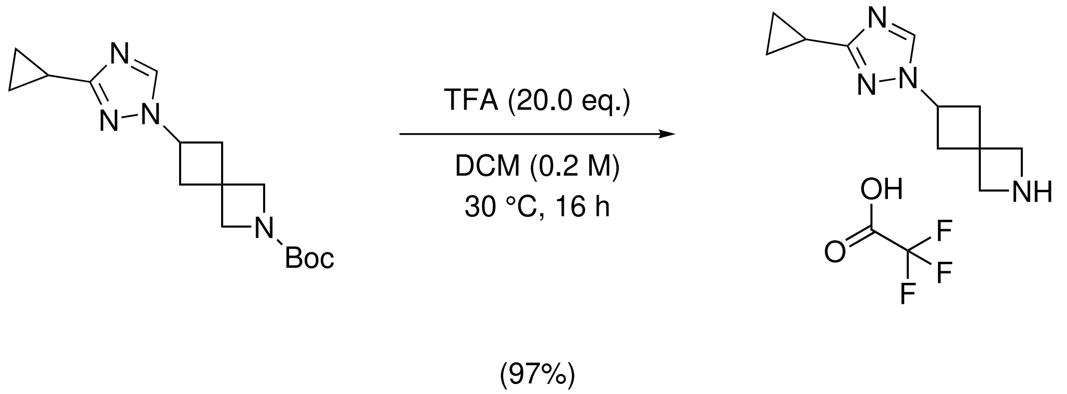

15b **15**

Figure S10: Synthesis of head group 3 (**15**).

To a solution of tert-butyl 6-(3-cyclopropyl-l,2,4-triazol-l-yl)-2-azaspiro[3.3]heptane-2- carboxylate (**15b**, 6.00 g, 19.7 mmol) in DCM (120 mL) was added TFA (46.2 g, 405 mmol, 30 mL) at 25 °C. The mixture was stirred at 30 °C for 16 h, before being evaporated. The title compound (**15**, 14.0 g, crude) was used in the next step without further purification.

^{1}H NMR (600 MHz, DMSO-d$_6$) δ (ppm) 8.88 – 8.33 (m, 2H), 8.31 (s, 1H), 4.75 (quin, J = 7.9 Hz, 1H), 4.07 – 3.98 (m, 4H), 2.77 – 2.71 (m, 2H), 2.65 – 2.59 (m, 2H), 1.95 (tt, J = 8.2, 4.8 Hz, 1H), 0.92 – 0.83 (m, 2H), 0.80 – 0.71 (m, 2H)

HRMS $C_{11}H_{16}N_4$; calc. for $(M+H^+)$: 205.1448, found: 205.1451.

1-(1-benzhydrylazetidin-3-yl)piperazine (16a):

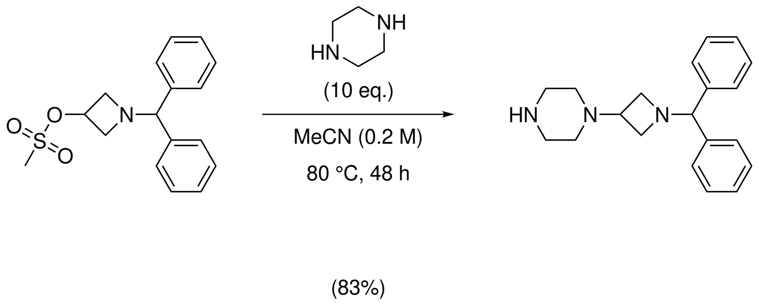

16a

Figure S11: Synthesis of precursor 1 for head group 4 (**16a**).

To a solution of Piperazine (2.71 g, 31.5 mmol, 10.0 eq.) in MeCN (15 mL) was added 1-Benzhydrylazetidin-3-yl methanesulfonate (1 g, 3.15 mmol, 1.0 eq.) and the reaction mixture was heated to 80 °C and stirred at this temperature for 48 hours. After 48 h, the reaction mixture was worked up by concentrating to half volume and filtered to remove trace amounts of white solid. The filtrate was poured into DCM and water and extracted with DCM (3 x 40 mL, owing to a difficult-to-see separation) before the DCM was then washed with water (3 x 50 mL), dried over Mg_2SO_4 and concentrated *in vacuo* to yield 1.88 g of an orange liquid. After drying on the high vacuum overnight, 1-(1-benzhydrylazetidin-3-yl)piperazine (**16a**, 804 mg, 2.61 mmol, 83%) was obtained as a viscous orange oil.

^{1}H NMR (600 MHz, CDCl$_3$) δ (ppm) 7.44 – 7.36 (m, 4H), 7.31 – 7.27 (m, 2H), 7.27 – 7.24 (m, 2H), 7.22 – 7.16 (m, 2H), 4.42 (s, 1H), 3.43 – 3.36 (m, 2H), 3.02 – 2.82 (m, 6H), 2.35 – 2.18 (m, 3H), 2.10 – 1.97 (m, 1H).
HRMS $C_{20}H_{25}N_3$; calc. for (M+H$^+$): 308.2121, found: 308.2149.

[4-(1-Benzhydrylazetidin-3-yl)piperazin-1-yl]-thiazol-2-yl-methanone (**16b**):

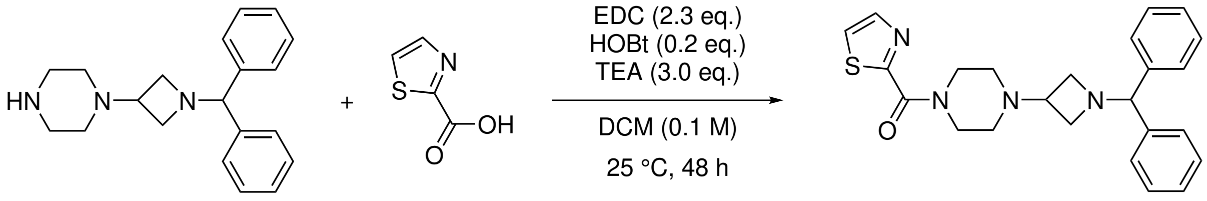

(60%)

16a **16b**

Figure S12: Synthesis of precursor 2 for head group 4 (**16b**).

1-(1-Benzhydrylazetidin-3-yl)piperazine (**16a**, 286 mg, 929 µmol, 1.3 eq.) was dissolved in DCM (5.27 mL), whereupon Thiazole-2-carboxylic acid (96 mg, 743 µmol, 1.0 eq.), HOBT (23.9 mg, 156 µmol, 0.2 eq.), EDC.HCl (332 mg, 1.73 mmol, 2.3 eq.) and triethylamine (226 mg, 311 µL, 2.23 mmol, 3.0 eq.) were added. The reaction was left to stir at RT over the weekend. The reaction was then worked up by pouring into a separating funnel containing sat. $NaHCO_3$ and DCM. The mixture was extracted with DCM and washed with brine, dried over Mg_2SO_4 and concentrated *in vacuo* with silica. The mixture was then purified via flash column chromatography (SiO_2, 25 g, Eluent: Hep:EtOAc 80:20 to 100% EtOAc over 25 min.). Fractions containing the product were combined and concentrated to yield 4-(1-Benzhydrylazetidin-3-yl)piperazin-1-yl]-thiazol-2-yl-methanone (**16b**, 231 mg, 552 µmol, 60%) as a white crystalline solid after drying under a high vacuum.

^{1}H NMR (300 MHz, $CDCl_3$) δ (ppm) 7.87 (d, J = 3.2 Hz, 1H), 7.53 (d, J = 3.2 Hz, 1H), 7.42 (br d, J = 7.3 Hz, 4H), 7.33 – 7.27 (m, 3H), 7.26 (br s, 1H), 7.24 – 7.14 (m, 2H), 4.13 (q, J = 7.1 Hz, 1H), 3.82 (br s, 2H), 3.52 – 3.31 (m, 2H), 3.08 – 2.85 (m, 3H), 2.39 (br s, 4H), 2.05 (s, 1H), 1.27 (t, J = 7.2 Hz, 1H).
LC-MS $C_{24}H_{26}N_4OS$; calc. for $(M+H^+)$: 419.2, found: 419.2.

[4-(Azetidin-3-yl)piperazin-1-yl]-thiazol-2-yl-methanone (16):

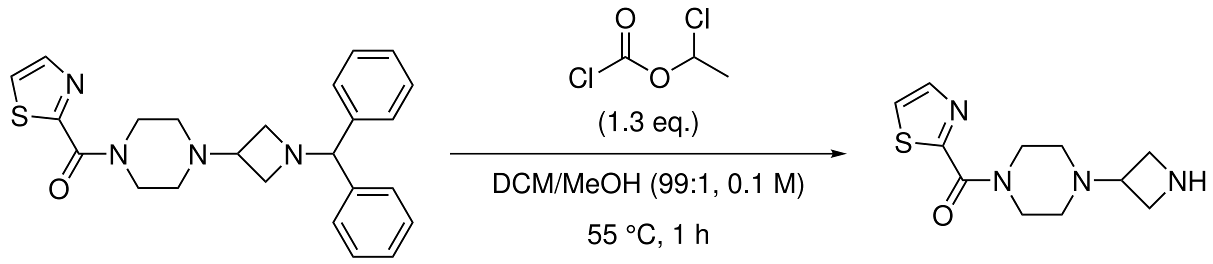

(132%)

16b **16**

Figure S13: Synthesis of head group 4 (**16**).

To a solution of (4-(1-Benzhydrylazetidin-3-yl)piperazin-1-yl)(thiazol-2-yl)methanone (**16b**, 22.2 g, 53 mmol, 1.0 eq.) in DCM (844 mL) at 0 °C, 1-Chloroethyl carbonochloridate (9.86 g, 7.44 mL, 69 mmol, 1.3 eq.) was added. The reaction mixture was stirred for 1.5 h at 0 °C. Methanol was added and the reaction mixture was stirred under reflux (55 °C) for 1 h. The reaction mixture was diluted with MeOH, dried with Na_2SO_4 filtered and the solvent was removed under reduced pressure to afford [4-(azetidin-3-yl)piperazin-1-yl]-thiazol-2-yl-methanone (**16**, 23.1 g, 92.0 mmol, 132%).

^{1}H NMR (300 MHz, DMSO-d$_6$) δ (ppm) 9.61 (br s, 1H), 9.19 (br s, 1H), 8.16 – 7.96 (m, 2H), 4.37 (br s, 4H), 4.16 – 3.98 (m, 4H), 3.29 – 2.97 (m, 5H).
HRMS $C_{11}H_{16}N_4OS$; calc. for $(M+H^+)$: 253.1118, found: 253.1122.

SI5.3.2 Synthesis of adaptable building block

Methyl 2-(3-Pyridyl)-1,3-benzoxazole-6-carboxylate (11):

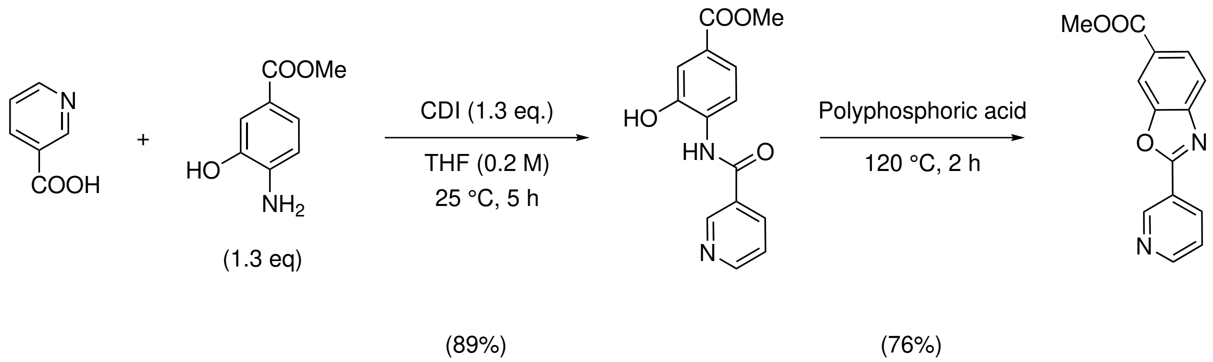

1 **7** **10** **11**

Figure S14: Synthesis of modifiable building block (**11**).

A mixture of Nicotinic acid (766.61 mg, 6.23 mmol, 1.3 eq) and CDI (1.01 g, 6.23 mmol, 1.3 eq) in 20 mL THF was stirred at rt for 0.5 h. Then, 4-Amino-3-hydroxy-benzoic acid methyl ester (800.7 mg, 4.79 mmol, 1.0 eq) was added and the mixture was stirred at 20 °C for 5 h. The reaction mixture was poured into water (30 mL) and extracted with EtOAc. The combined organic layers were washed with water, and brine, dried over Na_2SO_4, filtered and concentrated to dryness. The crude material was dispersed in EtOAc and filtrated. The filter cake contained most of the product. The filtrate was precipitated with water and more product was gained and again filtrated with EtOAc. The combination of the filter cakes gave 3-Hydroxy-4-nicotinamido-benzoic acid methyl ester (**10**, 1.16 g, 4.26 mmol, 89%) as a white solid. Without further characterization, 3-Hydroxy-4-nicotinamido-benzoic acid methyl ester (**10**, 1.16 g, 4.26 mmol, 1.0 eq) and Polyphosphoric acid (3.6 g) were stirred at 120 °C for 2 h. The reaction mixture was diluted with water (30 mL) and extracted with EtOAc. The combined organic layers were washed with water and brine, dried over Na_2SO_4, filtered and concentrated to dryness. The crude material was suspended in DMF and filtrated using Benzene. The filtrate was diluted with water causing more product to precipitate. The product was removed by filtration. Both product fractions were combined to give the title compound 2-(3-Pyridyl)-1,3-benzoxazole-6-carboxylic acid methyl ester (**11**, 836.6 mg, 3.29 mmol, 76%) as an off-white solid.

^{1}H NMR (600 MHz, CDCl$_3$) δ (ppm) 9.56 – 9.52 (m, 1H), 8.87 – 8.82 (m, 1H), 8.65 (d, J = 8.0 Hz, 1H), 8.33 (s, 1H), 8.15 (dd, J = 8.4, 1.5 Hz, 1H), 7.85 (dd, J = 8.4, 0.6 Hz, 1H), 7.63 – 7.57 (m, 1H), 4.00 (s, 3H). **^{13}C NMR (151 MHz, CDCl$_3$)** δ (ppm) 166.6, 162.8, 151.5, 150.7, 148.1, 145.7, 136.3, 128.2, 127.0, 124.5, 123.8, 120.2, 112.8, 52.7. **GC-MS** $C_{14}H_{10}N_2O_3$; calc. for $(M+H^+)$: 255.1, found: 255.1.

SI5.3.3 Synthesis of alkylated building blocks

Methyl 2-(4-cyclopentyl-3-pyridyl)-1,3-benzoxazole-6-carboxylate (9a), methyl 2-(2-cyclopentyl-3-pyridyl)-1,3-benzoxazole-6-carboxylate (9b), methyl 2-(6-cyclopentyl-3-pyridyl)-1,3-benzoxazole-6-carboxylate (9c):

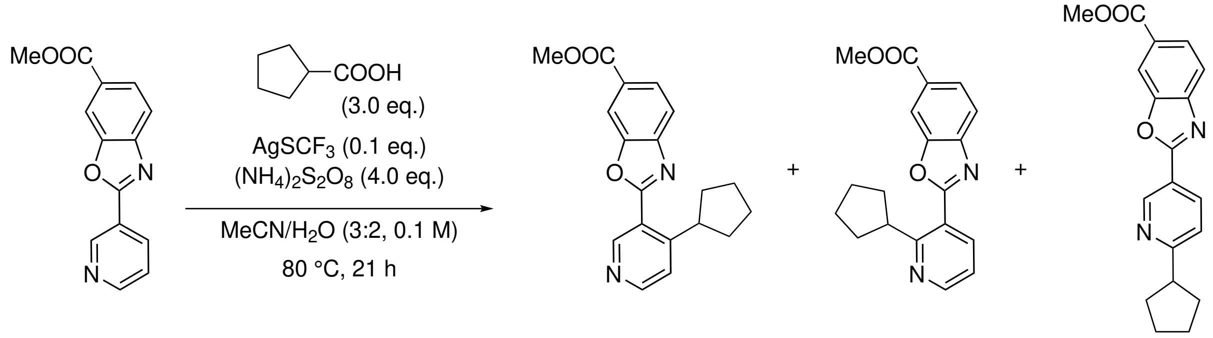

(9%) (1%) (1%)

11 **9a** **9b** **9c**

Figure S15: Synthesis of modifiable building blocks (**9a, 9b, 9c**).

A mixture of 2-(3-Pyridyl)-1,3-benzoxazole-6-carboxylic acid methyl ester (**1**, 400 mg, 1.57 mmol, 1.0 eq), Cyclopentanecarboxylic acid (539 mg, 4.72 mmol, 3.0 eq), Ammonium persulfate (1.08 g, 4.72 mmol, 3.0 eq) and Silver trifluoromethanethiolate (32.87 mg, 157 µmol, 0.1 eq) in 9.4 mL degassed MeCN and 6.3 mL degassed water was stirred at 80 °C for 19 h. Afterwards, an additional 1.0 eq of Ammonium persulfate was added and the mixture was stirred for an additional 2 h at 80 °C. The reaction mixture was quenched with sat. $NaHCO_3$ solution and extracted with EtOAc and precipitation of a solid was observed. The combined organic layers were filtered, washed with brine, dried over Na_2SO_4, filtered and concentrated to dryness. The crude material was purified by silica gel column chromatography (12 g) using an EtOAc gradient (0%-80%) in heptane. The solvent was evaporated from the product containing fractions. Product fractions were combined to give the title compounds 2-(4-Cyclopentyl-3-pyridyl)-1,3-benzoxazole-6-carboxylic acid methyl ester (**9a**, 44.1 mg, 136 µmol, 9%) as white solid, 2-(2-Cyclopentyl-3-pyridyl)-1,3-benzoxazole-6-carboxylic acid methyl ester (**9b**, 6.7 mg, 20.7 µmol, 1%) as white solid and 2-(6-Cyclopentyl-3-pyridyl)-1,3-benzoxazole-6-carboxylic acid methyl ester (**9c**, 7.2 mg, 21.7 µmol, 1%) as white solid.

9a:
^{1}H NMR (600 MHz, $CDCl_3$) δ (ppm) 9.25 (s, 1H), 8.67 (d, J = 5.3 Hz, 1H), 8.33 (d, J = 1.5 Hz, 1H), 8.15 (dd, J = 8.4, 1.5 Hz, 1H), 7.86 (d, J = 8.4 Hz, 1H), 7.44 (d, J = 5.3 Hz, 1H), 4.22 – 4.15 (m, 1H), 3.99 (s, 3H), 2.24 – 2.17 (m, 2H), 1.90 – 1.85 (m, 2H), 1.82 – 1.76 (m, 2H), 1.70 – 1.63 (m, 2H). **HRMS** $C_{19}H_{18}N_2O_3$; calc. for ($M+H^+$): 323.1390, found: 323.1387.

9b:
^{1}H NMR (600 MHz, $CDCl_3$) δ (ppm) 8.75 (dd, J = 4.7, 1.8 Hz, 1H), 8.35 (dd, J = 7.9, 1.8 Hz, 1H), 8.32 (d, J = 2.1 Hz, 1H), 8.14 (dd, J = 8.4, 1.5 Hz, 1H), 7.86 (dd, J = 8.3, 0.6 Hz, 1H), 7.29 (dd, J = 7.9, 4.7 Hz, 1H), 4.29 (t, J = 8.4 Hz, 1H), 3.99 (s, 3H), 2.18 – 2.09 (m, 2H), 2.03 – 1.85 (m, 4H), 1.81 – 1.70 (m, 2H). **HRMS** $C_{19}H_{18}N_2O_3$; calc. for ($M+H^+$): 323.1390, found: 323.1387.

9c:
^{1}H NMR (600 MHz, $CDCl_3$) δ (ppm) 9.41 (dd, J = 2.2, 0.7 Hz, 1H), 8.43 (dd, J = 8.2, 2.3 Hz, 1H), 8.31 (d, J = 2.1 Hz, 1H), 8.12 (dd, J = 8.3, 1.5 Hz, 1H), 7.81 (dd, J = 8.4, 0.6 Hz, 1H), 7.38 (d, J = 7.8 Hz, 1H), 3.99 (s, 3H), 3.30 (t, J = 8.3 Hz, 1H), 2.18 – 2.12 (m, 2H), 1.94 – 1.82 (m, 4H), 1.79 – 1.73 (m, 2H). **HRMS** $C_{19}H_{18}N_2O_3$; calc. for ($M+H^+$): 323.1390, found: 323.1386.

methyl 2-[4-(1-Benzyloxycarbonyl-4-piperidyl)-3-pyridyl]-1,3-benzoxazole-6-carboxylate (9d), methyl 2-[2-(1-Benzyloxycarbonyl-4-piperidyl)-3-pyridyl]-1,3-benzoxazole-6-carboxylate (9e):

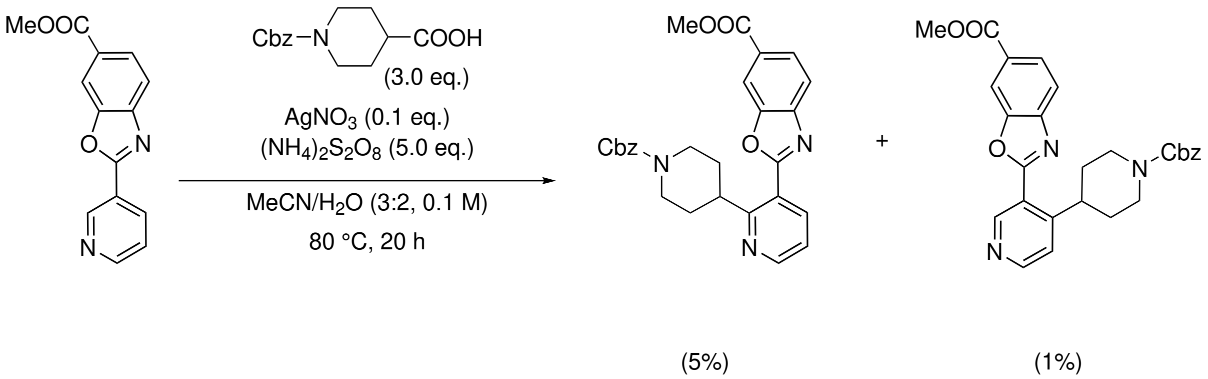

11 **9d** **9e**

Figure S16: Synthesis of modifiable building blocks (**9d, 9e**).

A mixture of 2-(3-Pyridyl)-1,3-benzoxazole-6-carboxylic acid methyl ester (**11**, 400 mg, 1.57 mmol, 1.0 eq), 1-carbobenzoxyisonipecotic acid (1.24 g, 4.72 mmol, 3.0 eq), Ammonium persulfate (1.08 g, 4.72 mmol, 3.0 eq) and Silver nitrate (26.73 mg, 157.33 µmol, 0.1 eq) in 9.4 mL degassed MeCN and 6.3 mL degassed water was stirred at 80 °C for 20 h. After 3 h and 19 h additional 1.0 eqs of Ammonium persulfate were added. The reaction mixture was quenched with sat. $NaHCO_3$ solution and extracted with EtOAc. The combined organic layers were washed with brine, dried over Na_2SO_4, filtered and concentrated to dryness. The crude material was purified by silica gel column chromatography (12 g) using an EtOAc gradient (0%-80%) in heptane. The solvent was evaporated from product containing fractions. Product fractions were combined to give the title compounds 2-[4-(1-Carbobenzoxy-4-piperidyl)-3-pyridyl]-1,3-benzoxazole-6-carboxylic acid methyl ester (**9d**, 55.7 mg, 118 µmol, 5%) as light yellow solid and 2-[2-(1-Carbobenzoxy-4-piperidyl)-3-pyridyl]-1,3-benzoxazole-6-carboxylic acid methyl ester (**9e**, 5.6 mg, 11.8 µmol, 1%) as light yellow solid.

9d:
^{1}H NMR (600 MHz, $CDCl_3$) δ (ppm) 9.36 (s, 1H), 8.71 (d, J = 5.2 Hz, 1H), 8.35 – 8.33 (m, 1H), 8.16 (dd, J = 8.3, 1.5 Hz, 1H), 7.85 – 7.83 (m, 1H), 7.42 – 7.30 (m, 28H), 5.14 (s, 2H), 4.49 – 4.34 (m, 2H), 4.00 (s, 3H), 3.06 – 2.93 (m, 4H), 2.54 (tt, J = 10.8, 3.9 Hz, 1H), 1.91 – 1.84 (m, 2H). **HRMS** $C_{27}H_{25}N_3O_5$; calc. for $(M+H^+)$: 472.1867, found: 472.1859.

9e:
^{1}H NMR (600 MHz, $CDCl_3$) δ (ppm) 8.74 (dd, J = 4.7, 1.8 Hz, 1H), 8.44 (dd, J = 8.0, 1.8 Hz, 1H), 8.31 (d, J = 2.1 Hz, 1H), 8.15 (dd, J = 8.3, 1.5 Hz, 1H), 7.85 (dd, J = 8.3, 0.6 Hz, 1H), 7.42 – 7.30 (m, 6H), 5.17 (br d, J = 4.9 Hz, 2H), 4.53 – 4.28 (m, 2H), 4.20 (tt, J = 11.3, 3.8 Hz, 1H), 4.00 (s, 3H), 3.16 – 2.91 (m, 2H), 2.11 – 1.87 (m, 4H). **HRMS** $C_{27}H_{25}N_3O_5$; calc. for $(M+H^+)$: 472.1867, found: 472.1862.

Methyl 2-[6-(1-Cyclobutylcyclopropyl)-3-pyridyl]-1,3-benzoxazole-6-carboxylate (9f), Methyl 2-[2-(1-Cyclobutylcyclopropyl)-3-pyridyl]-1,3-benzoxazole-6-carboxylate (9g), Methyl 2-[4-(1-Cyclobutylcyclopropyl)-3-pyridyl]-1,3-benzoxazole-6-carboxylate (9h):

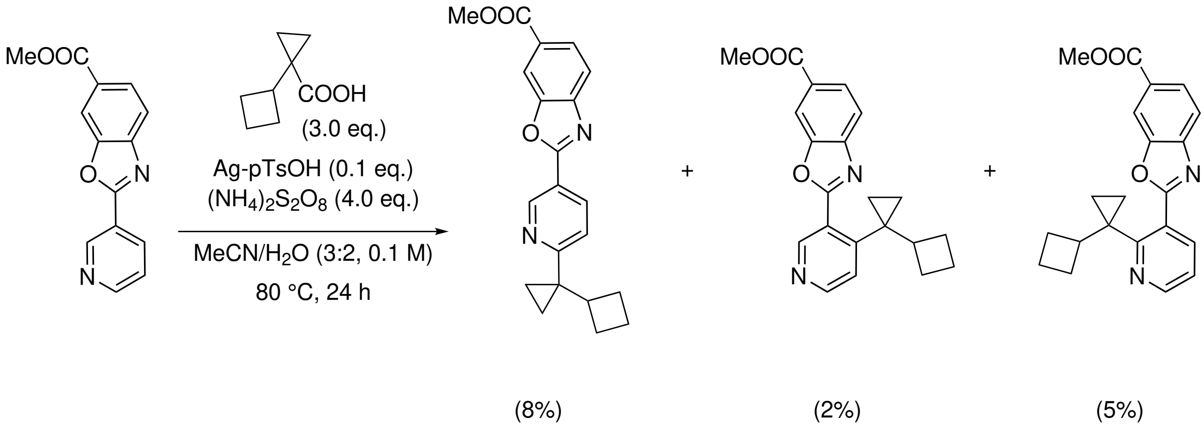

11 **9f** **9g** **9h**

Figure S17: Synthesis of modifiable building blocks (**9f, 9g, 9h**).

A mixture of 2-(3-Pyridyl)-1,3-benzoxazole-6-carboxylic acid methyl ester (**11**, 120 mg, 472 µmol, 1.0 eq), 1-Cyclobutylcyclopropanecarboxylic acid (198.49 mg, 1.42 mmol, 3.0 eq), Ammonium persulfate (323.13 mg, 1.42 mmol, 3.0 eq) and Silver tetrafluoroborate (9.19 mg, 47.2 µmol, 0.1 eq) in 5.7 mL degassed MeCN and 3.8 mL degassed water was stirred at 40 °C for 4 h. Afterwards, the temperature was increased to 50 °C and the mixture was stirred for another 17 h and another 3 h at 60 °C. After 23 h of total reaction time, an additional 1.0 eq of Ammonium persulfate was added to the reaction mixture. The reaction mixture was quenched with sat. $NaHCO_3$ solution and extracted with EtOAc. The combined organic layers were washed with water, and brine, dried over Na_2SO_4, filtered and concentrated to dryness. The crude material was purified by silica gel column chromatography (12 g) using an EtOAc gradient (0%-50%) in heptane. The solvent was evaporated from product containing fractions. Product fractions were combined to give the title compounds 2-[6-(1-Cyclobutylcyclopropyl)-3-pyridyl]-1,3-benzoxazole-6-carboxylic acid methyl ester (**9f**, 19 mg, 54.5 µmol, 8%) as off-white solid, 2-[2-(1-Cyclobutylcyclopropyl)-3-pyridyl]-1,3-benzoxazole-6-carboxylic acid methyl ester (**9g**, 2.9 mg, 8.32 µmol, 2%) as off-white solid, 2-[4-(1-Cyclobutylcyclopropyl)-3-pyridyl]-1,3-benzoxazole-6-carboxylic acid methyl ester (**9h**, 9.0 mg, 25.8 µmol, 5%) as off-white solid.

9f:
^{1}H NMR (600 MHz, $CDCl_3$) δ (ppm) 9.34 (dd, J = 2.3, 0.8 Hz, 1H), 8.39 (dd, J = 8.4, 2.3 Hz, 1H), 8.30 (d, J = 2.2 Hz, 1H), 8.12 (dd, J = 8.3, 1.5 Hz, 1H), 7.80 (dd, J = 8.4, 0.6 Hz, 1H), 7.41 (dd, J = 8.4, 0.8 Hz, 1H), 3.98 (s, 3H), 3.28 (quin, J = 8.5 Hz, 1H), 1.99 – 1.91 (m, 6H), 1.25 – 1.22 (m, 2H), 1.07 – 1.03 (m, 2H). **GC-MS** $C_{21}H_{20}N_2O_3$; calc. for $(M+H^+)$: 349.2, found: 349.1.

9g:
^{1}H NMR (600 MHz, $CDCl_3$) δ (ppm) 8.75 (dd, J = 4.8, 1.8 Hz, 1H), 8.43 – 8.37 (m, 1H), 8.31 (d, J = 2.2 Hz, 1H), 8.15 (dd, J = 8.3, 1.5 Hz, 1H), 7.85 (dd, J = 8.4, 0.6 Hz, 1H), 7.35 (dd, J = 7.8, 4.8 Hz, 1H), 4.00 (s, 3H), 3.08 – 2.98 (m, 1H), 1.92 – 1.85 (m, 2H), 1.84 – 1.70 (m, 3H), 1.67 – 1.62 (m, 1H), 0.99 – 0.89 (m, 2H), 0.84 – 0.76 (m, 2H). **HRMS** $C_{21}H_{20}N_2O_3$; calc. for $(M+H^+)$: 349.1547, found: 349.1551.

9h:
^{1}H NMR (600 MHz, $CDCl_3$) δ (ppm) 9.31 (s, 1H), 8.66 (br d, J = 4.9 Hz, 1H), 8.31 (d, J = 2.2 Hz, 1H), 8.15 (dd, J = 8.3, 1.5 Hz, 1H), 7.87 (dd, J = 8.4, 0.7 Hz, 1H), 7.40 (d, J = 5.1 Hz, 1H), 4.00 (s, 3H), 3.01

– 2.91 (m, 1H), 1.89 – 1.63 (m, 6H), 0.95 – 0.93 (m, 1H), 0.99 – 0.91 (m, 2H), 0.70 – 0.62 (m, 2H). **HRMS** $C_{21}H_{20}N_2O_3$; calc. for $(M+H^+)$: 349.1547, found: 349.1549.

methyl 2-(4,6-Dicyclohexyl-3-pyridyl)-1,3-benzoxazole-6-carboxylate (9i):

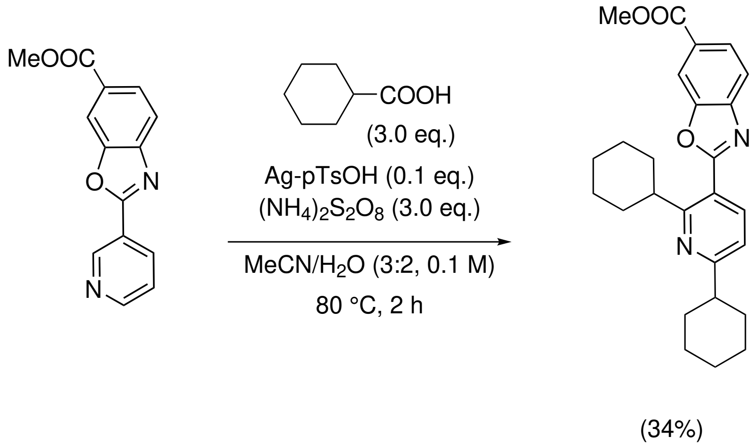

11 **9i**

Figure S18: Synthesis of modifiable building block (**9i**).

A mixture of 2-(3-Pyridyl)-1,3-benzoxazole-6-carboxylic acid methyl ester (**11**, 200 mg, 786.66 µmol, 1.0 eq), Cyclohexanecarboxylic acid (302.48 mg, 293.67 µL, 2.36 mmol, 3.0 eq), Ammonium persulfate (538.55 mg, 2.36 mmol, 3.0 eq) and Silver p-toluenesulfonate (21.95 mg, 78.67 µmol, 0.1 eq) in 9.4 mL degassed MeCN and 6.3 mL degassed water was stirred at 80 °C for 2 h. The reaction mixture was quenched with sat. $NaHCO_3$ solution and extracted with EtOAc. The combined organic layers were washed with water, and brine, dried over Na_2SO_4, filtered and concentrated to dryness. The crude material was purified by silica gel column chromatography (12 g) using an EtOAc gradient (0%-50%) in heptane. The solvent was evaporated from product containing fractions. Product fractions were combined to give the title compound 2-(4,6-Dicyclohexyl-3-pyridyl)-1,3-benzoxazole-6-carboxylic acid methyl ester (**9i**, 117 mg, 280 µmol, 34%) as a light yellow solid.

9i:

^{1}H NMR (600 MHz, DMSO-d$_6$) δ (ppm) 9.10 (s, 1H), 8.35 (d, J = 2.2 Hz, 1H), 8.06 (dd, J = 8.4, 1.6 Hz, 1H), 7.98 (dd, J = 8.3, 0.6 Hz, 1H), 7.48 (s, 1H), 3.92 (s, 3H), 3.68 (tt, J = 11.7, 2.9 Hz, 1H), 2.80 (tt, J = 11.9, 3.4 Hz, 1H), 1.89 – 1.79 (m, 10H), 1.48 – 1.21 (m, 10H). **GC-MS** $C_{26}H_{30}N_2O_3$; calc. for (M+H$^+$): 419.2, found: 419.2.

SI5.3.4 Synthesis of MAGL inhibitor molecules

2-[4-[2-(3-Pyridyl)-1,3-benzoxazole-6-carbonyl]piperazino]-3H-quinazolin-4-one (**17**):

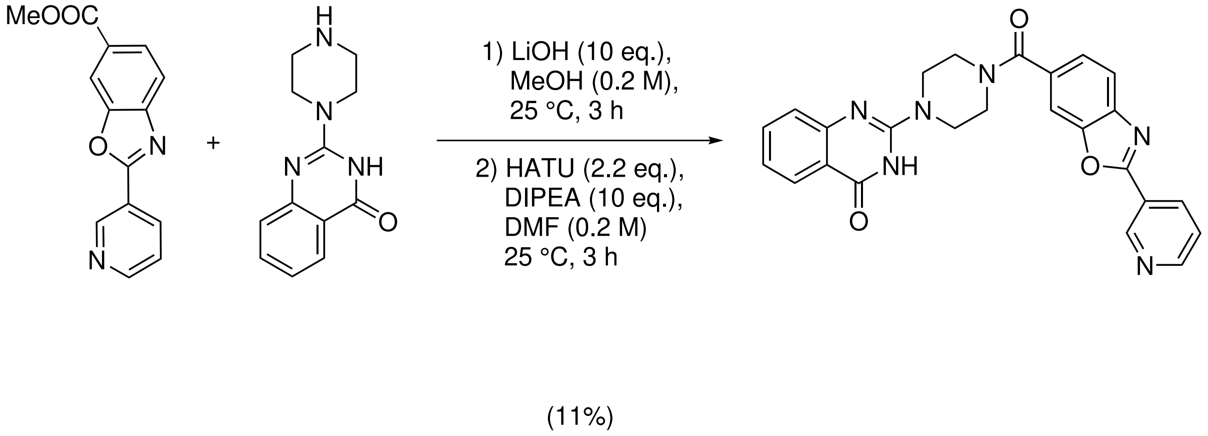

11 **13** **17**

Figure S19: Synthesis of MAGL inhibitor molecule (**17**).

2-(3-Pyridyl)-1,3-benzoxazole-6-carboxylic acid methyl ester (**11**, 100 mg, 393 µmol, 1.0 eq) was diluted in 2.34 mL methanol and 5M LiOH (787 µL, 3.93 mmol, 10.0 eq) was added. The mixture was stirred at rt for 3 h to saponify the ester. Full saponification was seen in LC-MS. Subsequently, the solvent was evaporated, the product washed with toluene, toluene was evaporated and the product redissolved in 1.17 mL DMF. HATU (165 mg, 433 µmol, 1.1 eq) and DIEA (508 mg, 687 µL, 3.93 mmol, 10 eq) were added to activate the acid and the mixture was stirred at 25 °C for 1 h. Then 2-piperazino-3H-quinazolin-4-one (**H1**, 107 mg, 472 µmol, 1.2 eq) in 1.17 mL DMF was added and the mixture was stirred at rt for 5 h. After 3 h additional 1.1 eq of HATU was added, as no amidation was observed in LC-MS. The reaction mixture was quenched with sat. $NaHCO_3$ solution and extracted with EtOAc. The combined organic layers were washed with brine, dried over Na_2SO_4, filtered and concentrated to dryness. The crude was purified by reversed-phase HPLC using a MeCN gradient (20%-98%) in H_2O [(acidic)]. The solvent was removed from product containing fractions. Product fractions were combined to give the title compound 2-[4-[2-(3-Pyridyl)-1,3-benzoxazole-6-carbonyl]piperazino]-3H-quinazolin-4-one (**17**, 20.2 mg, 44.6 µmol, 11%) as yellow solid.

^{1}H NMR (600 MHz, $CDCl_3$) δ (ppm) 11.50 (s, 1H), 9.39 (dd, J = 2.2, 0.7 Hz, 1H), 8.84 (dd, J = 4.8, 1.6 Hz, 1H), 8.57 (ddd, J = 8.0, 2.2, 1.7 Hz, 1H), 7.99 – 7.91 (m, 3H), 7.69 (ddd, J = 8.0, 4.8, 0.9 Hz, 1H), 7.64 – 7.59 (m, 1H), 7.53 (dd, J = 8.1, 1.1 Hz, 1H), 7.30 (br d, J = 8.2 Hz, 1H), 7.17 (t, J = 7.4 Hz, 1H), 3.84 – 3.44 (m, 8H). **HRMS** $C_{25}H_{20}N_6O_3$; calc. for ($M+H^+$): 453.1670, found: 453.1675.

2-[4-[2-(4-Cyclopentyl-3-pyridyl)-1,3-benzoxazole-6-carbonyl]piperazin-1-yl]-3 H-quinazolin-4-one - (18):

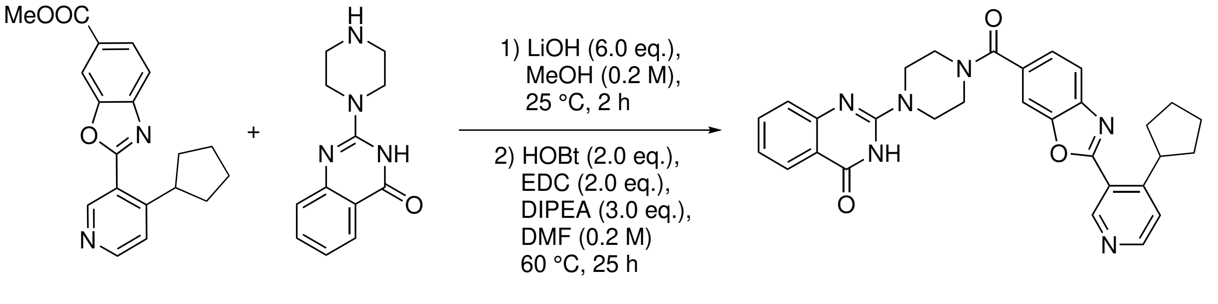

(3%)

9a **13** **18**

Figure S20: Synthesis of MAGL inhibitor molecule (**Inh1**).

2-(4-Cyclopentyl-3-pyridyl)-1,3-benzoxazole-6-carboxylic acid methyl ester (**9a**, 42.1 mg, 131 µmol, 1.0 eq) was diluted in 1200 µL ethanol and 1M LiOH (783.6 µL, 784 µmol, 6.0 eq) was added. The mixture was stirred at rt for 2 h to saponify the ester. Full saponification was seen in LC-MS. Subsequently, the solvent was evaporated, the product washed with toluene, toluene was evaporated and the product redissolved in 600µL DMF. A mixture of 2-Piperazino-3H-quinazolin-4-one;dihydrochloride (**13**, 39.6 mg, 131 µmol, 1.0 eq), 1-(3-Dimethylaminopropyl)-3-ethylcarbodiimide hydrochloride (50.1 mg, 261 µmol, 2.0 eq), DIEA (50.6 mg, 68.4 µL, 392 µmol, 3.0 eq) and 1-Hydroxybenzotriazole (35.3 mg, 261 µmol, 2.0 eq) in 600 µL DMF was stirred at 25 °C for 0.5 h. Then the acid from the first step was added and the mixture was stirred at RT for 19 h. As only little conversion was seen in LC-MS the temperature was raised to 40 °C and the mixture was stirred for another 3 h before an additional raise of temperature to 60 °C for another 3 h. The crude was extracted with TBME using $NaHCO_3$, NH_4Cl and brine solutions. The organic layers were combined and evaporated to dryness. The combined fractions were purified by reversed-phase HPLC using a MeCN gradient (20%-98%) in H_2O [(acidic)]. The solvent was removed from product containing fractions. Product fractions were combined to give the title compound 2-[4-[2-(4-Cyclopentyl-3-pyridyl)-1,3-benzoxazole-6-carbonyl]piperazino]-3H-quinazolin-4-one (**18**, 1.90 mg, 3.65 µmol, 3%) as white solid.

^{1}H NMR (600 MHz, $CDCl_3$) δ (ppm) 10.88 – 10.32 (m, 1H), 9.24 – 9.22 (m, 1H), 8.68 – 8.66 (m, 1H), 8.10 – 8.06 (m, 1H), 7.92 – 7.88 (m, 1H), 7.81 – 7.78 (m, 1H), 7.67 – 7.62 (m, 1H), 7.54 – 7.50 (m, 1H), 7.46 – 7.44 (m, 1H), 7.43 – 7.40 (m, 1H), 7.25 – 7.21 (m, 1H), 4.22 – 4.12 (m, 1H), 3.93 – 3.75 (m, 6H), 2.25 – 2.15 (m, 2H), 1.93 – 1.86 (m, 2H), 1.82 – 1.77 (m, 2H), 1.72 – 1.63 (m, 2H). **^{13}C NMR (151 MHz, $CDCl_3$)** δ (ppm) 156.4, 151.6, 150.7, 150.0, 143.2, 135.0, 132.3, 126.2, 125.3, 123.8, 123.3, 121.5, 120.4, 116.9, 110.1, 45.1, 41.1, 34.1, 25.8. **HRMS** $C_{30}H_{28}N_6O_3$; calc. for $(M+H^+)$: 521.2296, found: 521.2300.

2-[4-[2-(4,6-Dicyclohexyl-3-pyridyl)-1,3-benzoxazole-6-carbonyl]piperazin-1-yl]-3 H-quinazolin-4-one (19):

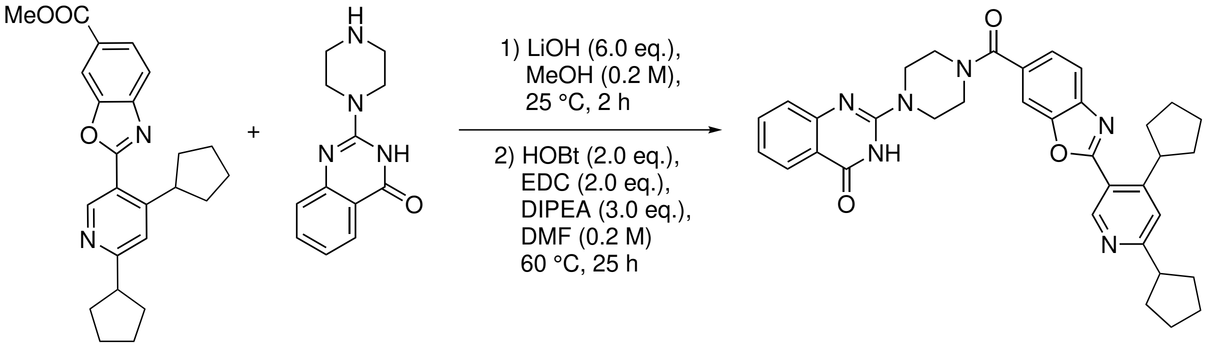

(1%)

9i **13** **19**

Figure S21: Synthesis of MAGL inhibitor molecule (**19**).

2-(4,6-Dicyclohexyl-3-pyridyl)-1,3-benzoxazole-6-carboxylic acid methyl ester (**9i**, 117 mg, 279 µmol, 1.0 eq) was diluted in 1.5 mL ethanol and 1M LiOH (2.51 mL, 2.51 mmol, 9.0 eq) was added. The mixture was stirred at rt for 5 h to saponify the ester. Partial saponification was seen in LC-MS. Subsequently, the solvent was evaporated, the product washed with toluene, toluene was evaporated and the product redissolved in DMF. A mixture of 2-Piperazino-3H-quinazolin-4-one dihydrochloride (**13**, 84.6 mg, 279 µmol, 1.0 eq), 1-(3-Dimethylaminopropyl)-3-ethylcarbodiimide hydrochloride (107 mg, 558 µmol, 2.0 eq), DIEA (108 mg, 146 µL, 837 µmol, 3.0 eq) and 1-hydroxybenzotriazole (75.4 mg, 558 µmol, 2.0 eq) in 100 µL DMF was stirred at 25 °C for 0.5 h. Then the acid from the first step was added and the mixture was stirred at rt for 43 h. The crude was extracted with TBME using $NaHCO_3$, NH_4Cl and brine solutions. The reaction mixture was purified by reversed-phase HPLC using a MeCN gradient (60%-100%) in H_2O [(acidic)]. The solvent was removed from product containing fractions. Product fractions were combined to give the title compound 2-[4-[2-(4,6-Dicyclohexyl-3-pyridyl)-1,3-benzoxazole-6-carbonyl]piperazino]-3H-quinazolin-4-one (**19**, 3.1 mg, 2.20 µmol, 1%) as white solid.

^{1}H NMR (600 MHz, $CDCl_3$) δ (ppm) 11.71 – 10.39 (m, 2H), 9.18 (s, 1H), 8.10 – 8.06 (m, 1H), 7.88 (dd, J = 8.1, 0.6 Hz, 1H), 7.79 (d, J = 2.1 Hz, 1H), 7.65 (ddd, J = 8.4, 7.0, 1.6 Hz, 1H), 7.51 (dd, J = 8.1, 1.5 Hz, 1H), 7.43 (d, J = 8.1 Hz, 1H), 7.27 – 7.26 (m, 1H), 7.26 – 7.22 (m, 1H), 4.01 – 3.75 (m, 10H), 2.86 – 2.74 (m, 1H), 2.03 – 1.94 (m, 5H), 1.94 – 1.88 (m, 5H), 1.71 – 1.39 (m, 31H), 1.39 – 1.29 (m, 6H), 1.27 (s, 7H). **HRMS** $C_{37}H_{40}N_6O_3$; calc. for ($M+H^+$): 617.3234, found: 617.3242.

2-[4-[2-[6-(1-Cyclobutylcyclopropyl)-3-pyridyl]-1,3-benzoxazole-6-carbonyl]piperazin-1-yl]-3 H-quinazolin-4-one (20):

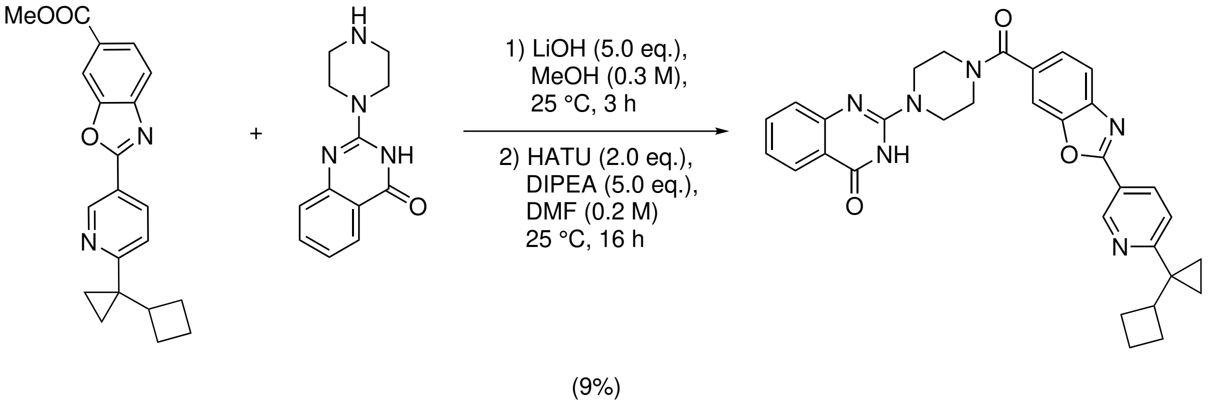

Figure S22: Synthesis of MAGL inhibitor molecule (**20**).

A solution of 2-[6-(1-Cyclobutylcyclopropyl)-3-pyridyl]-1,3-benzoxazole-6-carboxylic acid methyl ester(**9f**, 18.9 mg, 56.5 µmol, 1.0 eq) and 5M LiOH (56.5 µL, 282.6 µmol, 5.0 eq) in 200 µL methanol was stirred for 3 h. The reaction mixture was diluted with toluene and evaporated to dryness. Then HATU (23.64 mg, 62.18 µmol, 1.1 eq), DIEA (36.5 mg, 49.4 µL, 282 µmol, 5.0 eq) and 300 µL of MeCN was stirred at 25 °C for 1 h. Then 2-piperazino-3H-quinazolin-4-one;dihydrochloride (**13**, 17.1 mg, 56.5 µmol, 1.0 eq) was added. The reaction mixture was stirred at 25 °C for 16 h. The reaction mixture was diluted with H_2O and extracted with EtOAc. The combined organic layers were washed with water and brine, dried over Na_2SO_4, filtered and concentrated to dryness. The crude material was purified by reversed-phase HPLC (Gemini NX, 12 nm, 5 µm, 75 x 30 mm) using a MeCN gradient (20%-98%) in H_2O + 0.1% HCOOH. The solvent was removed from product containing fractions. Evaporation of solvents gave the title compound 2-[4-[2-[6-(1-Cyclobutylcyclopropyl)-3-pyridyl]-1,3-benzoxazole-6-carbonyl]piperazino]-3H-quinazolin-4-one (**20**, 3.00 mg, 5.49 µmol 9%) as an off-white powder.

^{1}H NMR (600 MHz, $CDCl_3$) δ (ppm) 9.35 (s, 1H), 8.41 (br d, J = 7.9 Hz, 1H), 8.07 (d, J = 7.9 Hz, 1H), 7.85 (d, J = 8.1 Hz, 1H), 7.76 (s, 1H), 7.67 (br t, J = 7.2 Hz, 1H), 7.50 (br d, J = 8.1 Hz, 1H), 7.45 – 7.41 (m, 1H), 7.27 – 7.22 (m, 2H), 3.89 (br s, 8H), 3.29 (quin, J = 8.5 Hz, 1H), 2.10 – 2.04 (m, 2H), 2.00 – 1.88 (m, 1H), 1.69 (td, J = 9.2, 2.2 Hz, 2H), 1.29 – 1.22 (m, 4H), 1.09 – 1.05 (m, 2H). **^{13}C NMR (151 MHz, $CDCl_3$)** δ (ppm) 170.0, 150.4, 149.5, 143.6, 126.4, 124.2, 120.2, 110.3, 37.4, 29.7, 28.5, 25.4, 17.6, 13.3. **HRMS** $C_{32}H_{30}N_6O_3$; calc. for (M+H$^+$): 547.2452, found: 547.2452.

2-[4-[2-(2-Cyclopentyl-3-pyridyl)-1,3-benzoxazole-6-carbonyl]piperazin-1-yl]-3 H-quinazolin-4-one(21)-
,
2-[4-[2-(6-Cyclopentyl-3-pyridyl)-1,3-benzoxazole-6-carbonyl]piperazin-1-yl]-3 H-quinazolin-4-one -
(**22**):

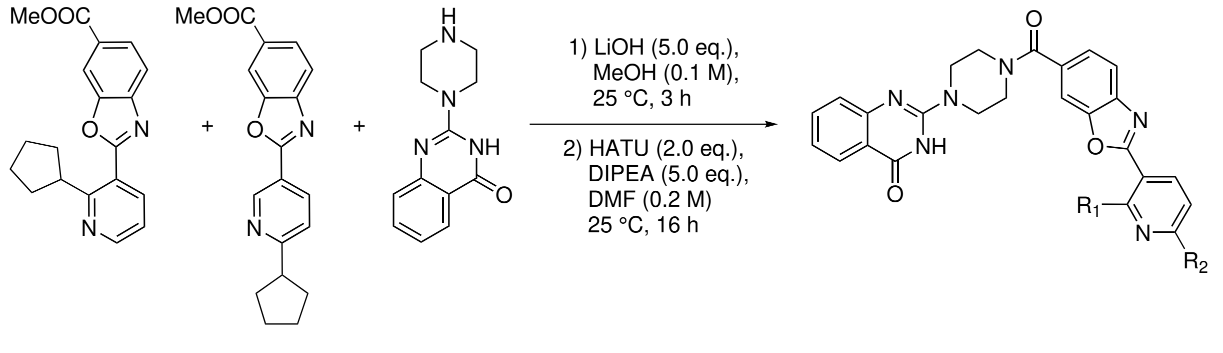

9b **9a** **13**

21 (18%): R_1 = cPen, R_2 = H
22 (29%): R_1 = H, R_2 = cPen

Figure S23: Synthesis of MAGL inhibitor molecules (**21**) and (**22**).

A solution of 2-(2-Cyclopentyl-3-pyridyl)-1,3-benzoxazole-6-carboxylic acid methyl ester (**9b**, 18.5 mg, 57.4 µmol, 1.0 eq), 2-(4-Cyclopentyl-3-pyridyl)-1,3-benzoxazole-6-carboxylic acid methyl ester (**9a**, 18.5 mg, 57.4 µmol, 1.0 eq), 5M LiOH (57.4 µL, 287 µmol, 5.0 eq) in 200 µL methanol was stirred for 3 h. The reaction mixture was diluted with toluene and evaporated to dryness. Then HATU (24.0 mg, 63.1 µmol, 1.1 eq), DIEA (37.1 mg, 50.1 µL, 287 µmol, 5.0 eq) and 300 µL of MeCN were added and the reaction was stirred at 25 °C for 1 h. Then 2-Piperazino-3H-quinazolin-4-one;dihydrochloride (**H1**, 17.1 mg, 56.5 µmol, 1.0 eq) was added. The reaction mixture was stirred at 25 °C for 16 h. The reaction mixture was diluted with H_2O and extracted with EtOAc. The combined organic layers were washed with water, and brine, dried over Na_2SO_4, filtered and concentrated to dryness. The crude material was purified by SFC (OJ-H, 5 µm, 250 x 20 mm) using a MeOH gradient (40%-60%) in supercritical CO_2. Evaporation of solvents gave the title compounds 2-[4-[2-(2-Cyclopentyl-3-pyridyl)-1,3-benzoxazole-6-carbonyl]piperazino]-3H-quinazolin-4-one (**21**, 5.50 mg, 10.6 µmol 18%) as off-white solid and 2-[4-[2-(6-Cyclopentyl-3-pyridyl)-1,3-benzoxazole-6-carbonyl]piperazino]-3H-quinazolin-4-one (**22**, 8.80 mg, 16.9 µmol, 29%) as off-white solid.

21:
^{1}H NMR (600 MHz, CDCl$_3$) δ (ppm) 11.15 – 10.80 (m, 1H), 8.75 (dd, J = 4.7, 1.8 Hz, 1H), 8.34 (dd, J = 7.9, 1.8 Hz, 1H), 8.07 (dd, J = 8.0, 1.3 Hz, 1H), 7.90 (dd, J = 8.1, 0.6 Hz, 1H), 7.81 – 7.76 (m, 1H), 7.65 (ddd, J = 8.4, 7.0, 1.6 Hz, 1H), 7.51 (dd, J = 8.1, 1.5 Hz, 1H), 7.42 (d, J = 7.9 Hz, 1H), 7.29 (dd, J = 7.9, 4.7 Hz, 1H), 7.23 (ddd, J = 7.9, 7.1, 1.1 Hz, 1H), 4.01 – 3.71 (m, 8H), 2.17 – 2.09 (m, 2H), 2.03 – 1.96 (m, 2H), 1.96 – 1.88 (m, 2H), 1.79 – 1.79 (m, 1H), 1.79 – 1.71 (m, 1H). **HRMS** $C_{30}H_{28}N_6O_3$; calc. for $(M+H^+)$: 521.2296, found: 521.2302.**22**:

^{1}H NMR (600 MHz, CDCl$_3$) δ (ppm) 11.09 – 10.73 (m, 1H), 9.41 (dd, J = 2.2, 0.7 Hz, 1H), 8.43 (dd, J = 8.2, 2.3 Hz, 1H), 8.07 (dd, J = 8.0, 1.2 Hz, 1H), 7.86 (dd, J = 8.1, 0.6 Hz, 1H), 7.77 (d, J = 2.1 Hz, 1H), 7.64 (ddd, J = 8.4, 7.0, 1.6 Hz, 1H), 7.50 (dd, J = 8.1, 1.5 Hz, 1H), 7.42 (br d, J = 8.3 Hz, 1H), 7.39 (d, J = 8.0 Hz, 1H), 7.25 – 7.21 (m, 1H), 4.04 – 3.69 (m, 8H), 3.34 – 3.28 (m, 1H), 2.19 – 2.13 (m, 2H), 1.94 – 1.81 (m, 4H), 1.80 – 1.73 (m, 2H). **HRMS** $C_{30}H_{28}N_6O_3$; calc. for $(M+H^+)$: 521.2296, found: 521.2303.

[4-(5-Chlorooxazolo[4,5-b]pyridin-2-yl)piperazin-1-yl]-[2-(4-cyclopentyl-3-pyridyl)-1,3-benzoxazol-6-yl]methanone (23):

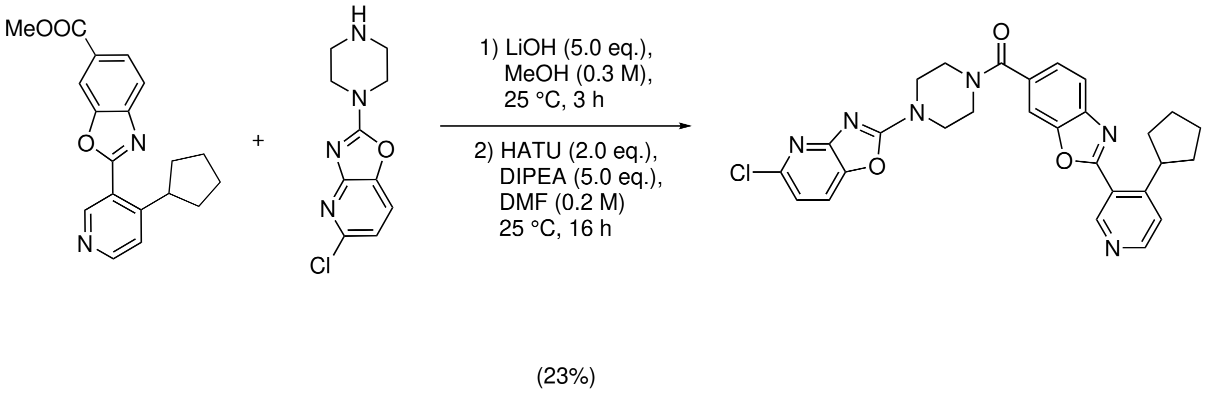

Figure S24: Synthesis of MAGL inhibitor molecule (**23**).

2-(4-Cyclopentyl-3-pyridyl)-1,3-benzoxazole-6-carboxylic acid methyl ester (**9a**, 43.2 mg, 134 µmol, 1.0 eq) was diluted in 500 µL methanol and 5M LiOH (134 µL, 670 µmol, 5.0 eq) was added. The mixture was stirred at rt for 3 h to saponify the ester. Full saponification was seen in LC-MS. Subsequently, the solvent was evaporated, and the product washed with toluene. Toluene was evaporated and the product redissolved in 250 µL DMF. HATU (61.2 mg, 161 µmol, 1.2 eq) was added to activate the acid and the mixture was stirred at 25 °C for 1 h. Then DIEA (86.6 mg, 117 µL, 670 µmol, 5.0 eq) and 5-Chloro-2-piperazino-oxazolo[4,5-b]pyridine hydrochloride (**14**, 44.7 mg, 161 µmol, 1.2 eq) in 250 µL DMF was added and the mixture was stirred at 25 °C for 17 h. After 16 h, an additional 5.0 eq of DIEA and after 20 h an additional 0.8 eq of HATU were added. After 17 h, the temperature was raised to 40 °C and stirred for another 4 h. After 21 h, full amidation was observed. The crude material was purified by silica gel column chromatography (12 g) using an EtOAc gradient (0%-75%) in heptane. The solvent was removed from product containing fractions. Further purification was done by reversed-phase HPLC using a MeCN gradient (0%-98%) in H_2O [(acidic)]. The solvent was removed from product containing fractions to give the title compound [4-(5-Chlorooxazolo[4,5-b]pyridin-2-yl)piperazino]-[2-(4-cyclopentyl-3-pyridyl)-1,3-benzoxazol-6-yl]methanone (**23**, 17.4 mg, 32.9 µmol, 23%) as a white solid.

^{1}H NMR (600 MHz, $CDCl_3$) δ (ppm) 9.23 (br s, 1H), 8.75 – 8.61 (m, 1H), 7.90 (dd, J = 8.1, 0.6 Hz, 1H), 7.78 (d, J = 2.1 Hz, 1H), 7.50 (dd, J = 8.1, 1.5 Hz, 1H), 7.49 – 7.44 (m, 1H), 7.43 (d, J = 8.2 Hz, 1H), 6.99 (d, J = 8.2 Hz, 1H), 4.18 (quin, J = 8.4 Hz, 1H), 4.06 – 3.61 (m, 8H), 2.24 – 2.17 (m, 2H), 1.93 – 1.84 (m, 2H), 1.83 – 1.75 (m, 2H), 1.71 – 1.63 (m, 2H). **HRMS** $C_{28}H_{25}ClN_6O_3$; calc. for $(M+H^+)$: 529.1750, found: 529.1744.

2-(2-Cyclopentyl-3-pyridyl)-1,3-benzoxazole-6-carboxylic acid methyl ester (**9b**, 15.4 mg, 47.8 µmol, 1.0 eq) was diluted in 500 µL methanol and 5M LiOH (47.8 µL, 239 µmol, 5.0 eq) was added. The mixture was stirred at rt for 5 h to saponify the ester. After 2 h additional 5.0 eq of LiOH were added. Full saponification was seen in LC-MS. Subsequently, the solvent was evaporated and the product was washed with toluene. Toluene was evaporated and the product redissolved in 250 DMF. HATU (36.3 mg, 95.6 µmol, 2.0 eq) was added to activate the acid and the mixture was stirred at 25 °C for 1 h. Then DIEA (61.7 mg, 83.4 µL, 478 µmol, 10.0 eq) and 5-chloro-2-piperazino-oxazolo[4,5-b]pyridine hydrochloride (**14**, 15.8 mg, 57.3 µmol, 1.2 eq) in 250 µL DMF was added and the mixture was stirred at rt for 2 h. After 1 h, an additional 2.0 eq of HATU was added, After 2 h full amidation was observed and the reaction mixture was quenched with sat. $NaHCO_3$ solution and extracted with EtOAc. The combined organic layers were washed with brine, dried over Na_2SO_4, filtered and concentrated to dryness. Further purification was done by reversed-phase HPLC using a MeCN gradient (20%-98%) in H_2O [(acidic)]. The solvent was removed from product containing fractions to give the title compound [4-(5-Chlorooxazolo[4,5-b]pyridin-2-yl)piperazino]-[2-(2-cyclopentyl-3-pyridyl)-1,3-benzoxazol-6-yl]methanone (**24**, 10.4 mg, 19.7 µmol 41%) as a white solid.

^{1}H NMR (600 MHz, CDCl$_3$) δ (ppm) 8.75 (dd, J = 4.7, 1.8 Hz, 1H), 8.33 (dd, J = 7.9, 1.8 Hz, 1H), 7.89 (dd, J = 8.1, 0.6 Hz, 1H), 7.76 (d, J = 1.5 Hz, 1H), 7.49 (dd, J = 8.1, 1.5 Hz, 1H), 7.43 (d, J = 8.2 Hz, 1H), 7.31 – 7.27 (m, 1H), 6.99 (d, J = 8.2 Hz, 1H), 4.30 (quin, J = 8.3 Hz, 1H), 3.89 – 3.81 (m, 5H), 2.16 – 2.09 (m, 2H), 2.03 – 1.94 (m, 2H), 1.95 – 1.89 (m, 2H), 1.79 – 1.71 (m, 2H). **HRMS** $C_{28}H_{25}ClN_6O_3$; calc. for (M+H$^+$): 529.1750, found: 529.1745.

[4-(5-Chlorooxazolo[4,5-b]pyridin-2-yl)piperazin-1-yl]-[2-[4-(4-piperidyl)-3-pyridyl]-1,3-benzoxazol-6-yl]methanone formic acid (25):

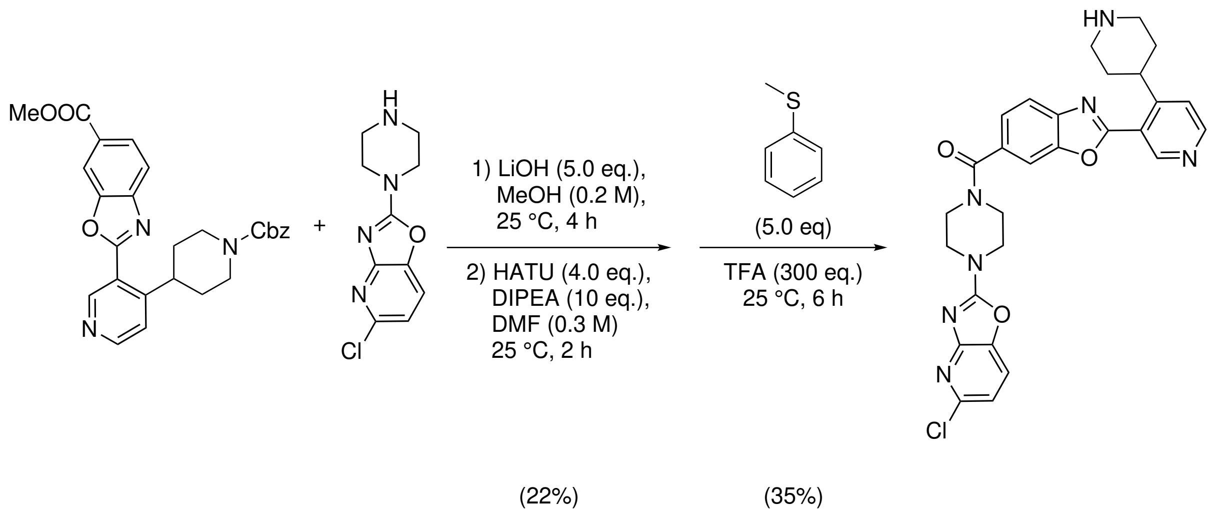

Figure S25: Synthesis of MAGL inhibitor molecule (**25**).

2-[4-(1-Carbobenzoxy-4-piperidyl)-3-pyridyl]-1,3-benzoxazole-6-carboxylic acid methyl ester (**9d**, 39.4 mg, 83.6 µmol, 1.0 eq) was diluted in 500 µL methanol and 5M LiOH (83.6 µL, 418 µmol, 5.0 eq) was added. The mixture was stirred at rt for 3 h to saponify the ester. After 2 h, an additional 5.0 eq of LiOH were added. After 3 h, the temperature was raised to 40 °C. Upon full saponification, the solvent was evaporated, the product was washed with toluene, and the product was redissolved in 250 µL DMF. DIPEA (108 mg, 146 µL, 836 µmol, 10 eq) and HATU (63.6 mg, 167 µmol, 2.0 eq) was added to activate the acid and the mixture was stirred at 25 °C for 1 h. Then 5-Chloro-2-piperazino-oxazolo[4,5-b]pyridine hydrochloride (**14**, 27.6 mg, 100 µmol, 1.2 eq) in 250 µL DMF was added and the mixture was stirred at rt for 2 h. After 1 h, an additional 2.0 eq of HATU was added. The reaction mixture was precipitated with water and the solid was filtered off and purified by reversed-phase HPLC using a MeCN gradient (40%-70%) in H_2O [(acidic)]. The solvent was removed from the product containing fractions to give 4-[3-[6-[4-(5-Chlorooxazolo[4,5-b]pyridin-2-yl)piperazine-1-carbonyl]-1,3-benzoxazol-2-yl]-4-pyridyl]piperidine-1-carboxylic acid benzyl ester (12.8 mg, 18.3 µmol, 22%) as a white solid. 4-[3-[6-[4-(5-Chlorooxazolo[4,5-b]pyridin-2-yl)piperazine-1-carbonyl]-1,3-benzoxazol-2-yl]-4-pyridyl]piperidine-1-carboxylic acid benzyl ester (12.8 mg, 18.3 µmol, 1.0 eq) and Thioanisole (11.4 mg, 10.8 µL, 91.5 µmol, 5.0 eq) were dissolved in TFA (626 mg, 423 µL, 5.49 mmol, 300 eq) and stirred at 25 °C for 6 h. The reaction mixture was quenched with sat. $NaHCO_3$ and extracted with EtOAc. The combined organic layers were washed with water, and brine, dried over Na_2SO_4, filtered and concentrated to dryness. The crude material was purified by reversed-phase HPLC using a MeCN gradient (10%-50%) in H_2O [acidic]. The solvent was removed from product containing fractions to give the title compound, [4-(5-Chlorooxazolo[4,5-b]pyridin-2-yl)piperazino]-[2-[4-(4-piperidyl)-3-pyridyl]-1,3-benzoxazol-6-yl]methanone (**25**, 4 mg, 7.35 µmol, 35%) as a white solid.

^{1}H NMR (600 MHz, DMSO-d$_6$) δ (ppm) 9.17 (s, 1H), 8.74 (d, J = 5.3 Hz, 1H), 7.99 (d, J = 1.4 Hz, 1H), 7.97 (d, J = 8.1 Hz, 1H), 7.82 (d, J = 8.2 Hz, 1H), 7.59 (d, J = 5.3 Hz, 1H), 7.55 (dd, J = 8.1, 1.5 Hz, 1H), 7.08 (d, J = 8.2 Hz, 1H), 3.89 – 3.70 (m, 9H), 3.18 – 3.14 (m, 3H), 2.79 – 2.71 (m, 2H), 1.84 (br d, J = 12.4 Hz, 2H), 1.78 – 1.67 (m, 2H). **HRMS** $C_{28}H_{26}ClN_7O_3$; calc. for (M+H$^+$): 544.1859, found: 544.1862.

[4-(5-Chlorooxazolo[4,5-b]pyridin-2-yl)piperazin-1-yl]-[2-[2-(4-piperidyl)-3-pyridyl]-1,3-benzoxazol-6-yl]methanone;formic acid (26):

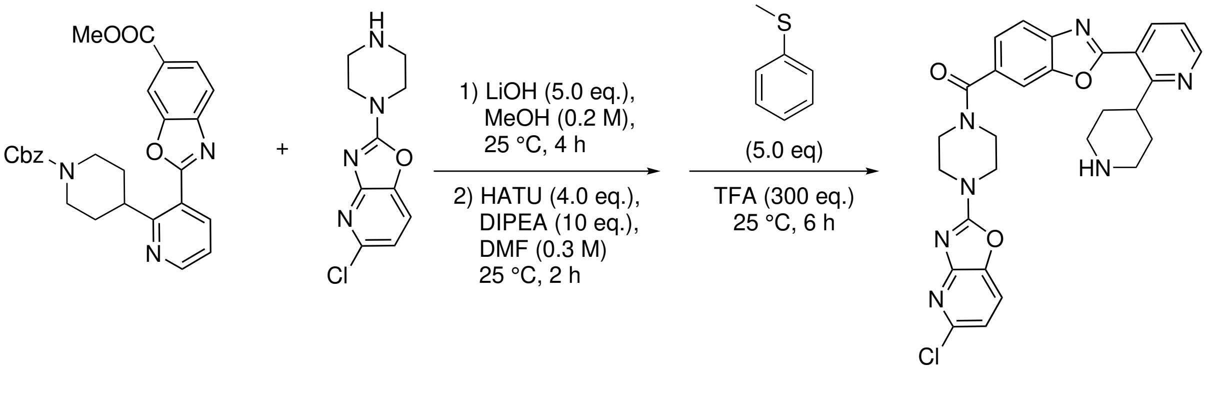

(34%) (39%)

9e **14** **26**

Figure S26: Synthesis of MAGL inhibitor molecule (**26**).

2-[2-(1-Carbobenzoxy-4-piperidyl)-3-pyridyl]-1,3-benzoxazole-6-carboxylic acid methyl ester (**9e**, 26.5 mg, 56.2 µmol, 1.0 eq) was diluted in 500 µL methanol and 5M LiOH (56.2 µL, 281 µmol, 5.0 eq) was added. The mixture was stirred at 25 °C for 4 h to saponify the ester. After 1 h, temperature was raised to 40 °C. Subsequently, the solvent was evaporated, the product washed with toluene, toluene was evaporated and the product redissolved in 250 µL DMF. HATU (23.5 mg, 61.8 µmol, 1.1 eq) and DIEA (72.6 mg, 98.2 µL, 562 µmol, 10 eq) was added to activate the acid and the mixture was stirred at 25 °C for 1 h. Then 5-chloro-2-piperazino-oxazolo[4,5-b]pyridine hydrochloride (**14**, 18.6 mg, 67.4 µmol, 1.2 eq) in 250 µL DMF was added and the mixture was stirred at rt for 2 h. The reaction mixture was precipitated with water and the solid product was removed by filtration. The solid was further purified by reversed-phase HPLC using a MeCN gradient (20%-98%) in H_2O [(acidic)]. The solvent was removed from product containing fractions to give 4-[3-[6-[4-(5-Chlorooxazolo-[4,5-b]pyridin-2-yl)piperazine-1-carbonyl]-1,3-benzoxazol-2-yl]-2-pyridyl]piperidine-1-carboxylic acid benzyl ester (13.4 mg, 19.4 µmol, 34%) as white solid. 4-[3-[6-[4-(5-chlorooxazolo[4,5-b]pyridin-2-yl)piperazine-1-carbonyl]-1,3-benzoxazol-2-yl]-2-pyridyl]piperidine-1-carboxylic acid benzyl ester (13.4 mg, 19.4 µmol, 1.0 eq) and thioanisole (12.0 mg, 11.5 µL, 96.8 µmol, 5.0 eq) were dissolved in TFA (662 mg, 448 µL, 5.81 mmol, 300 eq) and stirred at rt for 6 h. Full deprotection was observed in LC-MS. The reaction mixture was quenched with sat. $NaHCO_3$ and extracted with EtOAc. The combined organic layers were washed with water, and brine, dried over Na_2SO_4, filtered and concentrated to dryness. The crude material was purified by reversed-phase HPLC using a MeCN gradient (10%-60%) in H_2O [acidic]. The solvent was removed from product containing fractions. Evaporation of solvents gave the title compound [4-(5-Chlorooxazolo[4,5-b]pyridin-2-yl)piperazino]-[2-[2-(4-piperidyl)-3-pyridyl]-1,3-benzoxazol-6-yl]methanone 1:1 formic acid (**26**, 4.6 mg, 8.64 µmol, 39%) as white solid.

^{1}H NMR (600 MHz, $CDCl_3$) δ (ppm) 8.76 (dd, J = 4.7, 1.8 Hz, 1H), 8.74 – 8.49 (m, 1H), 8.47 (dd, J = 8.0, 1.8 Hz, 1H), 7.91 – 7.87 (m, 1H), 7.76 (d, J = 2.1 Hz, 1H), 7.51 (dd, J = 8.1, 1.5 Hz, 1H), 7.43 (d, J = 8.2 Hz, 1H), 7.38 (dd, J = 8.0, 4.7 Hz, 1H), 6.99 (d, J = 8.2 Hz, 1H), 4.38 (tt, J = 10.9, 3.4 Hz, 1H), 3.86 (br dd, J = 1.6, 0.9 Hz, 9H), 3.59 (br d, J = 12.2 Hz, 2H), 3.16 (br t, J = 11.5 Hz, 2H), 2.41 – 2.31 (m, 2H), 2.18 (s, 1H). **HRMS** $C_{28}H_{26}ClN_7O_3$; calc. for $(M+H^+)$: 544.1859, found: 544.1865.

[4-(5-Chlorooxazolo[4,5-b]pyridin-2-yl)piperazin-1-yl]-[2-[4-(1-cyclobutylcyclopropyl)-3-pyridyl]-1,3-benzoxazol-6-yl]methanone (27):

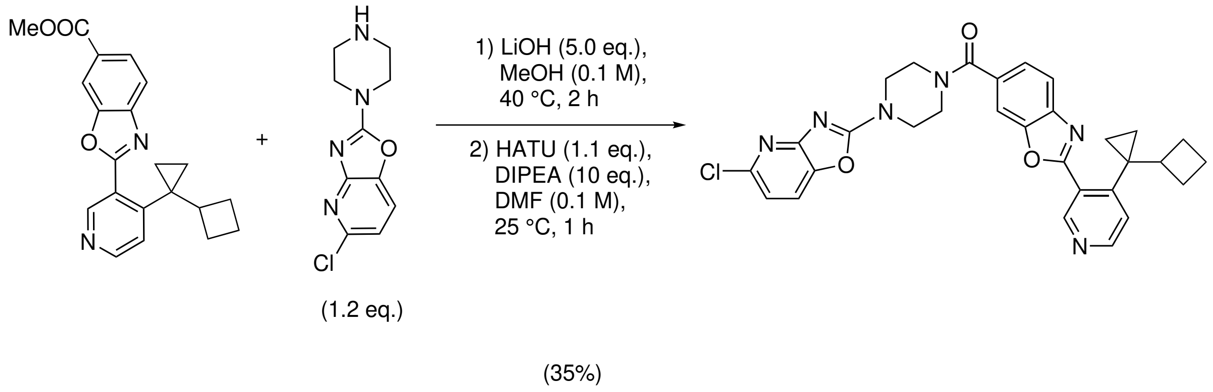

9h **14** **27**

Figure S27: Synthesis of MAGL inhibitor molecule (**27**).

2-[4-(1-Cyclobutylcyclopropyl)-3-pyridyl]-1,3-benzoxazole-6-carboxylic acid methyl ester (**9h**, 24.0 mg, 68.9 µmol, 1.0 eq) was diluted in 500 µL methanol and 5M LiOH (68.9 µL, 344 µmol, 5.0 eq) was added. The mixture was stirred at rt for 2 h to saponify the ester. After 1 h temperature was raised to 40 °C. Full saponification was seen in LC-MS. Subsequently, the solvent was evaporated, the product washed with toluene, toluene was evaporated and the product redissolved in 250 µL DMF. 5-chloro-2-piperazino-oxazolo[4,5-b]pyridine;hydrochloride (**14**, 22.7 mg, 82.7 µmol, 1.2 eq), DIPEA (89.0 mg, 120 µL, 689 µmol, 10 eq) was added and HATU (28.8 mg, 75.8 µmol, 1.1 eq) in 250 µL DMF was added and the mixture was stirred at rt for 1 h. The reaction mixture was precipitated with water and the solid product was removed by filtration. The solid was further purified by reversed-phase HPLC using a MeCN gradient (20%-98%) in H_2O [(acidic)]. The solvent was removed from product containing fractions. Product fractions were combined to give the title compound [4-(5-chlorooxazolo[4,5-b]pyridin-2-yl)piperazino]-[2-[4-(1-Cyclobutylcyclopropyl)-3-pyridyl]-1,3-benzoxazol-6-yl]methanone (**27**, 13.7 mg, 24.7 µmol, 35%) as white solid.

^{1}H NMR (600 MHz, $CDCl_3$) δ (ppm) 9.29 (br s, 1H), 8.66 (br s, 1H), 7.90 (dd, J = 8.1, 0.6 Hz, 1H), 7.77 (d, J = 2.1 Hz, 1H), 7.49 (dd, J = 8.1, 1.5 Hz, 1H), 7.41 (br dd, J = 3.9, 1.5 Hz, 1H), 6.99 (d, J = 8.2 Hz, 1H), 3.86 (br s, 9H), 3.01 – 2.92 (m, 1H), 1.88 – 1.81 (m, 2H), 1.79 – 1.60 (m, 5H), 0.97 – 0.92 (m, 2H), 0.68 – 0.63 (m, 2H). **HRMS** $C_{30}H_{27}ClN_6O_3$; calc. for $(M+H^+)$: 555.1906, found: 555.1911.

[4-(5-Chlorooxazolo[4,5-b]pyridin-2-yl)piperazin-1-yl]-[2-[2-(1-cyclobutylcyclopropyl)-3-pyridyl]-1,3-benzoxazol-6-yl]methanone (28):

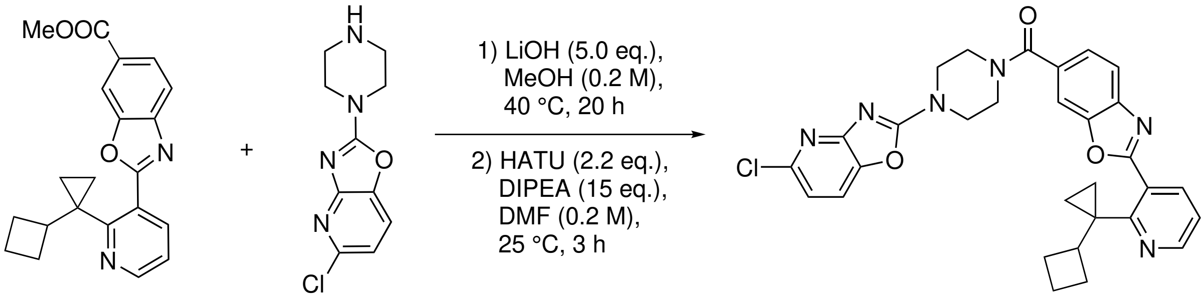

(1.2 eq.)

(14%)

9g **14** **28**

Figure S28: Synthesis of MAGL inhibitor molecule (**28**).

2-[2-(1-Cyclobutylcyclopropyl)-3-pyridyl]-1,3-benzoxazole-6-carboxylic acid methyl ester (**9g**, 6.1 mg, 17.5 µmol, 1.0 eq) was diluted in 500 µL methanol and 5M LiOH (17.5 µL, 87.5 µmol, 5.0 eq) was added. The mixture was stirred at rt for 20 h to saponify the ester. After 1 h temperature was raised to 40 °C. Full saponification was seen in LC-MS. Subsequently, the solvent was evaporated, the product washed with toluene, toluene was evaporated and the product redissolved in 250 µL DMF. HATU (7.32 mg, 19.2 µmol, 1.1 eq) and DIEA (22.6 mg, 30.6 µL, 175 µmol, 10 eq) was added to activate the acid and the mixture was stirred at 25 °C for 1 h. Then 5-chloro-2-piperazino-oxazolo[4,5-b]pyridine;hydrochloride (**14**, 5.78 mg, 21.0 µmol, 1.2 eq) in 250 µL DMF was added and the mixture was stirred at rt for 3 h. After 1 h additional 5.0 eq of DIPEA and after 2 h additional 1.1 eq of HATU were added, as no amidation was observed in LC-MS. The reaction mixture was quenched with sat. $NaHCO_3$ solution and extracted with EtOAc. The combined organic layers were washed with brine, dried over Na_2SO_4, filtered and concentrated to dryness. The reaction mixture was purified by reversed-phase HPLC using a MeCN gradient (40%-70%) in H_2O [(acidic)]. The solvent was removed from product containing fractions. Product fractions were combined to give the title compound [4-(5-chlorooxazolo[4,5-b]pyridin-2-yl)piperazino]-[2-[2-(1-cyclobutylcyclopropyl)-3-pyridyl]-1,3-benzoxazol-6-yl]methanone (**28**, 1.4 mg, 2.52 µmol, 14%) as white solid.

^{1}H NMR (600 MHz, $CDCl_3$) δ (ppm) 8.76 (dd, J = 4.8, 1.7 Hz, 1H), 8.44 – 8.30 (m, 1H), 7.91 – 7.87 (m, 1H), 7.78 (d, J = 2.1 Hz, 1H), 7.49 (dd, J = 8.1, 1.5 Hz, 1H), 7.43 (d, J = 8.2 Hz, 1H), 7.39 – 7.31 (m, 1H), 7.00 (d, J = 8.2 Hz, 1H), 4.40 – 3.31 (m, 8H), 3.08 – 3.00 (m, 1H), 1.91 – 1.84 (m, 2H), 1.84 – 1.69 (m, 3H), 1.68 – 1.60 (m, 1H), 0.94 (s, 2H), 0.80 – 0.75 (m, 2H). **HRMS** $C_{30}H_{27}ClN_6O_3$; calc. for $(M+H^+)$: 555.1906, found: 555.1926.

[2-(4-Cyclopentyl-3-pyridyl)-1,3-benzoxazol-6-yl]-[6-(3-cyclopropyl-1,2,4-triazol-1-yl)-2-azaspiro[3.3]-heptan-2-yl]methanone (**29**):

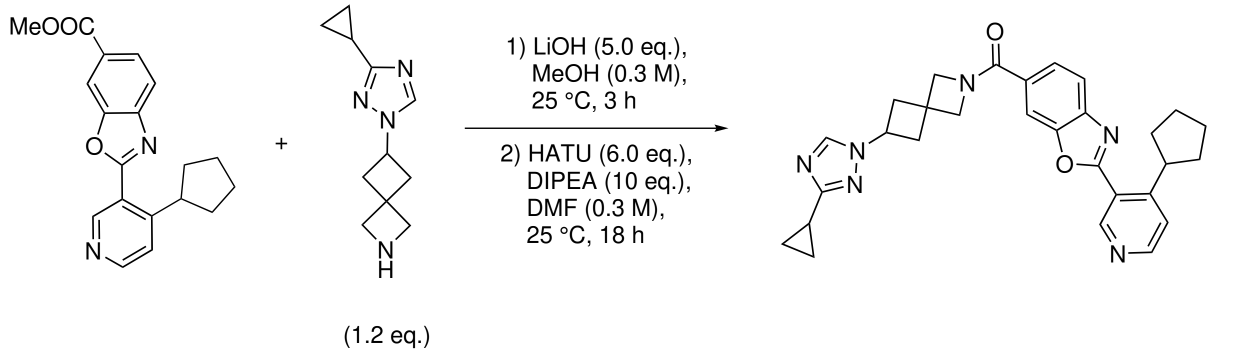

9a **15** **29**

Figure S29: Synthesis of MAGL inhibitor molecule (**29**).

2-(4-Cyclopentyl-3-pyridyl)-1,3-benzoxazole-6-carboxylic acid methyl ester (**9a**, 30.1 mg, 93.4 µmol, 1.0 eq) was diluted in 500 µL methanol and 5M LiOH (93.4 µL, 467 µmol, 5.0 eq) was added. The mixture was stirred at rt for 3 h to saponify the ester. Full saponification was seen in LC-MS. Subsequently, the solvent was evaporated, the product washed with toluene, toluene was evaporated and the product redissolved in 180 µL DMF. HATU (71.0 mg, 187 µmol, 2.0 eq) and DIEA (121 mg, 163 µL, 934 µmol, 10 eq) was added to activate the acid and the mixture was stirred at 25 °C for 1 h. Then 6-(3-cyclopropyl-1,2,4-triazol-1-yl)-2-azaspiro[3.3]heptane;2,2,2-trifluoroacetic acid (**15**, 35.7 mg, 112 µmol, 1.2 eq) in 200 µL DMF was added and the mixture was stirred at rt for 18 h. After 2 h and 17 h additional 2.0 eq of HATU were added, as only small amidation was observed in LC-MS. After 18 h full amidation was observed. The reaction mixture was quenched with sat. $NaHCO_3$ solution and extracted with EtOAc . The combined organic layers were washed with brine, dried over Na_2SO_4, filtered and concentrated to dryness. Further purification was done by reversed-phase HPLC using a MeCN gradient (20%-98%) in H_2O [(acidic)]. The solvent was removed from product containing fractions. Product fractions were combined to give the title compound [2-(4-cyclopentyl-3-pyridyl)-1,3-benzoxazol-6-yl]-[6-(3-cyclopropyl-1,2,4-triazol-1-yl)-2-azaspiro[3.3]heptan-2-yl]methanone (**29**, 8.50 mg, 17.2 µmol, 18%) as white solid.

^{1}H NMR (600 MHz, $CDCl_3$) δ (ppm) 9.23 (s, 1H), 8.67 (br d, J = 5.3 Hz, 1H), 7.96 (d, J = 1.5 Hz, 1H), 7.88 (s, 1H), 7.85 (dd, J = 8.3, 0.6 Hz, 1H), 7.71 (dd, J = 8.3, 1.5 Hz, 1H), 7.45 (d, J = 5.3 Hz, 1H), 4.83 – 4.56 (m, 1H), 4.50 – 4.42 (m, 2H), 4.36 (s, 2H), 4.18 (t, J = 8.4 Hz, 1H), 2.88 – 2.80 (m, 3H), 2.22 – 2.16 (m, 2H), 2.10 – 1.97 (m, 1H), 1.92 – 1.84 (m, 2H), 1.83 – 1.75 (m, 2H), 1.70 – 1.63 (m, 4H), 0.96 (s, 4H). **HRMS** $C_{29}H_{30}N_6O_2$; calc. for $(M+H^+)$: 495.2503, found: 495.2497.

[2-(6-Cyclopentyl-3-pyridyl)-1,3-benzoxazol-6-yl]-[6-(3-cyclopropyl-1,2,4-triazol-1-yl)-2-azaspiro[3.3]-heptan-2-yl]methanone (**30**):

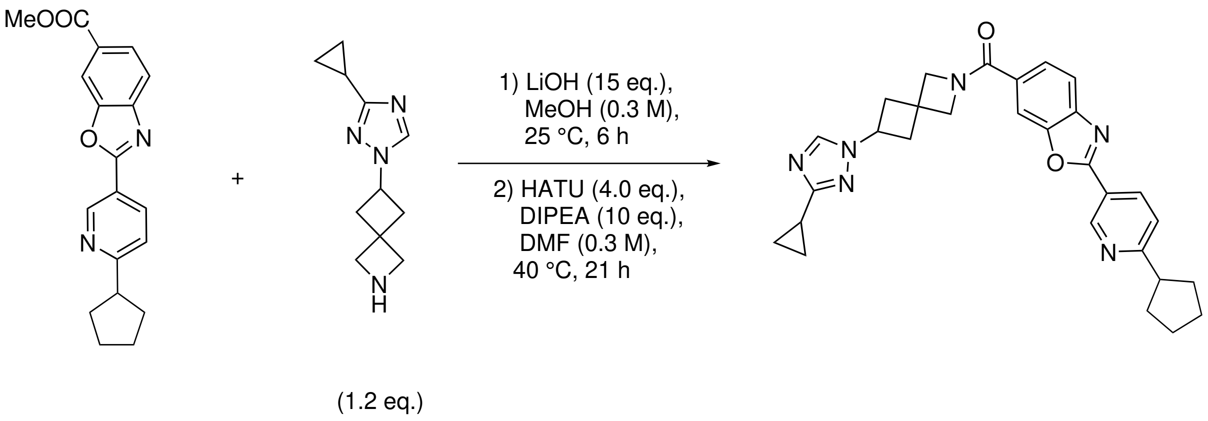

Figure S30: Synthesis of MAGL inhibitor molecule (**30**).

2-(6-cyclopentyl-3-pyridyl)-1,3-benzoxazole-6-carboxylic acid methyl ester (**9c**, 13.8 mg, 42.8 µmol, 1.0 eq) was diluted in 500 µL methanol and 5M LiOH (42.8 µL, 214 µmol, 5.0 eq) was added. The mixture was stirred at rt for 6 h to saponify the ester. After 2 h and 5 h additional 5.0 eq of LiOH were added. Full saponification was seen in LC-MS. Subsequently, the solvent was evaporated, the prodcut washed with toluene, toluene was evaporated and the product redissolved in 250 µL DMF. HATU (32.6 mg, 85.6 µmol, 2.0 eq) was added to activate the acid and the mixture was stirred at 25 °C for 1 h. Then DIEA (55.3 mg, 74.8 µL, 428 µmol, 10 eq) and 6-(3-cyclopropyl-1,2,4-triazol-1-yl)-2-azaspiro[3.3]heptane;2,2,2-trifluoroacetic acid (**15**, 16.4 mg, 51.4 µmol, 1.2 eq) in 250 µL DMF was added and the mixture was stirred at rt for 17 h. After 1 h additional 2.0 eq of HATU was added, as only small amidation was observed in LC-MS. After 17 h the temperature was raised to 40 °C and stirred for another 4 h. After 21 h full amidation was observed. The reaction mixture was quenched with sat. $NaHCO_3$ solution and extracted with EtOAc. The combined organic layers were washed with brine, dried over Na_2SO_4, filtered and concentrated to dryness. Further purification was done by reversed-phase HPLC using a MeCN gradient (0%-98%) in H_2O [(acidic)]. The solvent was removed from product containing fractions. Product fractions were combined to give the title compound [2-(6-Cyclopentyl-3-pyridyl)-1,3-benzoxazol-6-yl]-[6-(3-cyclopropyl-1,2,4-triazol-1-yl)-2-azaspiro[3.3]heptan-2-yl]methanone (**30**, 3.70 mg, 7.48 µmol, 16%) as white solid.

^{1}H NMR (600 MHz, CDCl$_3$) δ (ppm) 9.40 (dd, J = 2.2, 0.7 Hz, 1H), 8.43 (dd, J = 8.2, 2.3 Hz, 1H), 7.94 (d, J = 2.1 Hz, 1H), 7.87 (s, 1H), 7.80 (dd, J = 8.2, 0.6 Hz, 1H), 7.69 (dd, J = 8.3, 1.5 Hz, 1H), 7.38 (d, J = 7.8 Hz, 1H), 4.76 – 4.57 (m, 1H), 4.52 – 4.28 (m, 4H), 3.30 (t, J = 8.3 Hz, 1H), 2.95 – 2.72 (m, 4H), 2.21 – 2.13 (m, 2H), 2.12 – 2.02 (m, 1H), 1.94 – 1.73 (m, 9H), 0.97 (br d, J = 2.9 Hz, 5H). **HRMS** $C_{29}H_{30}N_6O_2$; calc. for (M+H$^+$): 495.2503, found: 495.2495.

[2-(4-Cyclopentyl-3-pyridyl)-1,3-benzoxazol-6-yl]-[3-[4-(thiazole-2-carbonyl)piperazin-1-yl]azetidin-1-yl]methanone (31):

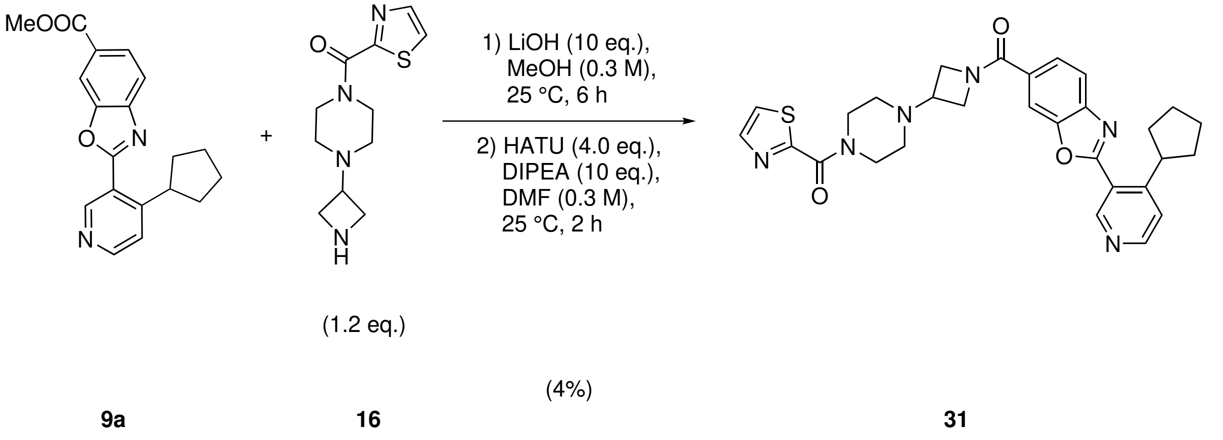

Figure S31: Synthesis of MAGL inhibitor molecule (**31**).

2-(4-Cyclopentyl-3-pyridyl)-1,3-benzoxazole-6-carboxylic acid methyl ester (**9a**, 30.0 mg, 93.1 µmol, 1.0 eq) was diluted in 500 µL methanol and 5M LiOH (93.1 µL, 465 µmol, 5.0 eq) was added. The mixture was stirred at rt for 6 h to saponify the ester. After 5 h additional 5.0 eq of LiOH were added. Full saponification was seen in LC-MS. Subsequently, the solvent was evaporated, the prodcut washed with toluene, toluene was evaporated and the product redissolved in 250 µL DMF. HATU (70.8 mg, 186 µmol, 2.0 eq) was added to activate the acid and the mixture was stirred at 25 °C for 1 h. Then DIPEA (120 mg, 163 µL, 931 µmol, 10 eq) and [4-(azetidin-3-yl)piperazino]-thiazol-2-yl-methanone (**16**, 28.2 mg, 112 µmol, 1.2 eq) in 250 µL DMF was added and the mixture was stirred at rt for 2 h. After 1 h additional 2.0 eq of HATU was added, as only small amidation was observed in LC-MS. After 2 h full amidation was observed. The reaction mixture was quenched with sat. $NaHCO_3$ solution and extracted with EtOAc. The combined organic layers were washed with brine, dried over Na_2SO_4, filtered and concentrated to dryness. Further purification was done by reversed-phase HPLC using a MeCN gradient (20%-98%) in H_2O [(acidic)]. The solvent was removed from product containing fractions. Product fractions were combined to give the title compound [2-(4-Cyclopentyl-3-pyridyl)-1,3-benzoxazol-6-yl]-[3-[4-(thiazole-2-carbonyl)piperazino]azetidin-1-yl]methanone (**31**, 2.00 mg, 3.69 µmol, 4%) as a white solid.

^{1}H NMR (600 MHz, $CDCl_3$) δ (ppm) 9.23 (s, 1H), 8.67 (d, J = 5.3 Hz, 1H), 7.98 – 7.95 (m, 1H), 7.89 (d, J = 3.2 Hz, 1H), 7.85 (d, J = 8.3 Hz, 1H), 7.71 (dd, J = 8.3, 1.5 Hz, 1H), 7.55 (d, J = 3.2 Hz, 1H), 7.46 – 7.42 (m, 1H), 4.66 – 4.22 (m, 5H), 4.21 – 4.12 (m, 2H), 4.04 – 3.79 (m, 2H), 3.34 – 3.27 (m, 1H), 2.61 – 2.42 (m, 4H), 2.24 – 2.16 (m, 2H), 1.92 – 1.84 (m, 2H), 1.79 (br dd, J = 7.4, 4.6 Hz, 2H), 1.70 – 1.63 (m, 3H). **HRMS** $C_{29}H_{30}N_6O_3S$; calc. for $(M+H^+)$: 543.2173, found: 543.2168.

SI6 NMR spectra

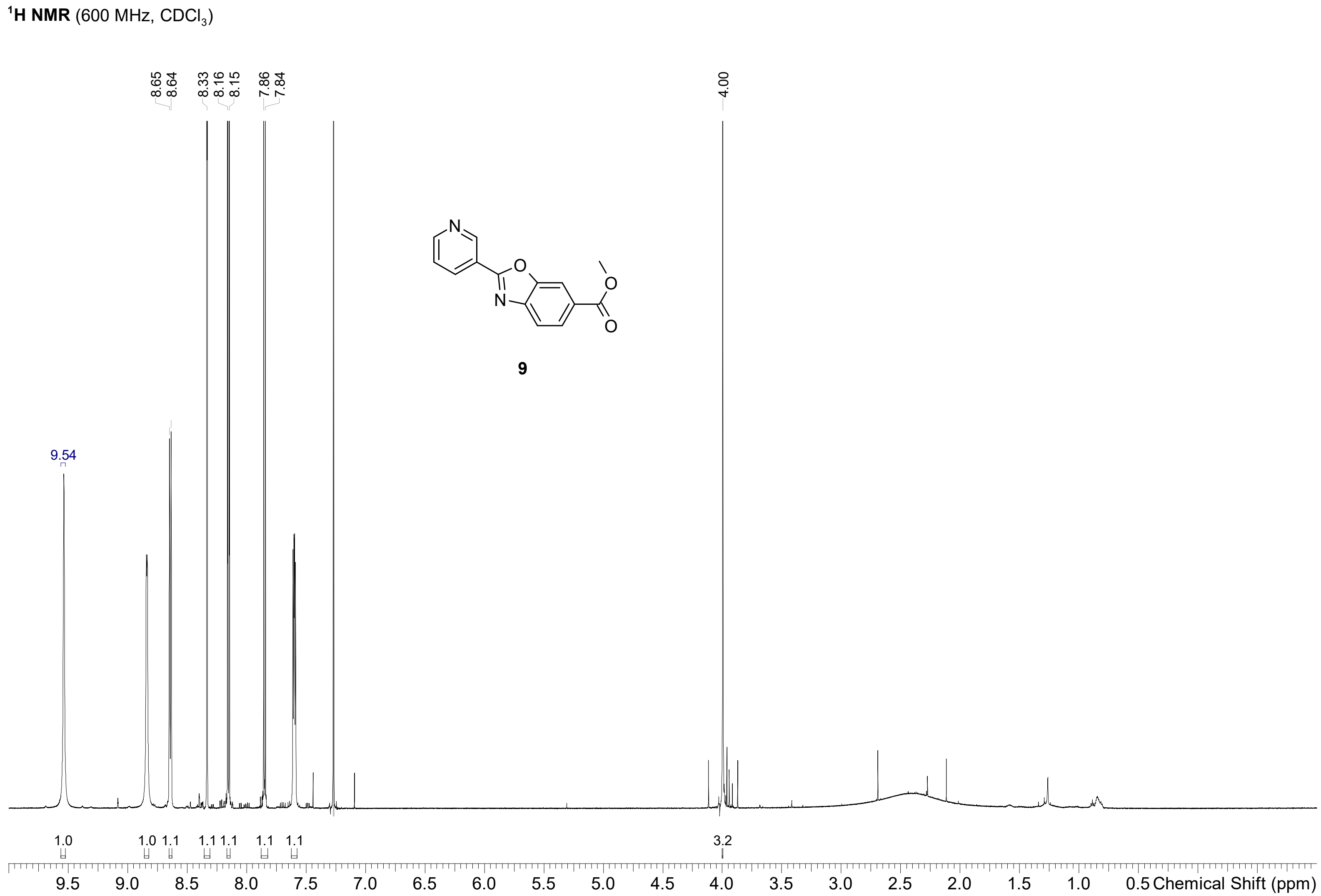

Figure S32: **9**, ^{1}H-NMR spectra.

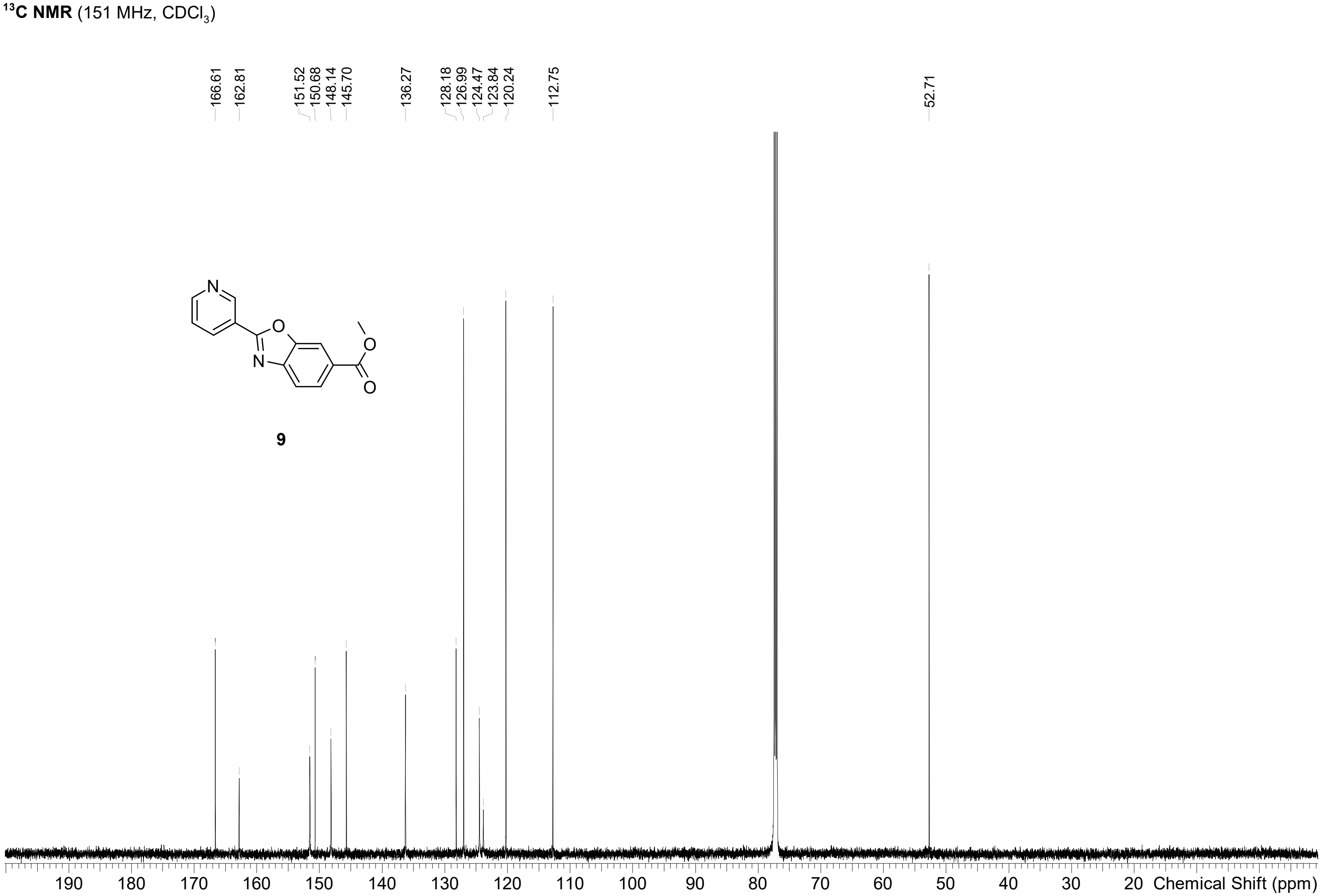

Figure S33: **9**, ^{13}C-NMR spectra.

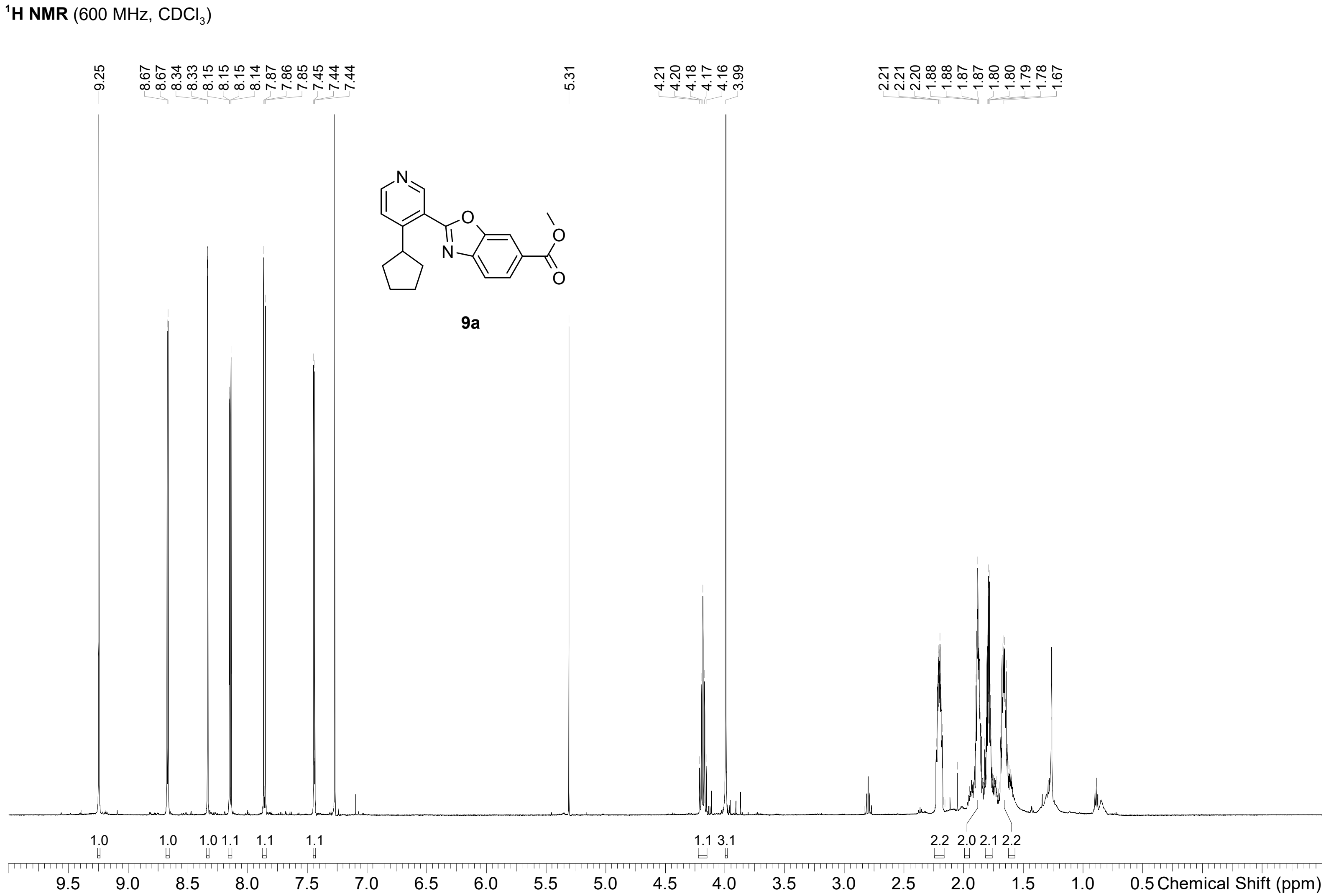

Figure S34: **9a**, ^{1}H-NMR spectra.

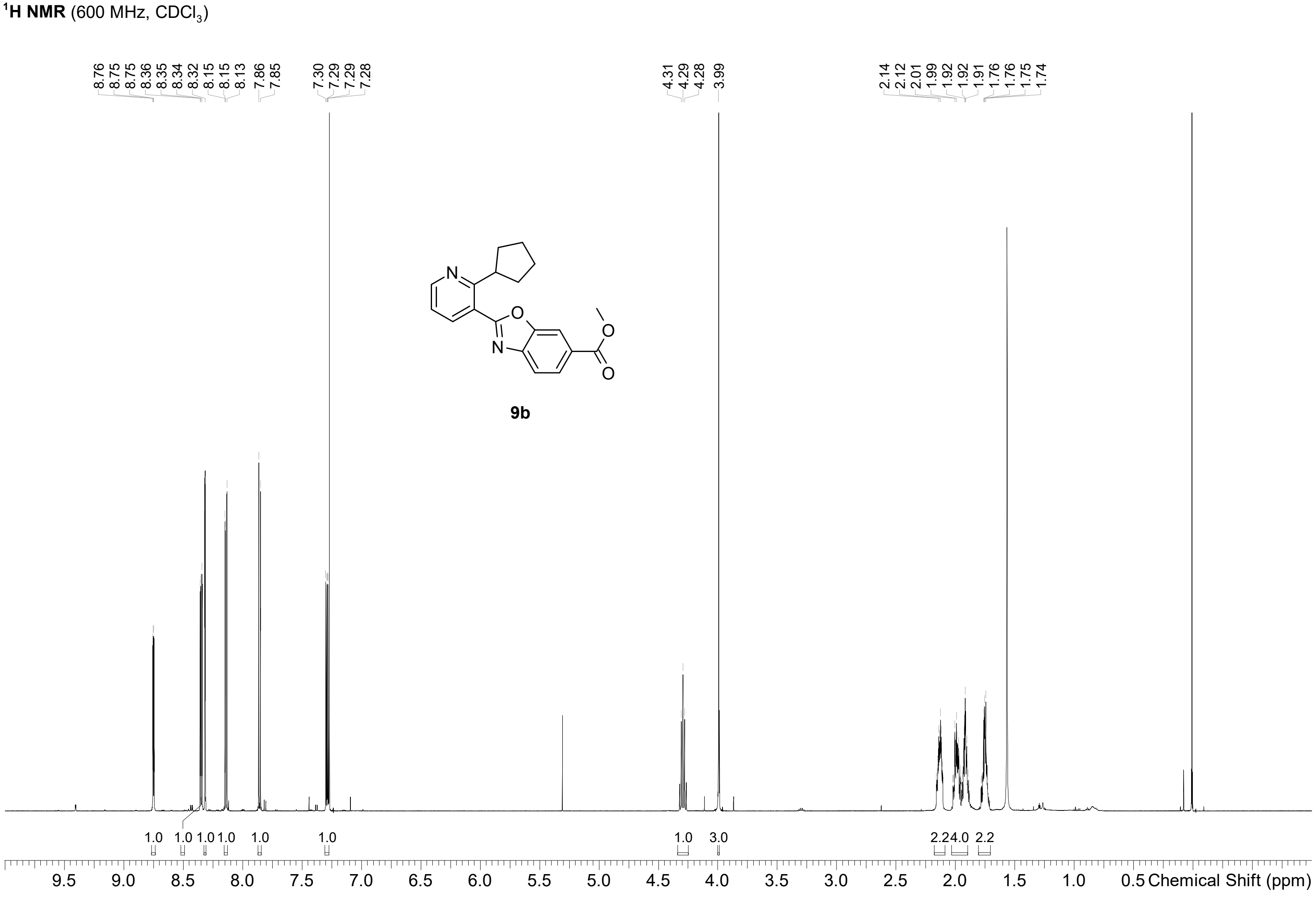

Figure S35: **9b**, ^{1}H-NMR spectra.

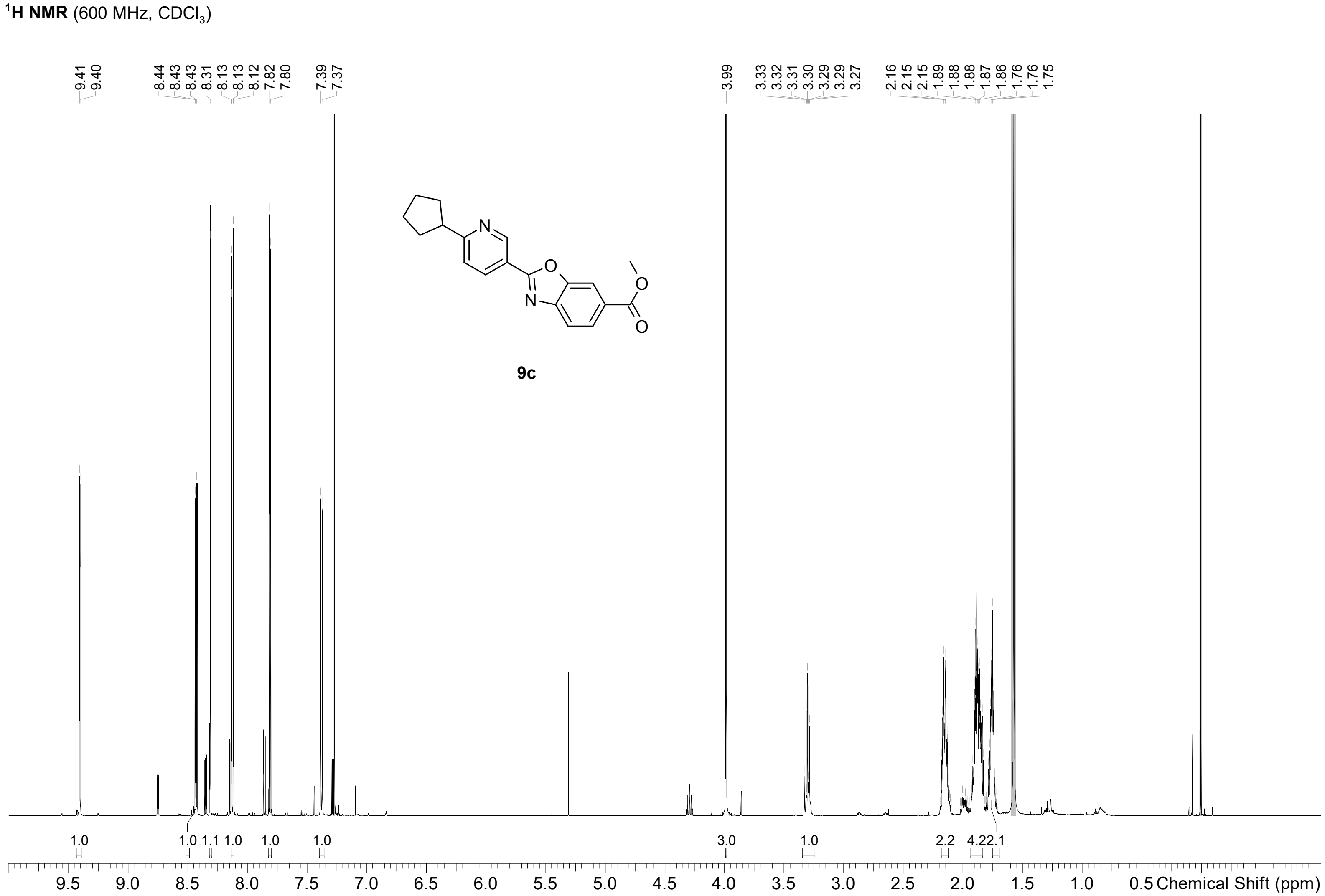

Figure S36: **9c**, ^{1}H-NMR spectra.

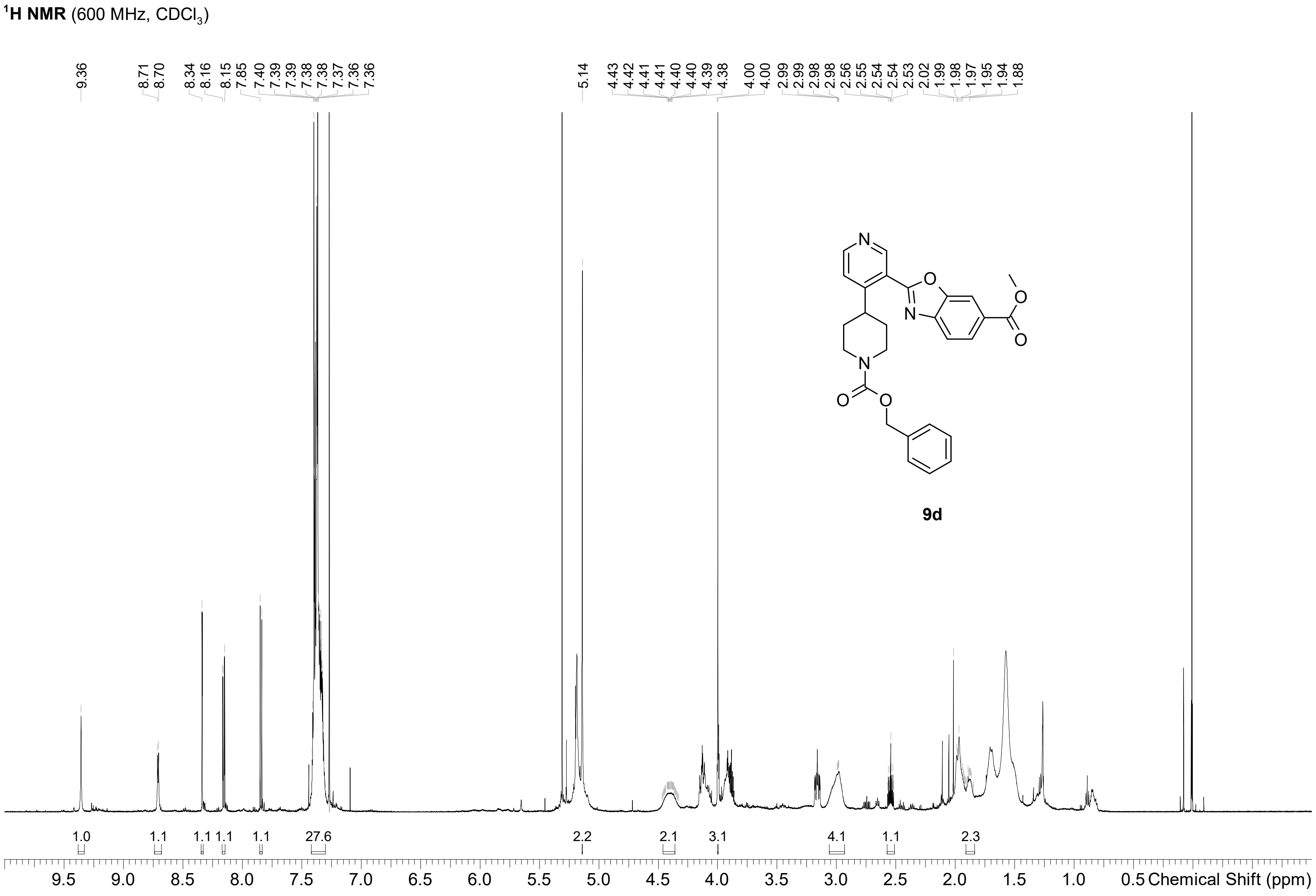

Figure S37: **9d**, ¹H-NMR spectra.

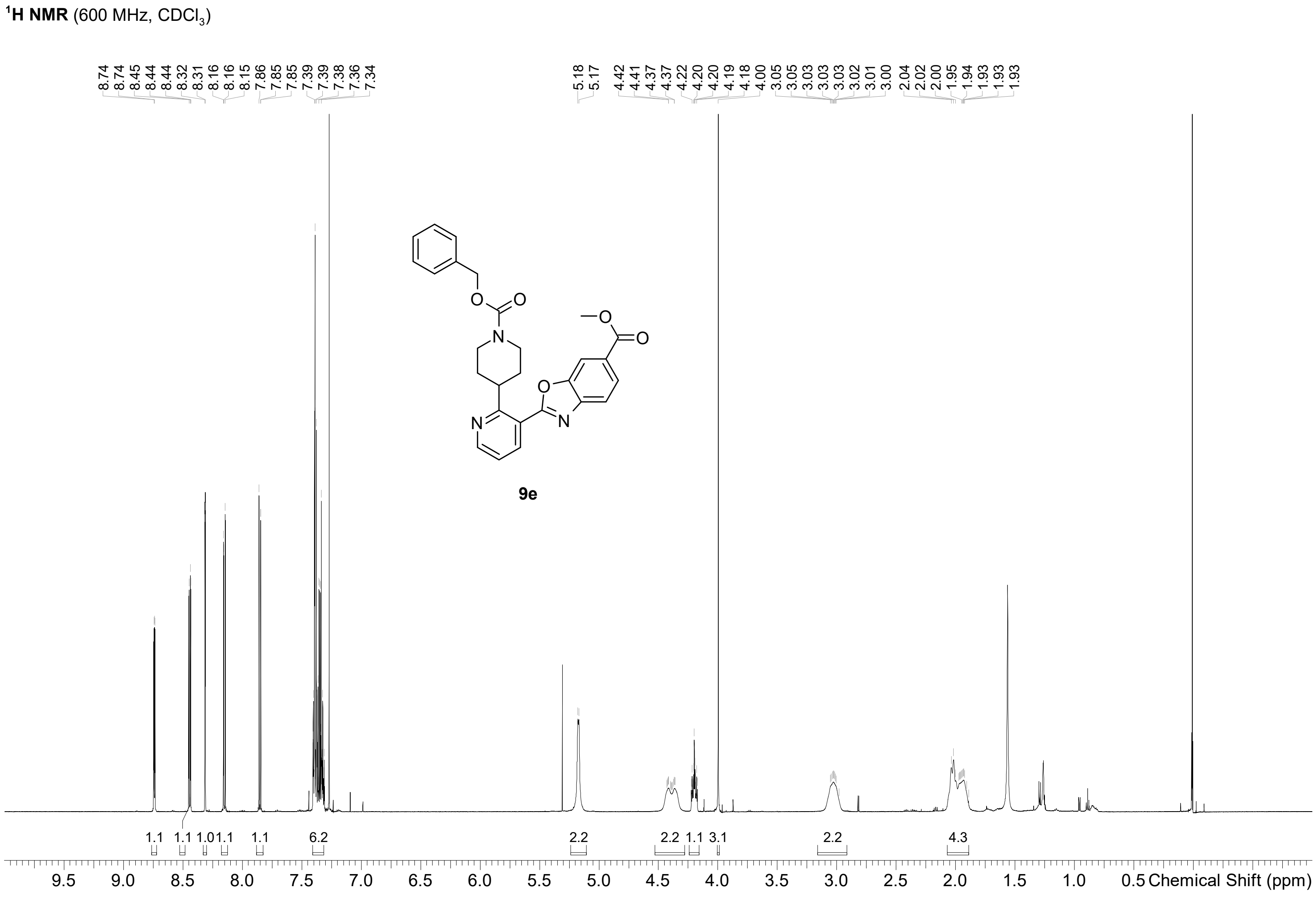

Figure S38: **9e**, ^{1}H-NMR spectra.

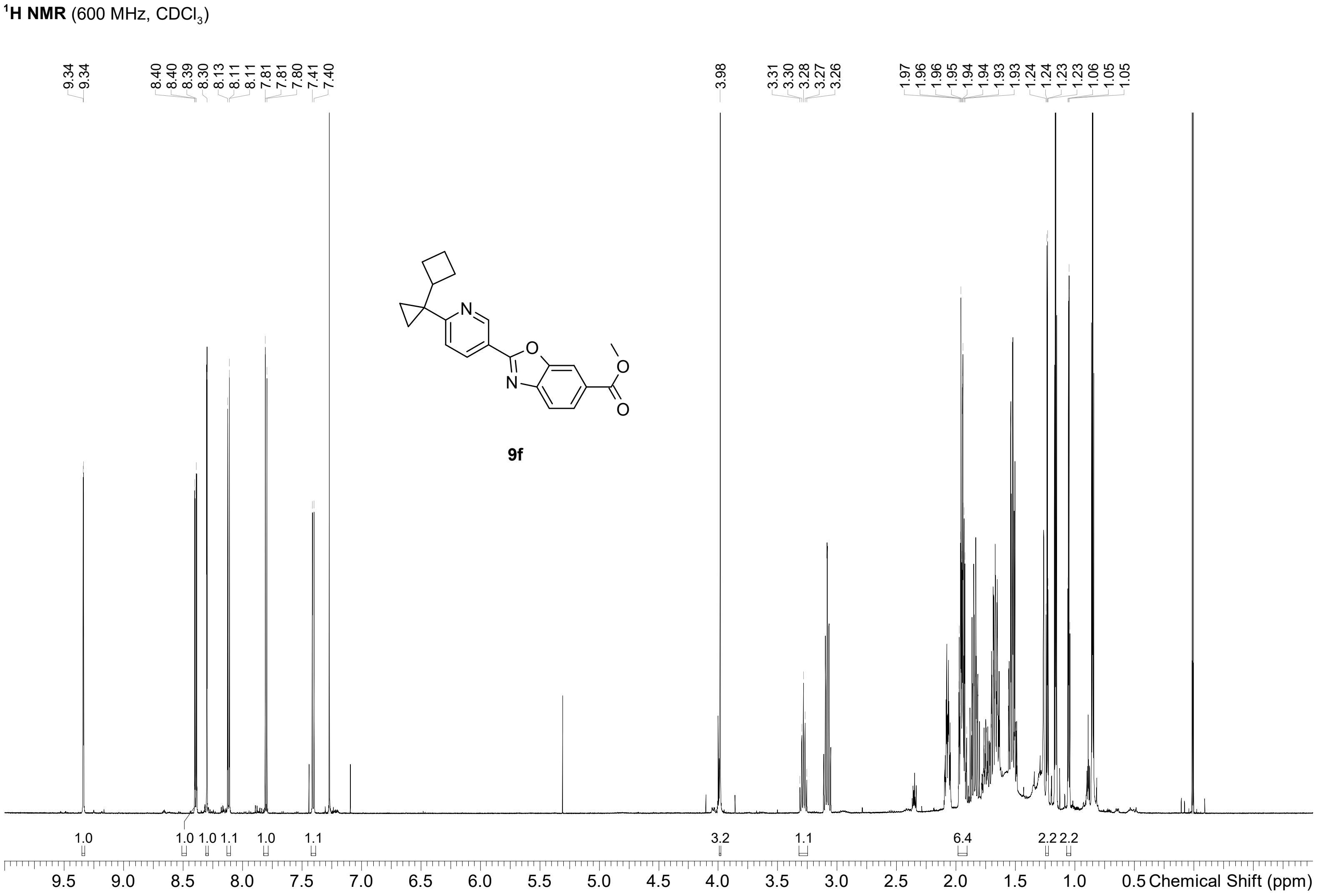

Figure S39: **9f**, ^{1}H-NMR spectra.

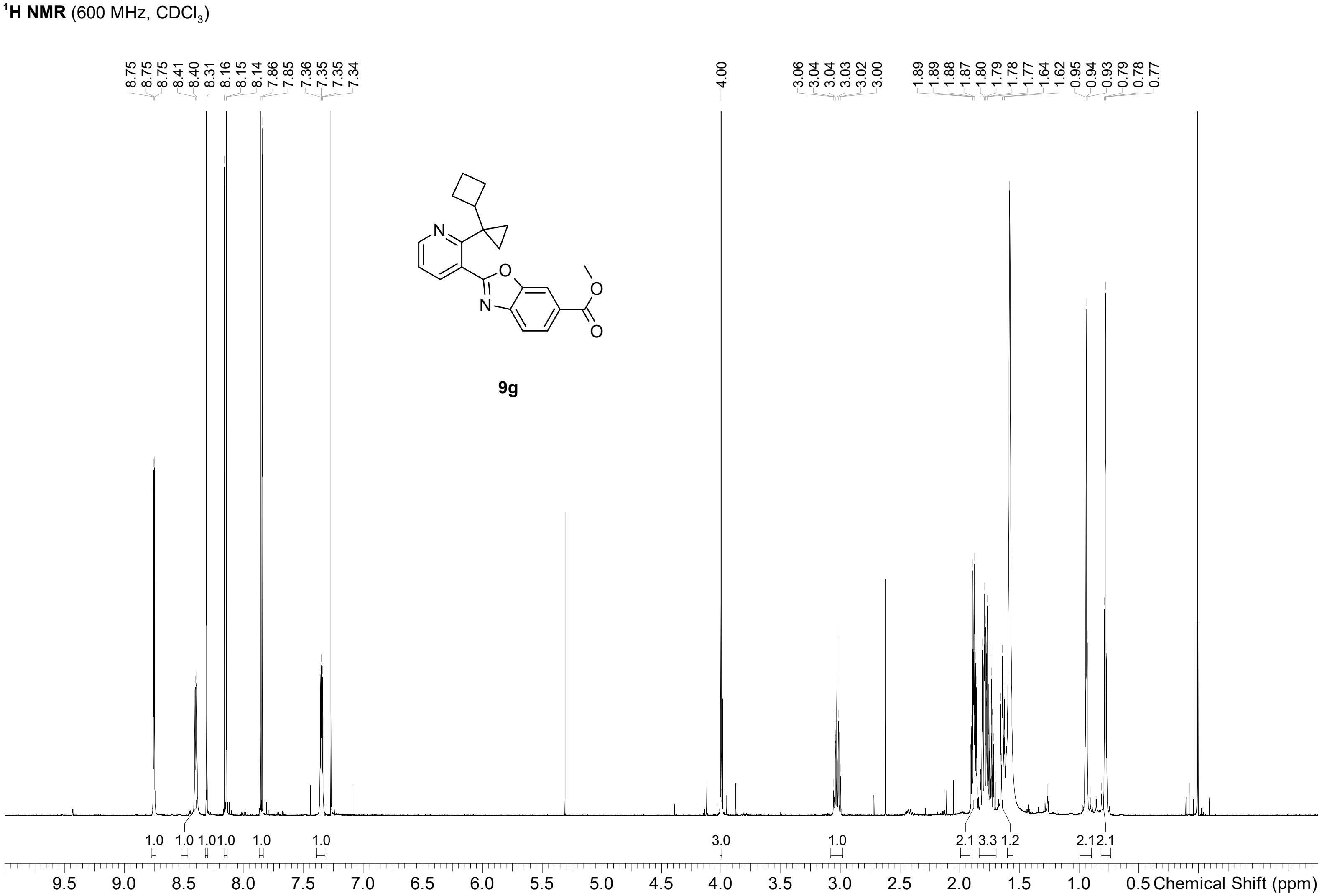

Figure S40: **9g**, ^{1}H-NMR spectra.

^{1}H NMR (600 MHz, $CDCl_3$)

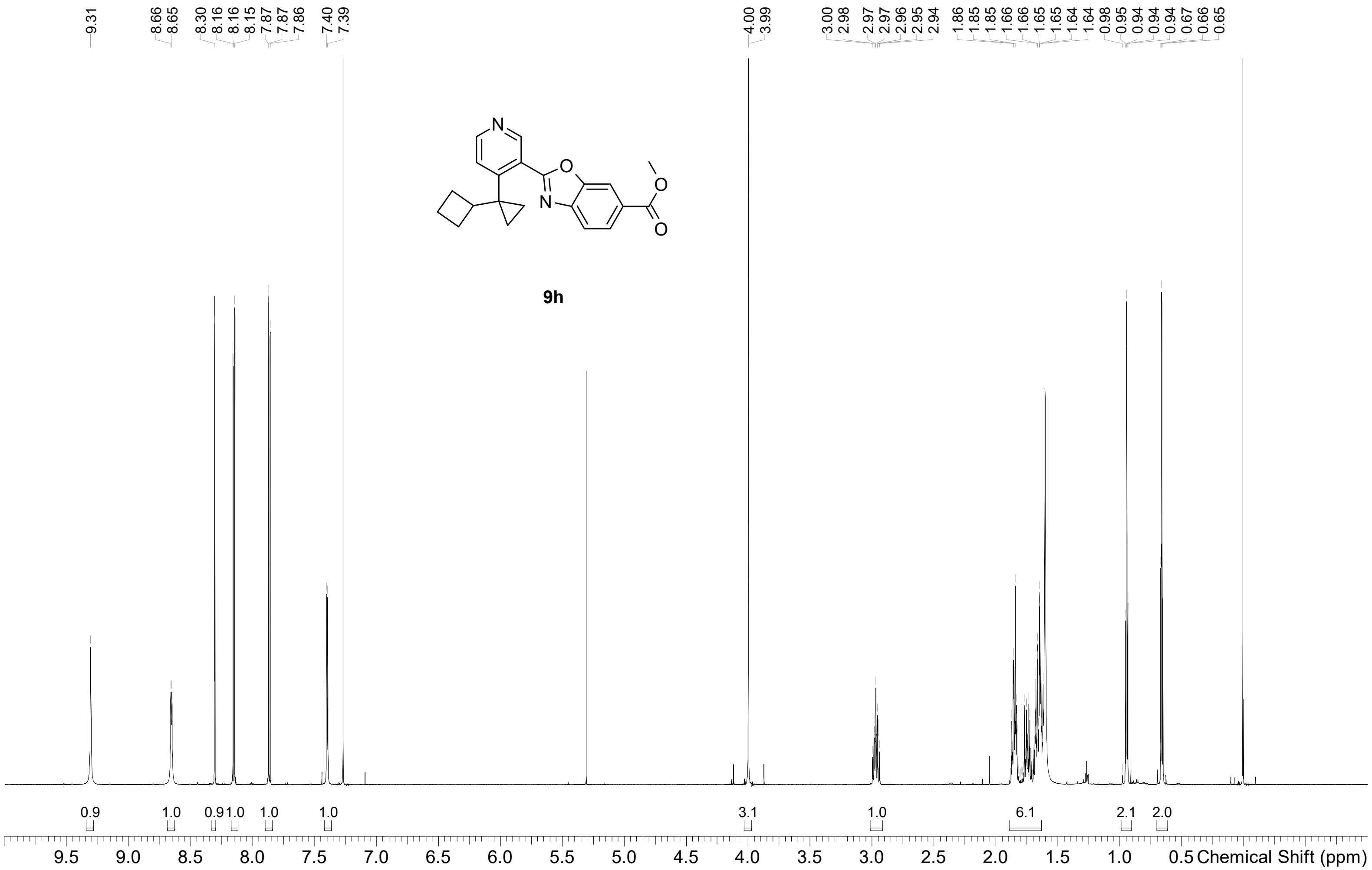

Figure S41: **9h**, ^{1}H-NMR spectra.

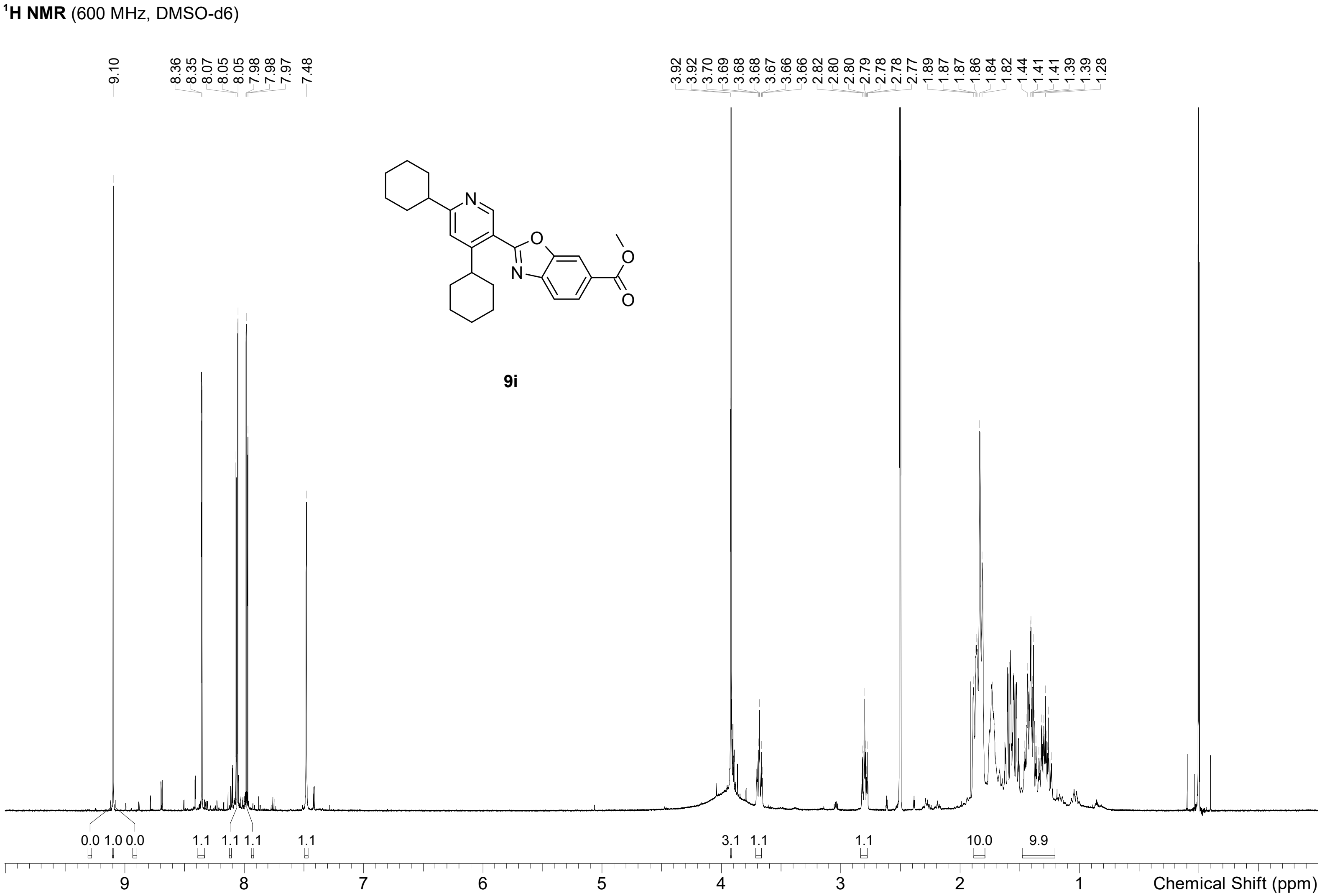

Figure S42: **9i**, ^{1}H-NMR spectra.

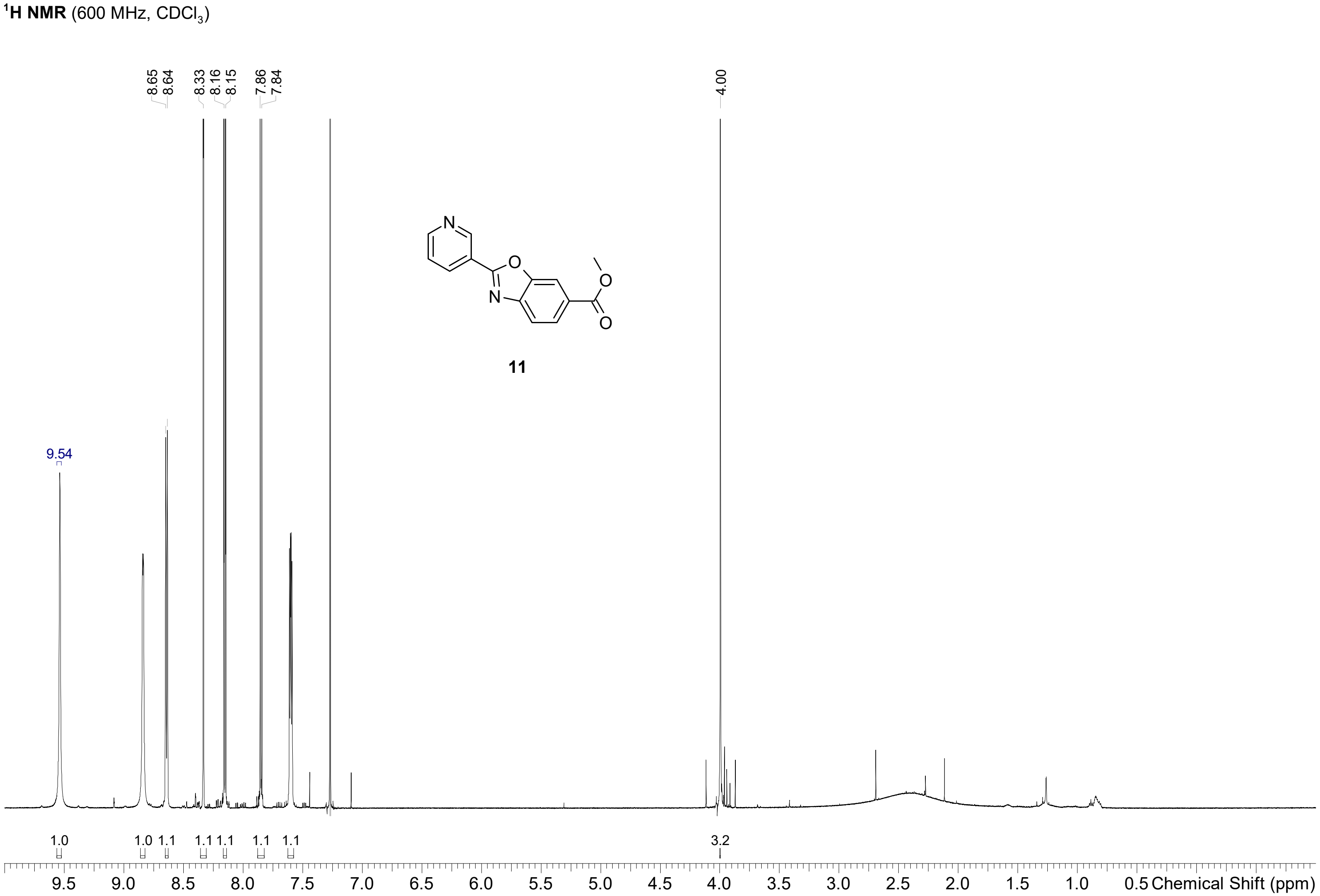

Figure S43: **11**, ^{1}H-NMR spectra.

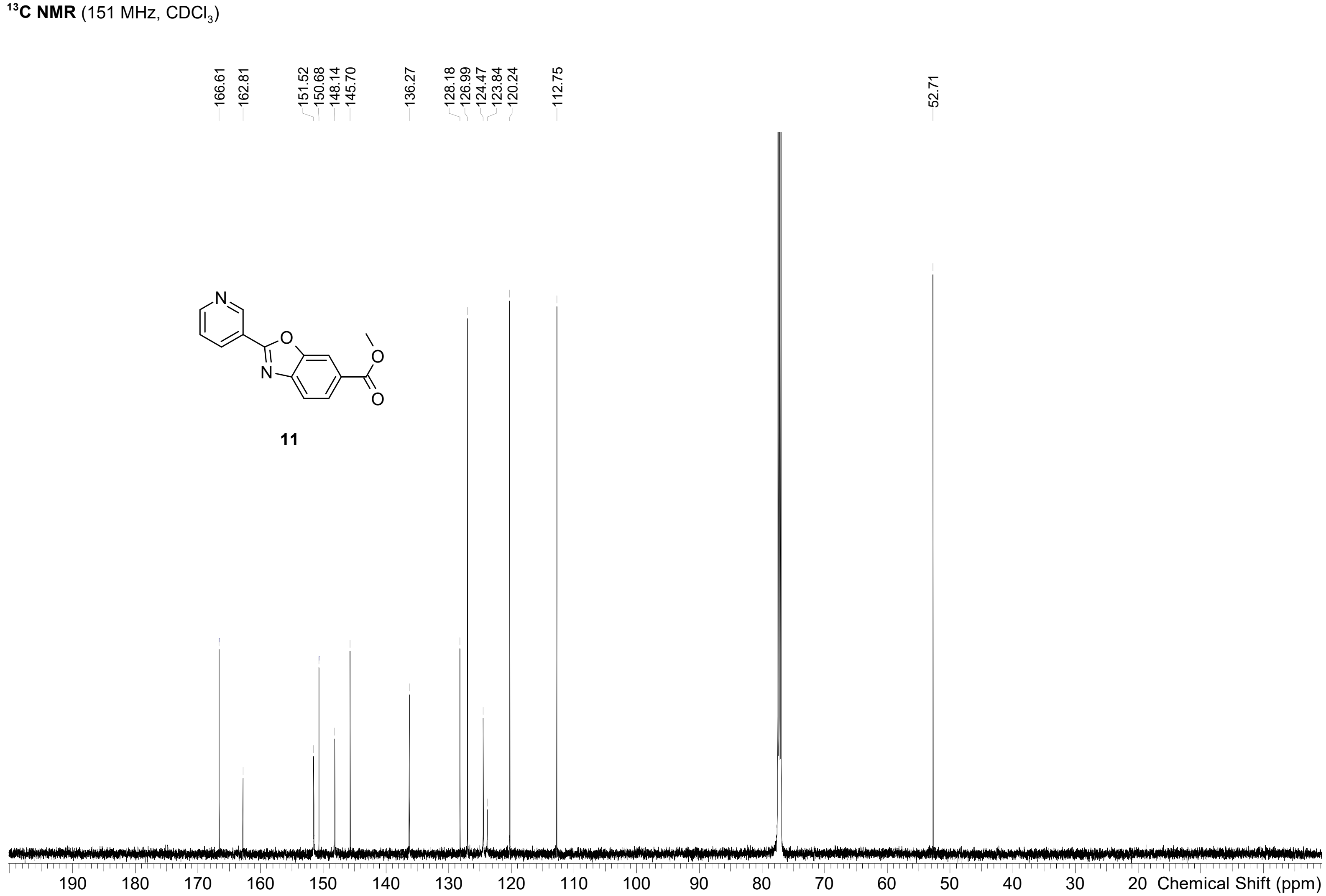

Figure S44: **11**, ^{13}C-NMR spectra.

^{1}**H NMR** (300 MHz, DMSO-d6)

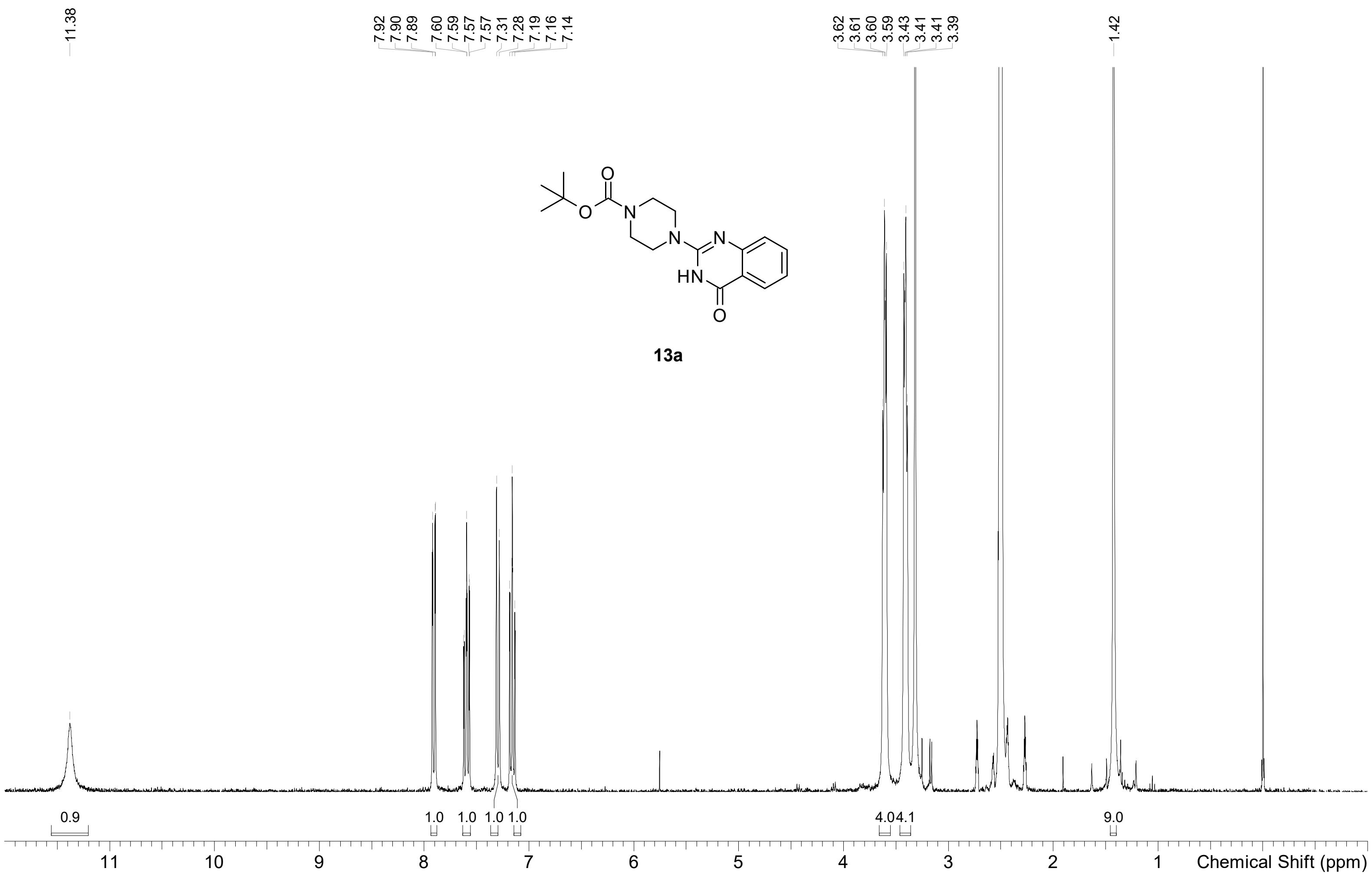

Figure S45: **13a**, ^{1}H-NMR spectra.

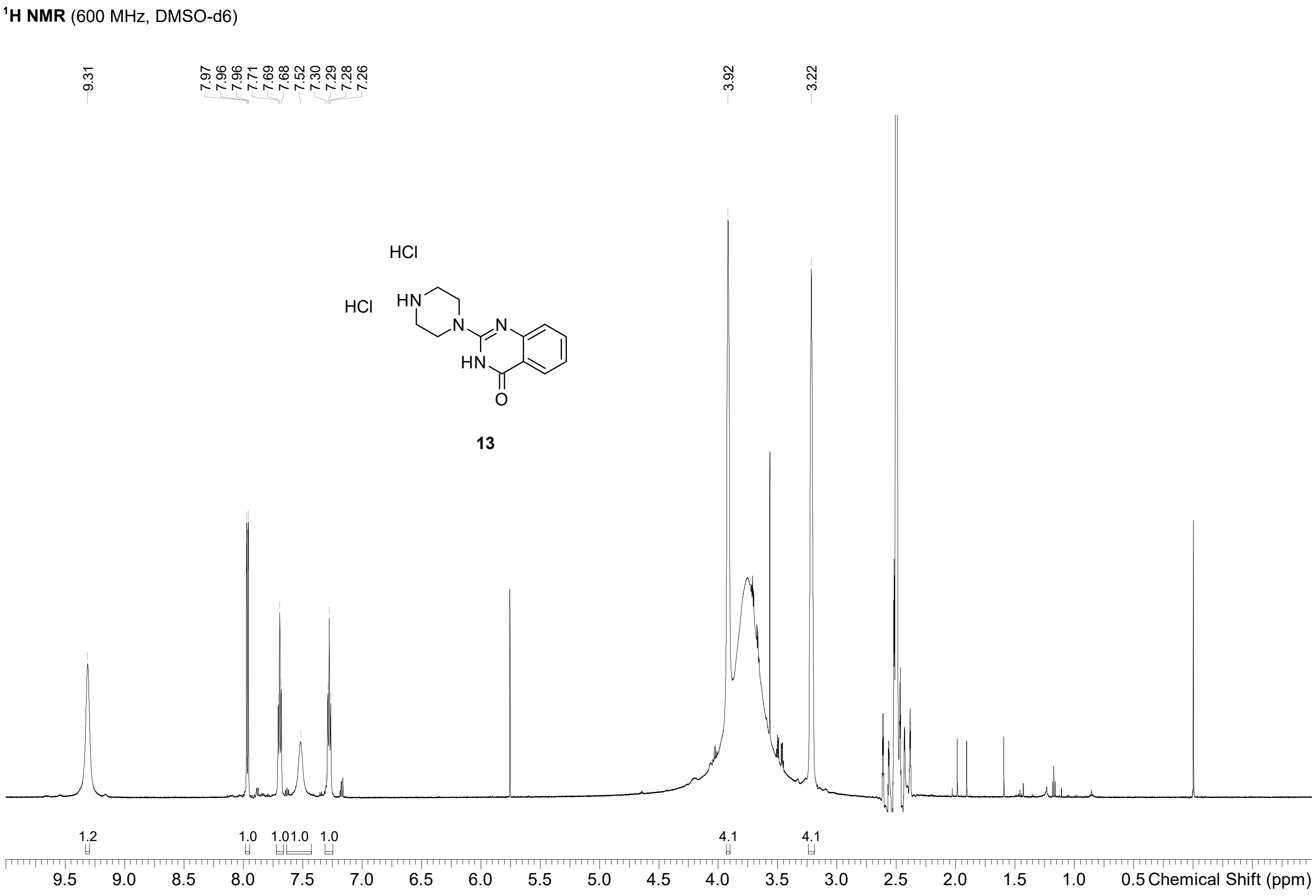

Figure S46: **13**, ^{1}H-NMR spectra.

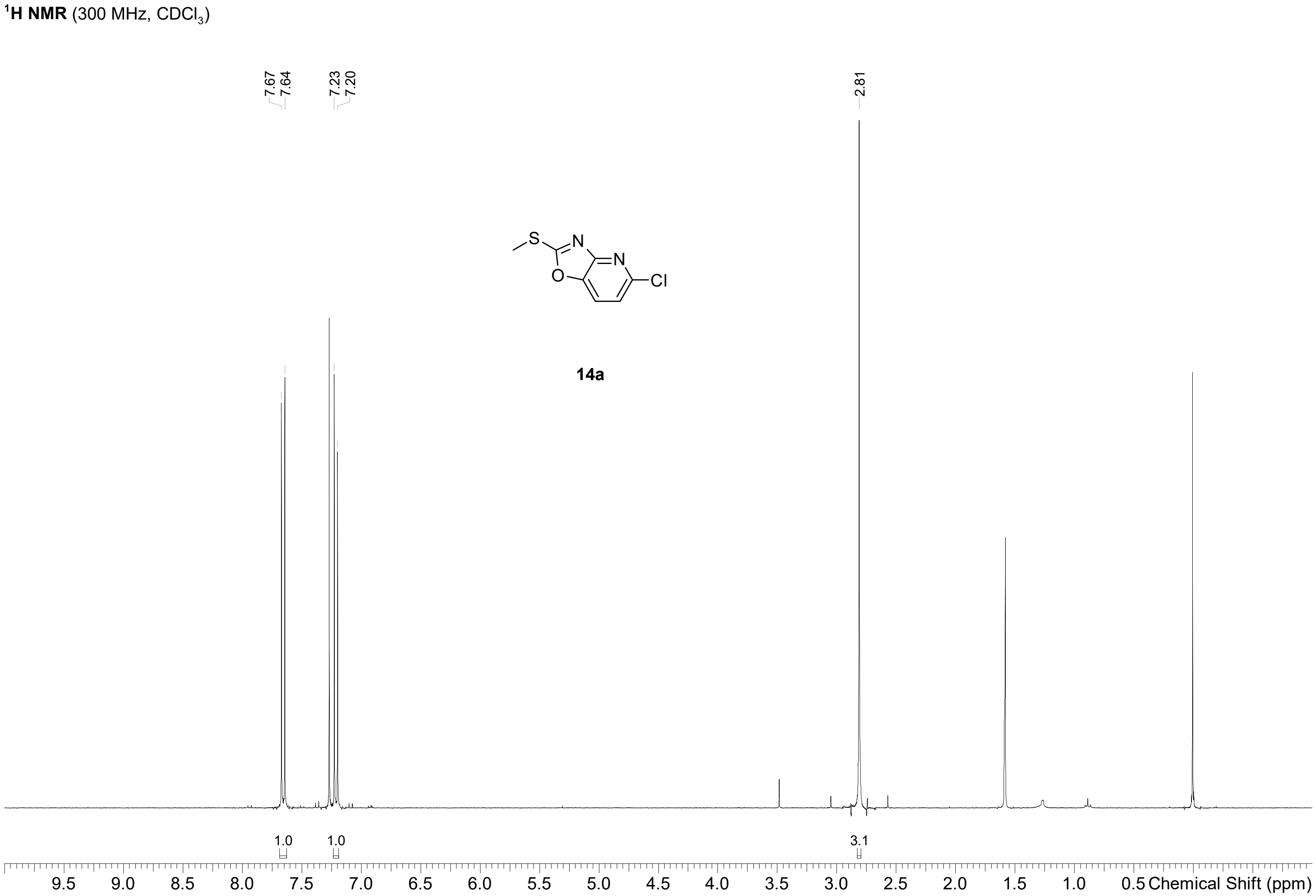

Figure S47: **14a**, ^{1}H-NMR spectra.

^{1}H NMR (300 MHz, $CDCl_3$)

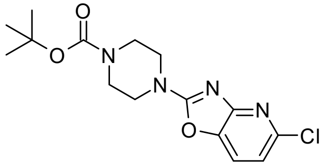

14b

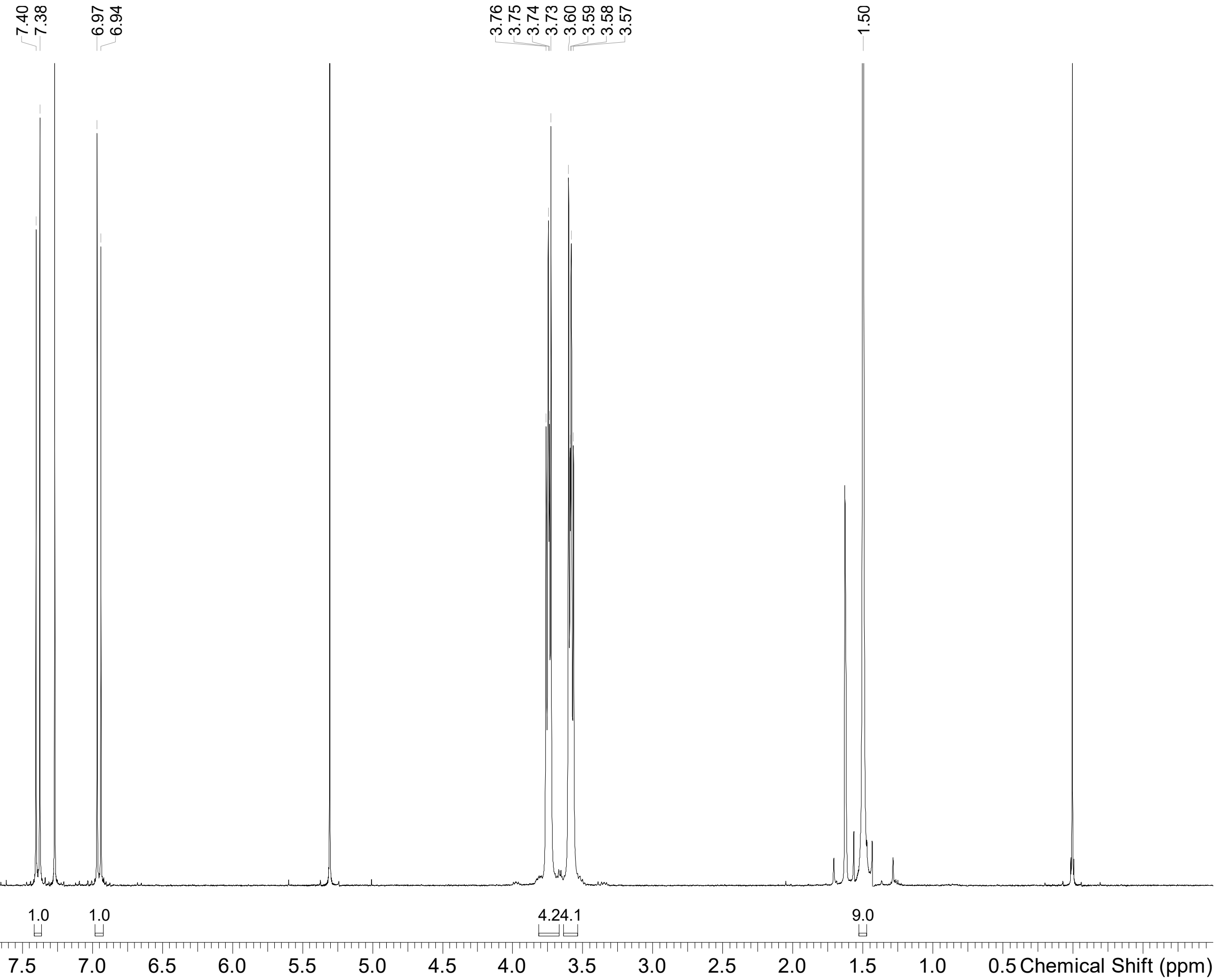

Figure S48: **14b**, ^{1}H-NMR spectra.

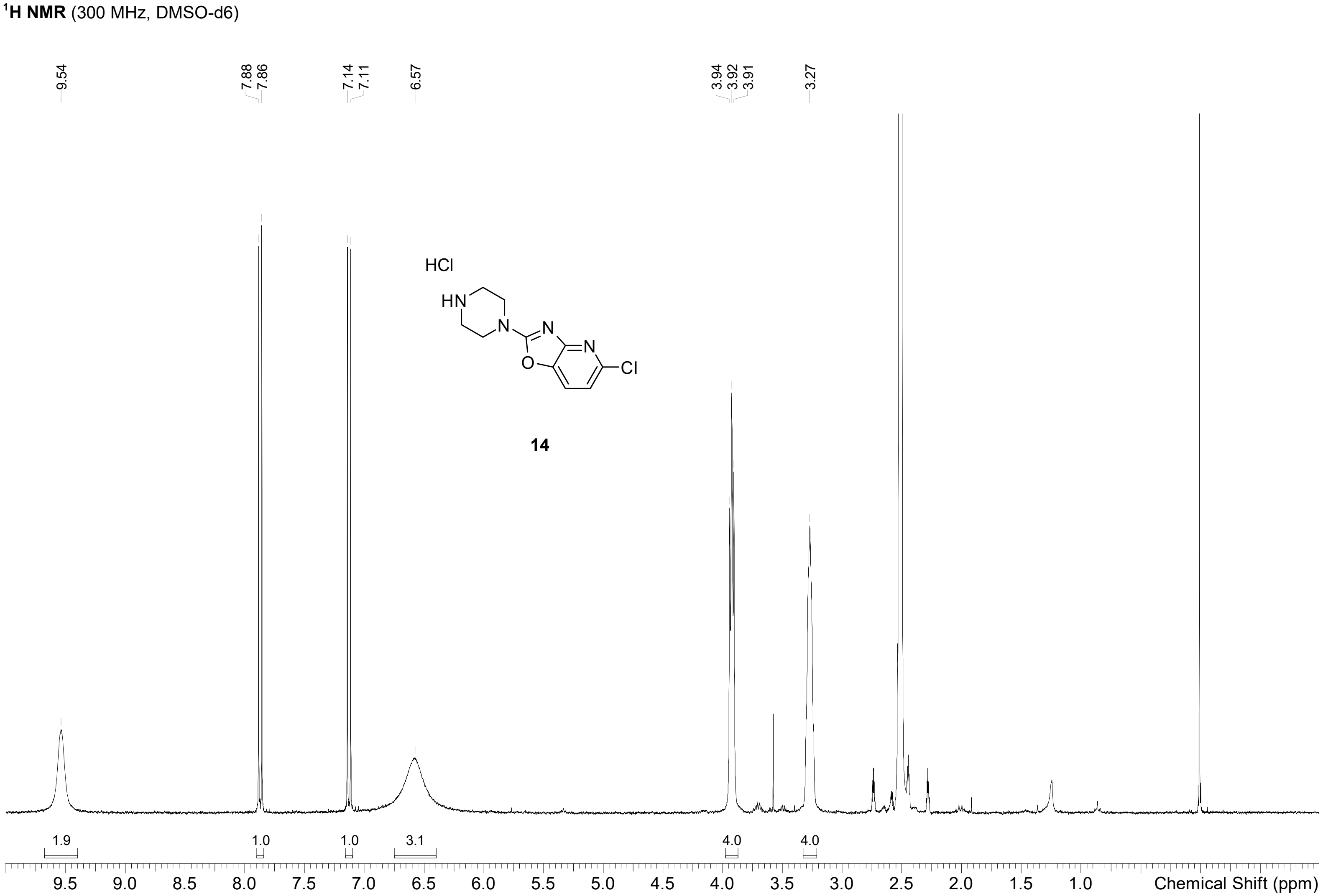

Figure S49: **14**, ^{1}H-NMR spectra.

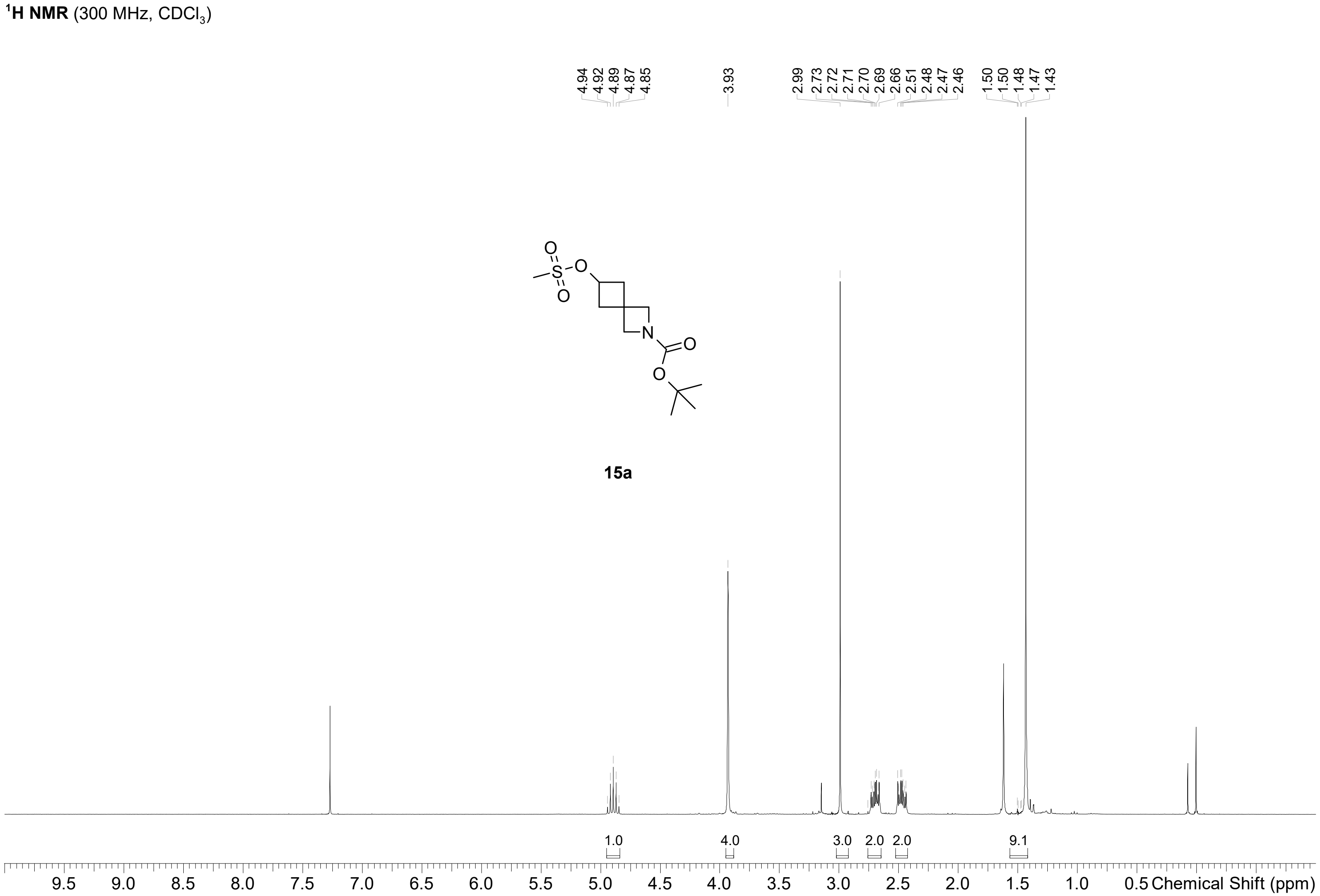

Figure S50: **15a**, ^{1}H-NMR spectra.

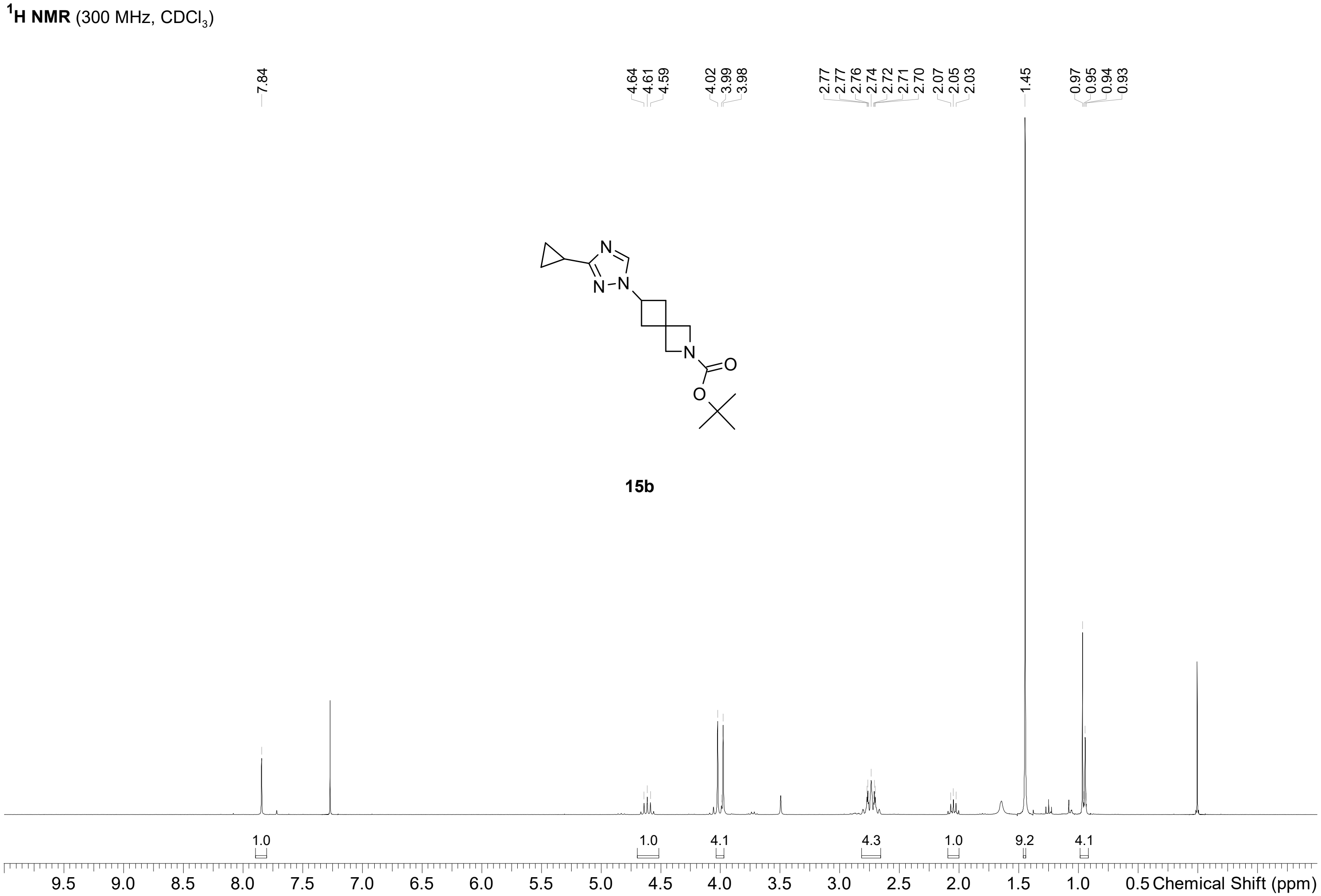

Figure S51: **15b**, ^{1}H-NMR spectra.

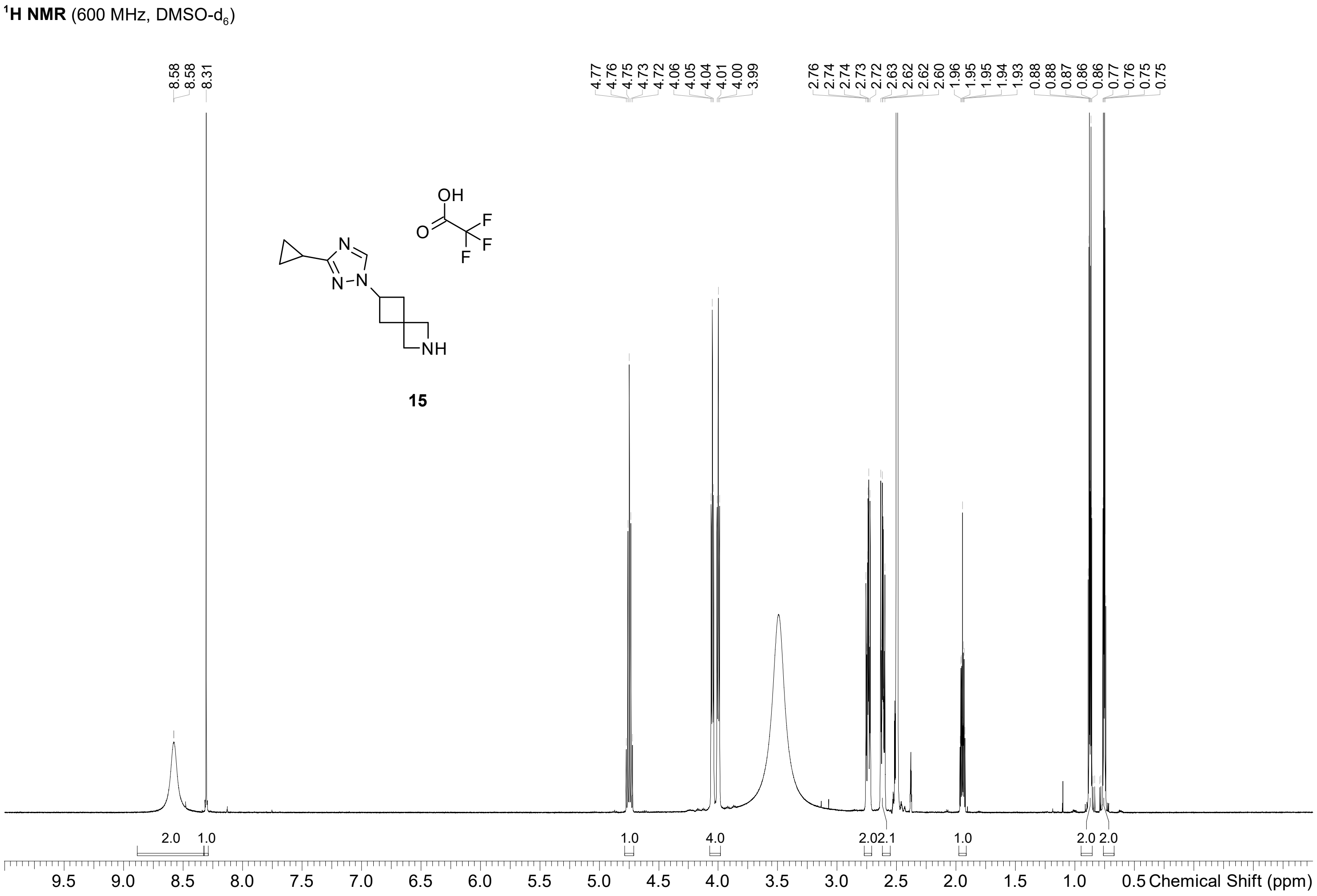

Figure S52: **15**, ^{1}H-NMR spectra.

^{1}H NMR (600 MHz, $CDCl_3$)

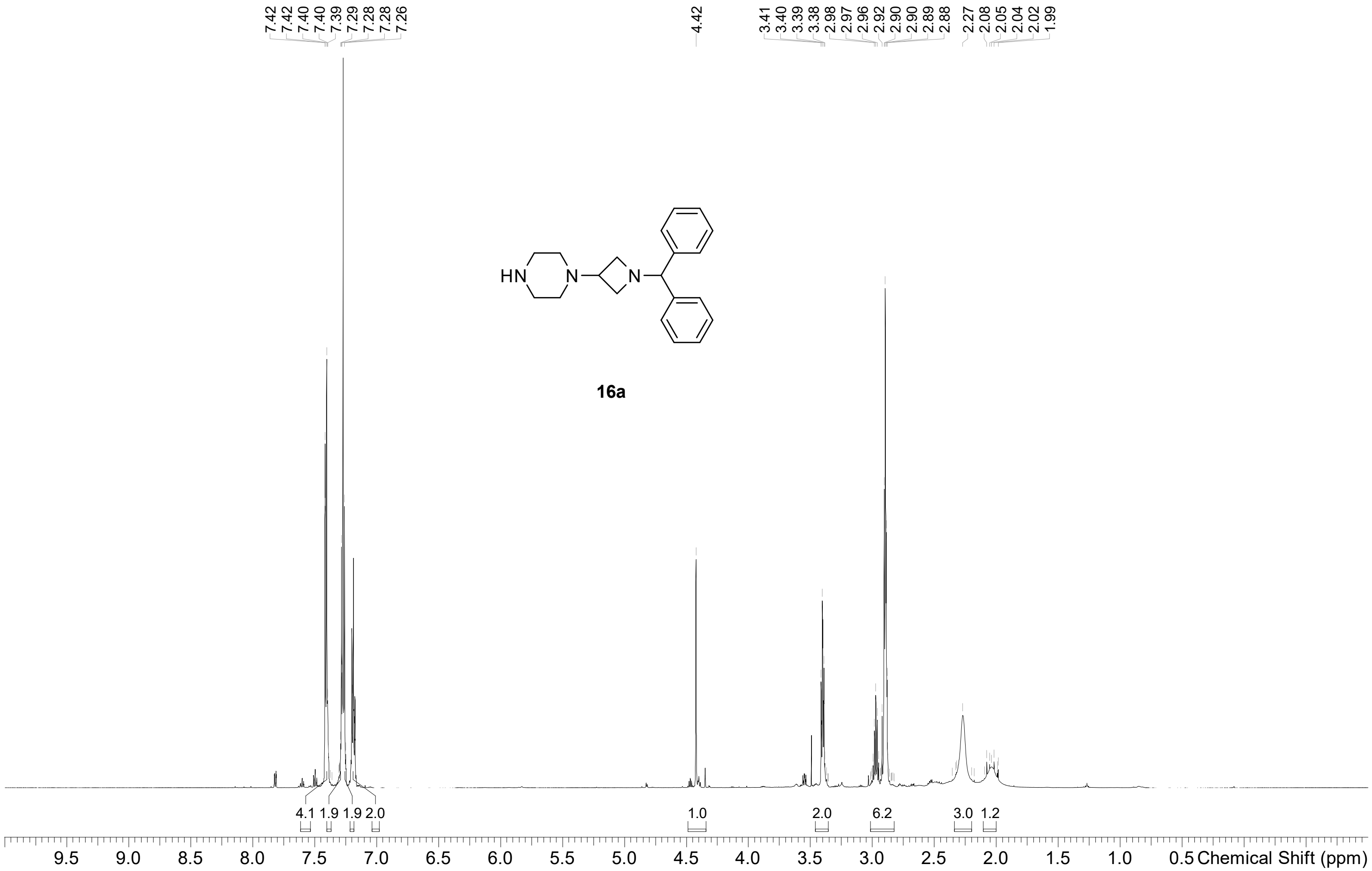

Figure S53: **16a**, ^{1}H-NMR spectra.

^{1}H NMR (300 MHz, $CDCl_3$)

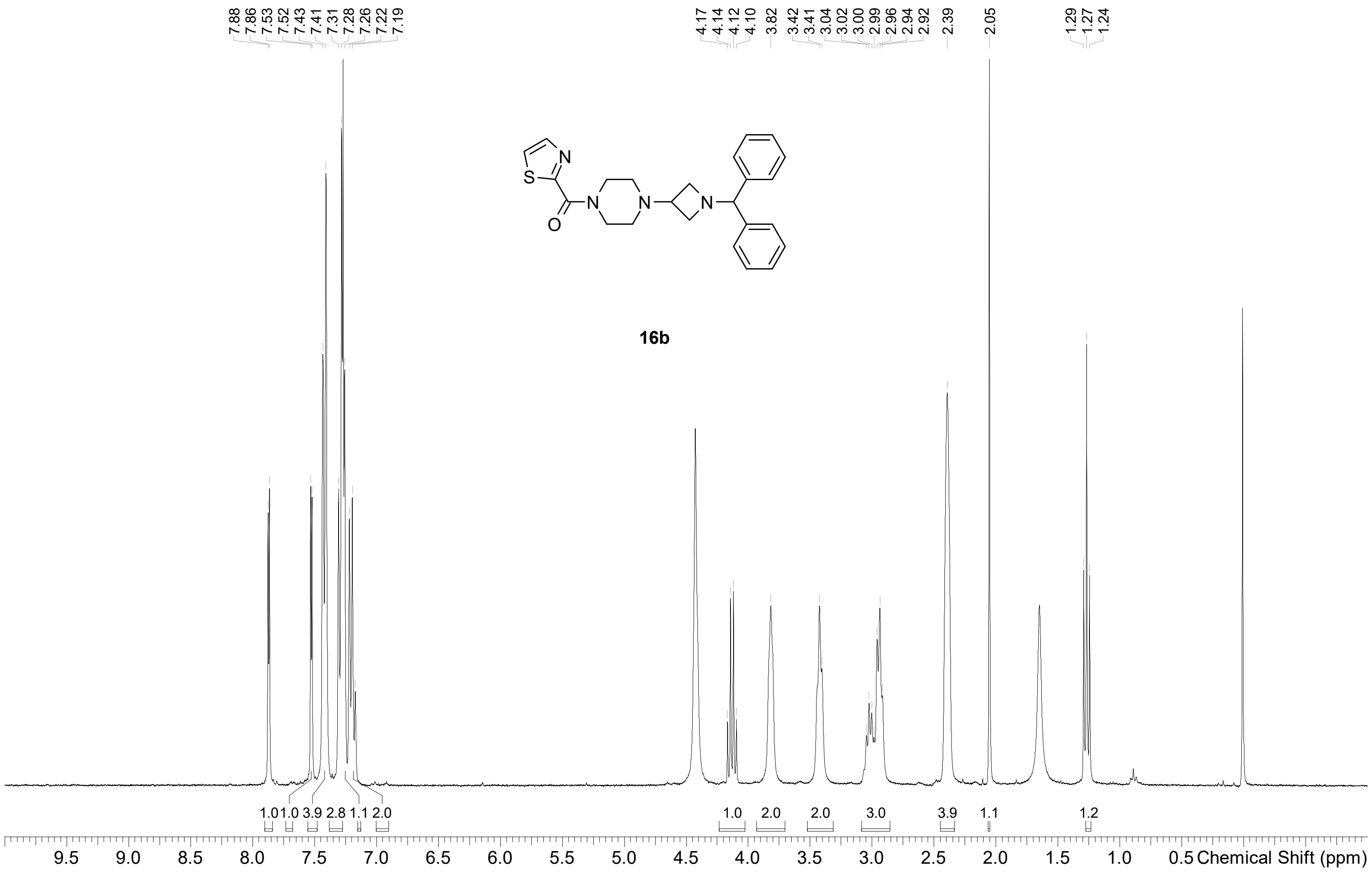

Figure S54: **16b**, ^{1}H-NMR spectra.

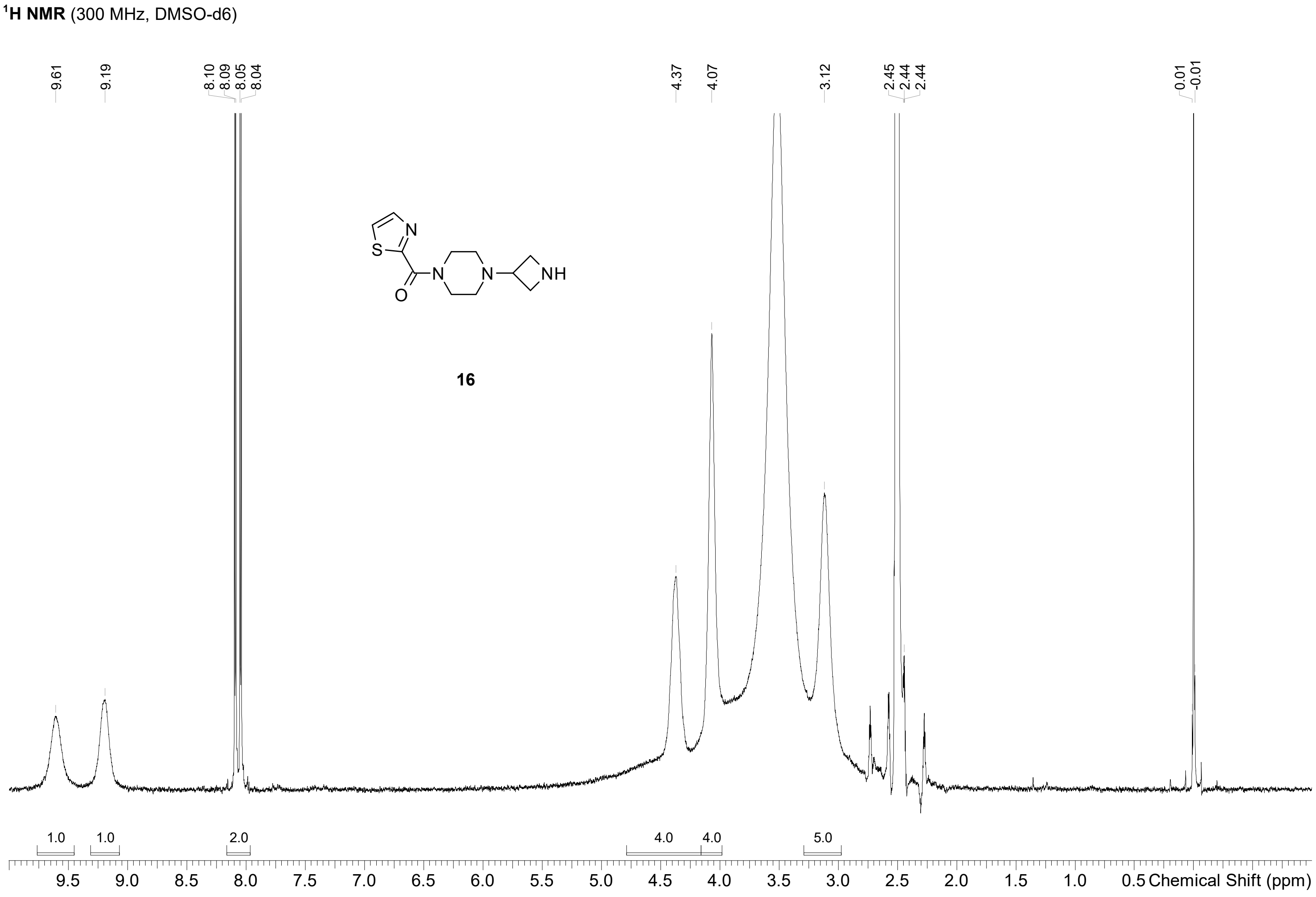

Figure S55: **16**, ¹H-NMR spectra.

^{1}H NMR (600 MHHz, DMSO-d$_6$)

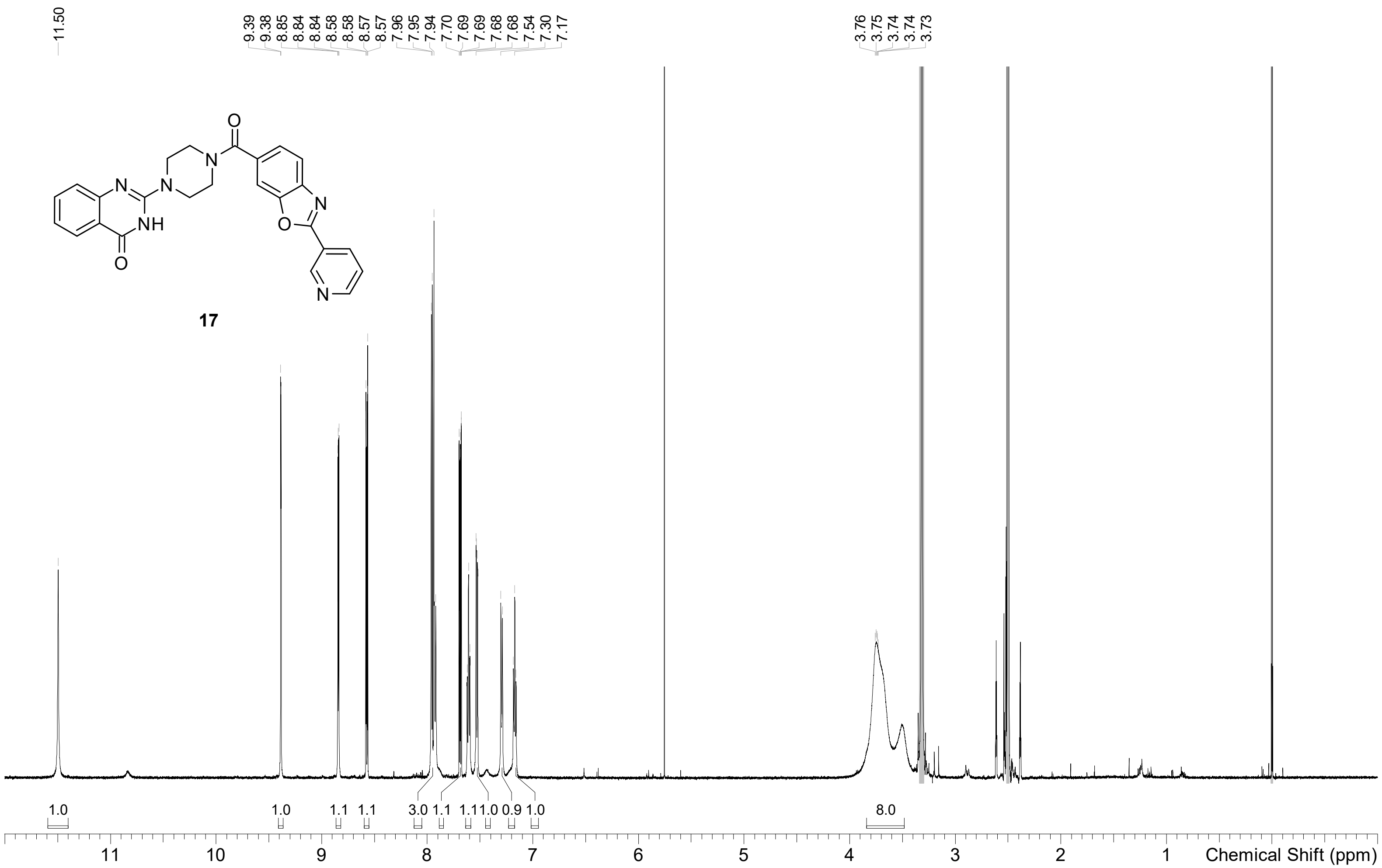

Figure S56: **17**, ^{1}H-NMR spectra.

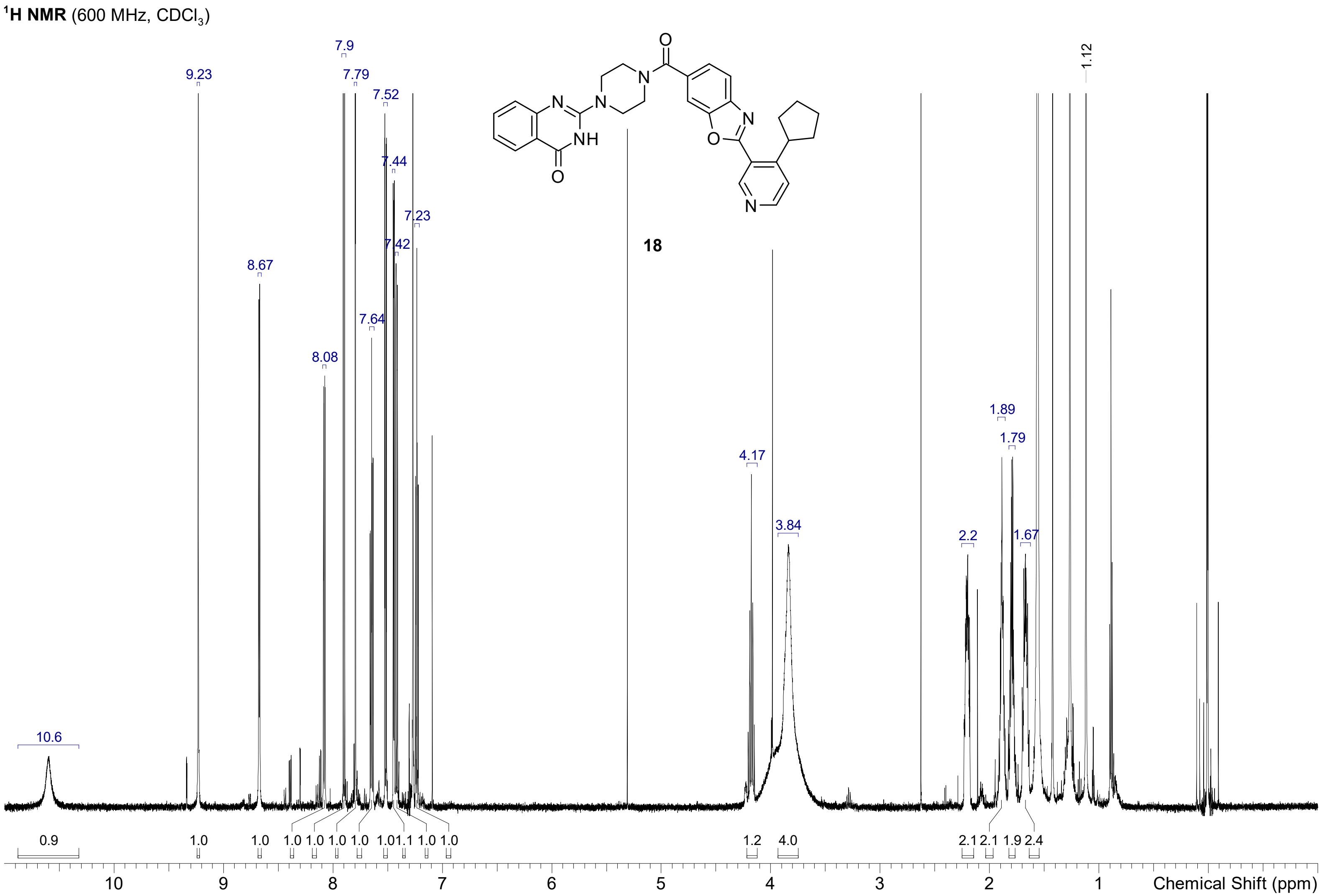

Figure S57: **18**, ^{1}H-NMR spectra.

^{13}**C NMR** (151 MHz, $CDCl_3$)

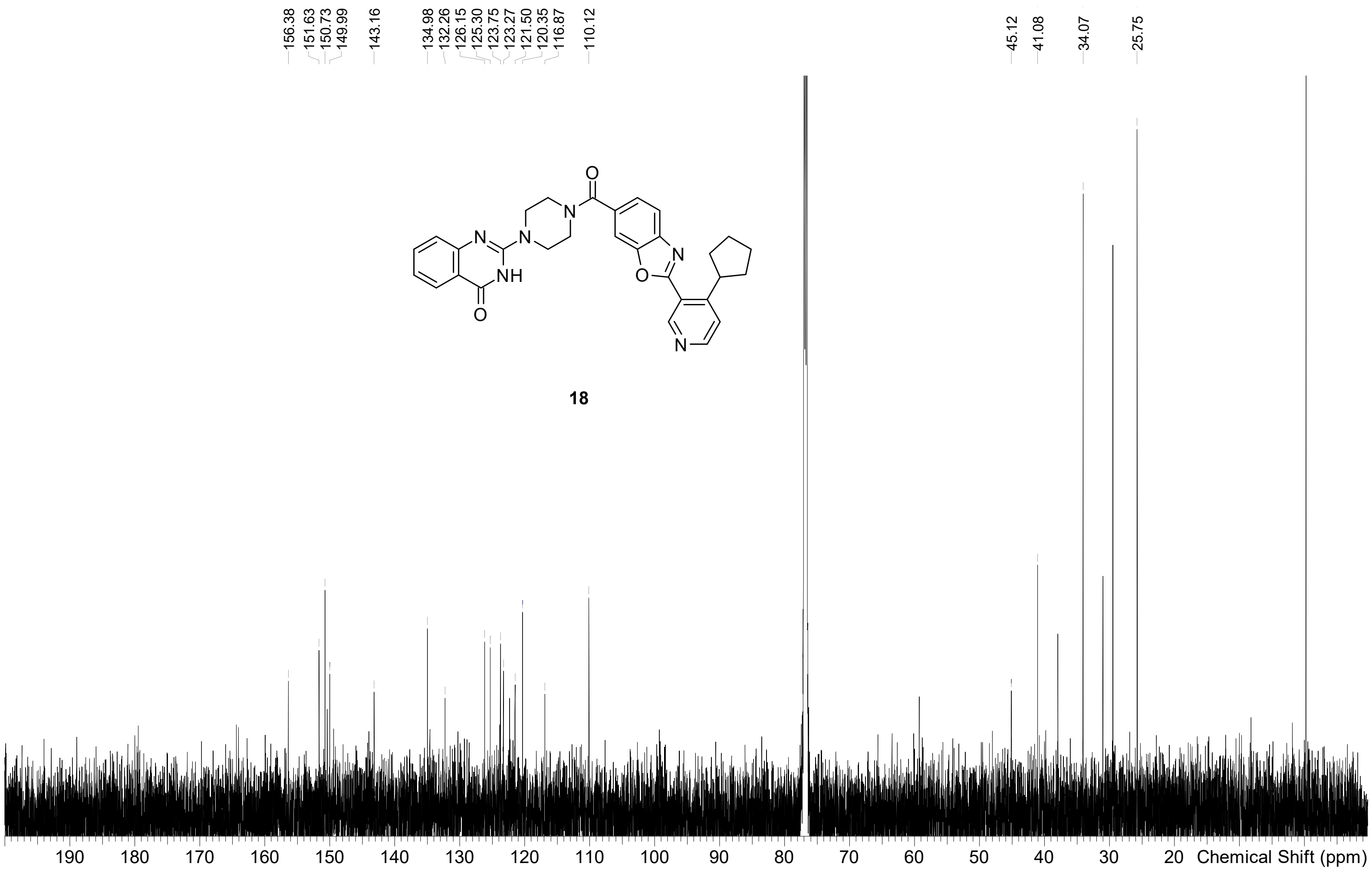

Figure S58: **18**, ^{13}C-NMR spectra.

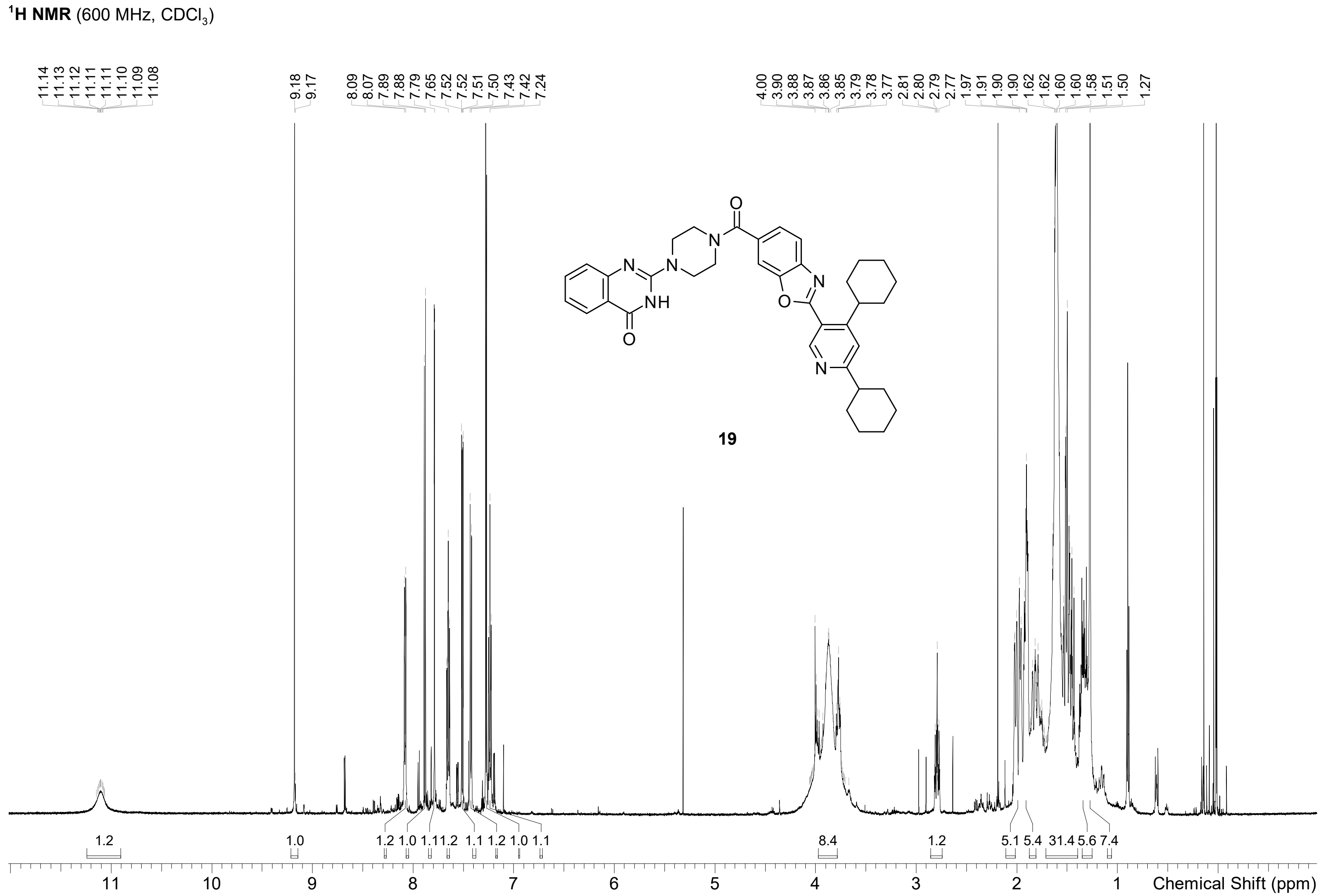

Figure S59: **19**, ^{1}H-NMR spectra.

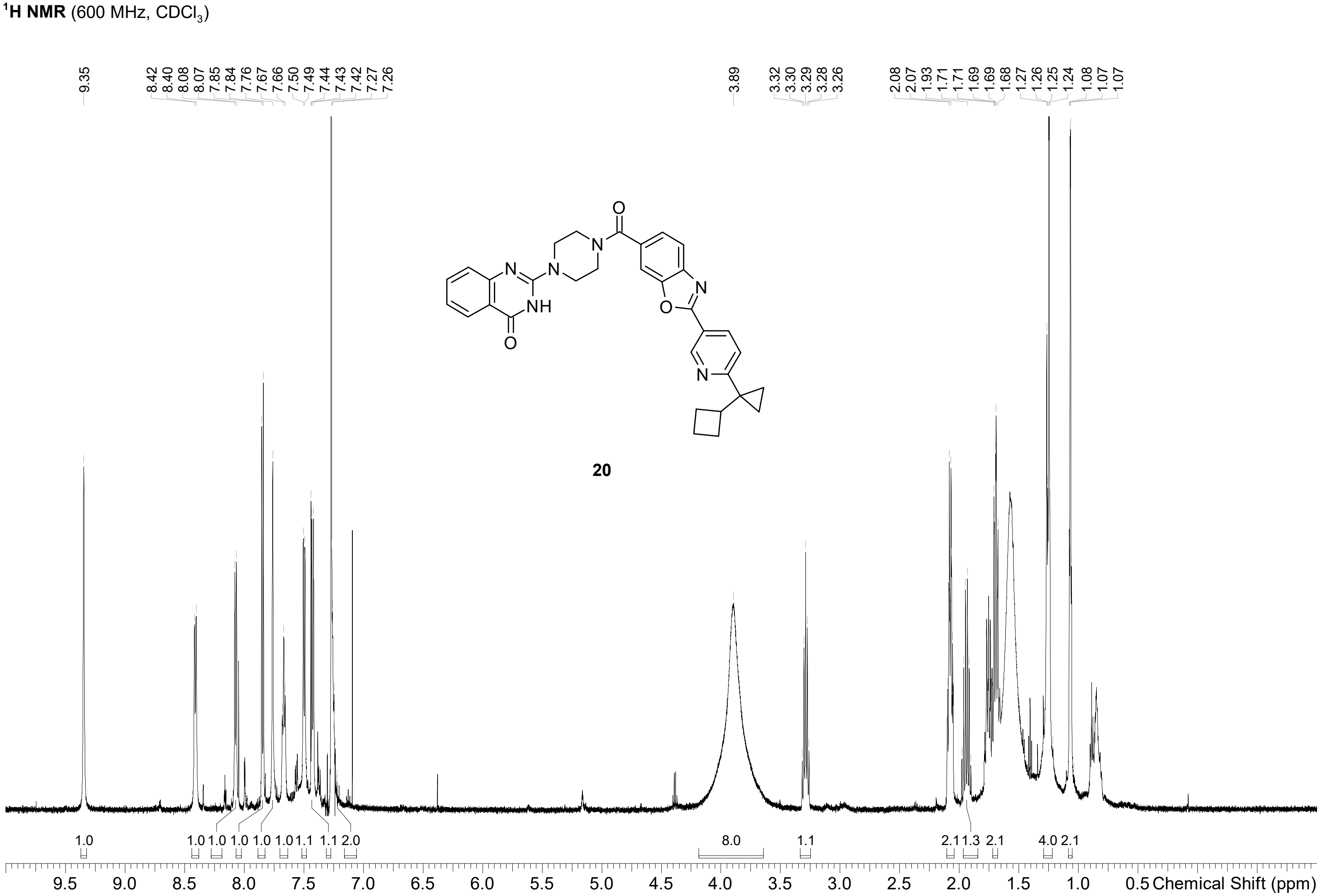

Figure S60: **20**, ^{1}H-NMR spectra.

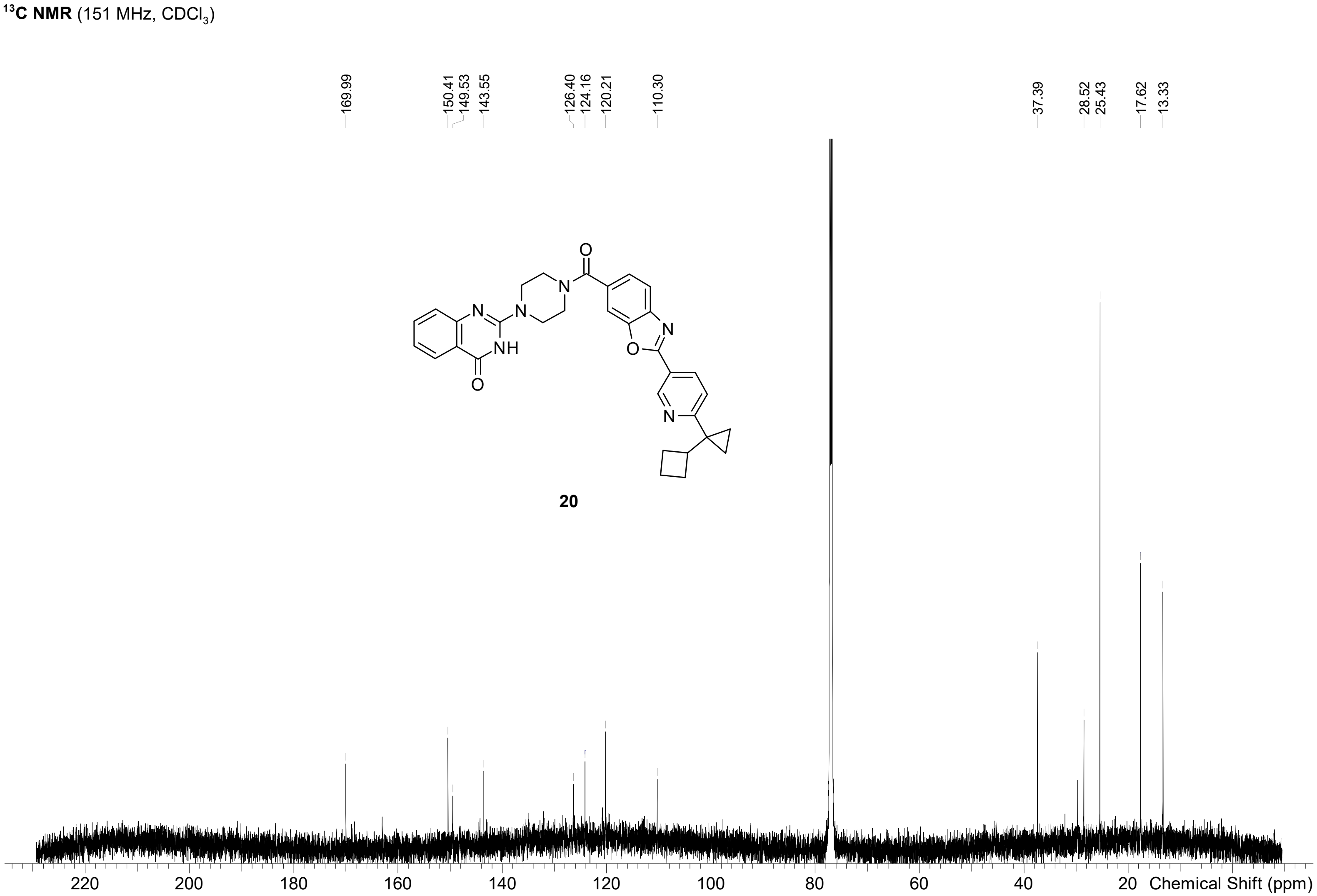

Figure S61: **20**, ^{13}C-NMR spectra.

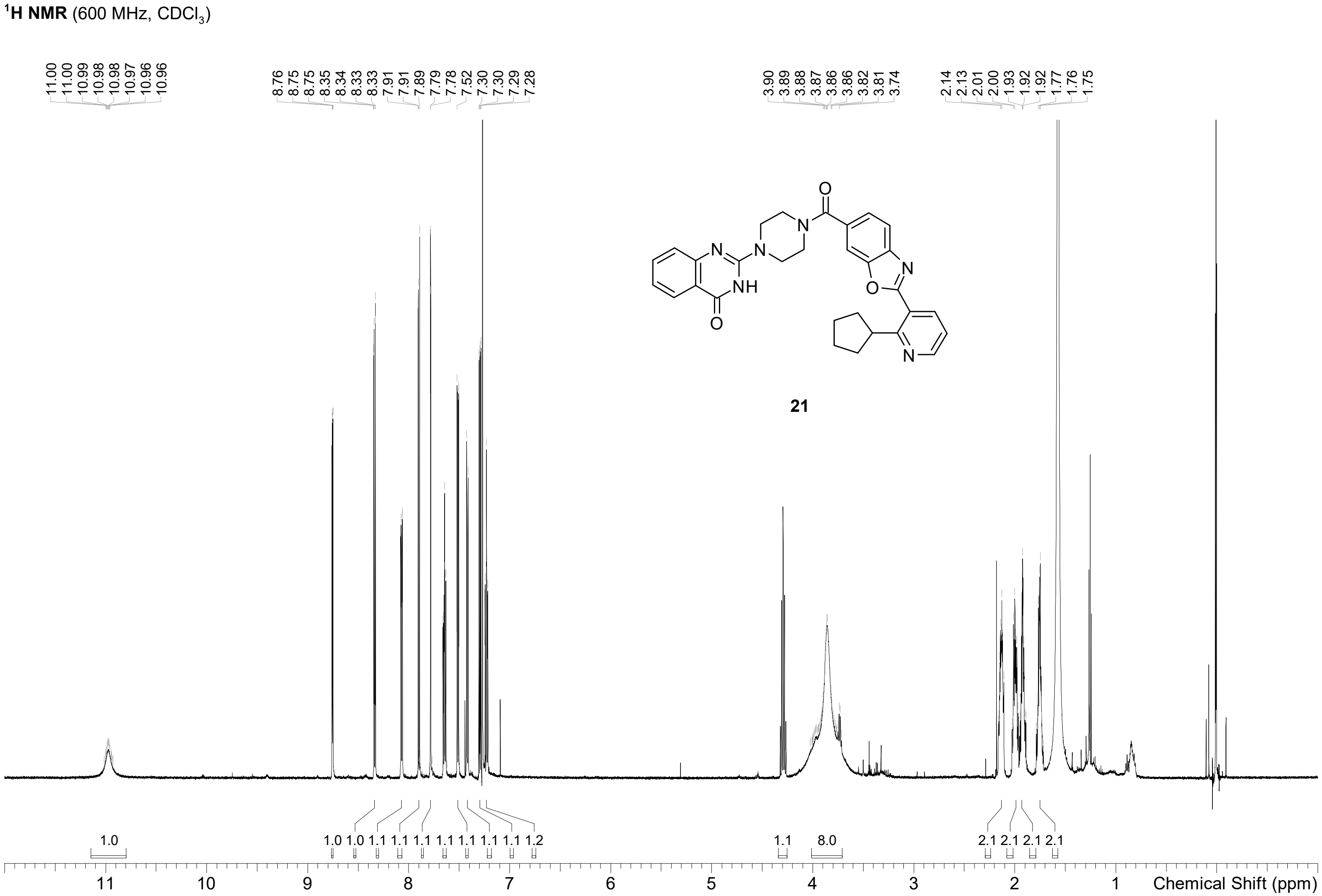

Figure S62: **21**, ^{1}H-NMR spectra.

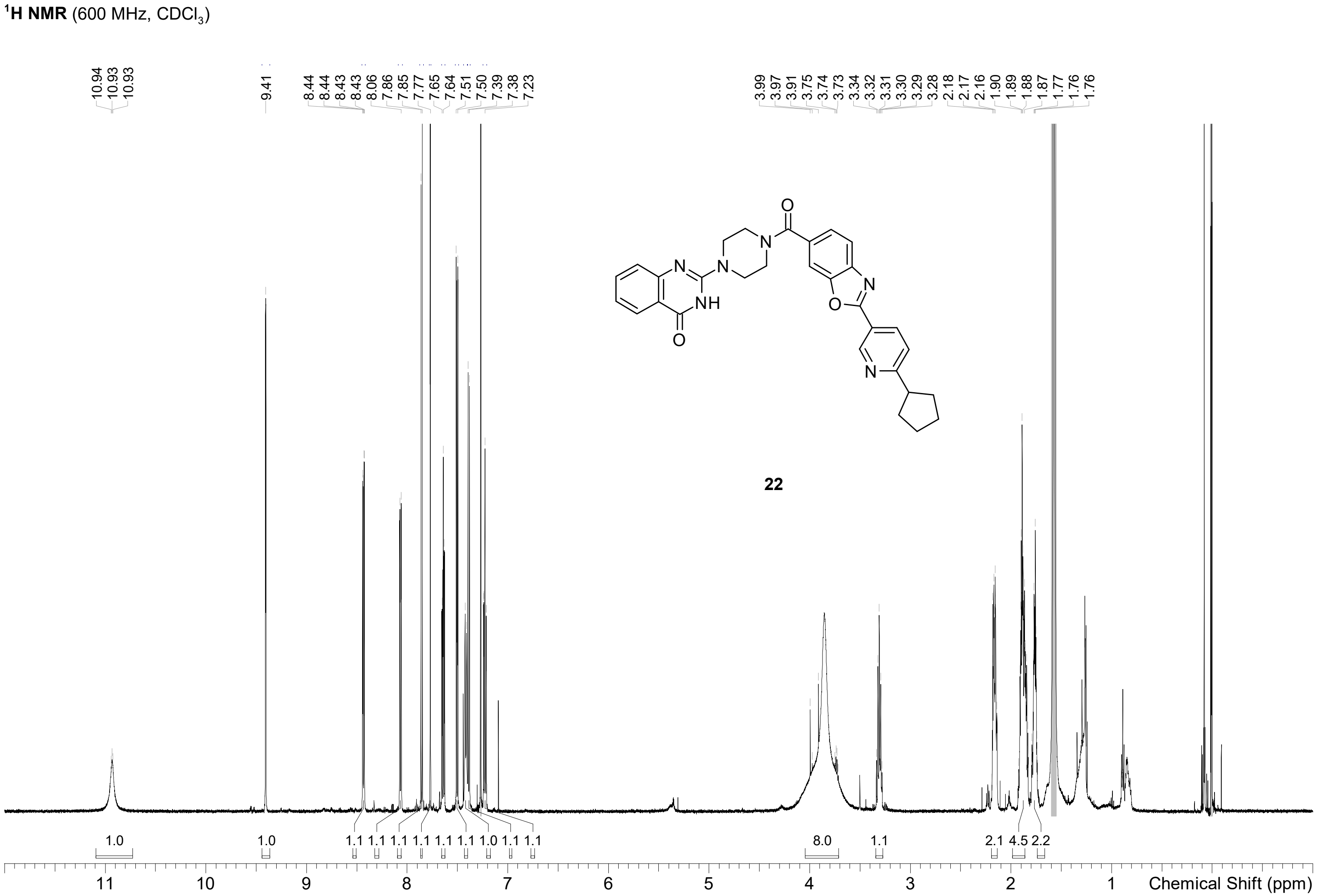

Figure S63: **22**, ^{1}H-NMR spectra.

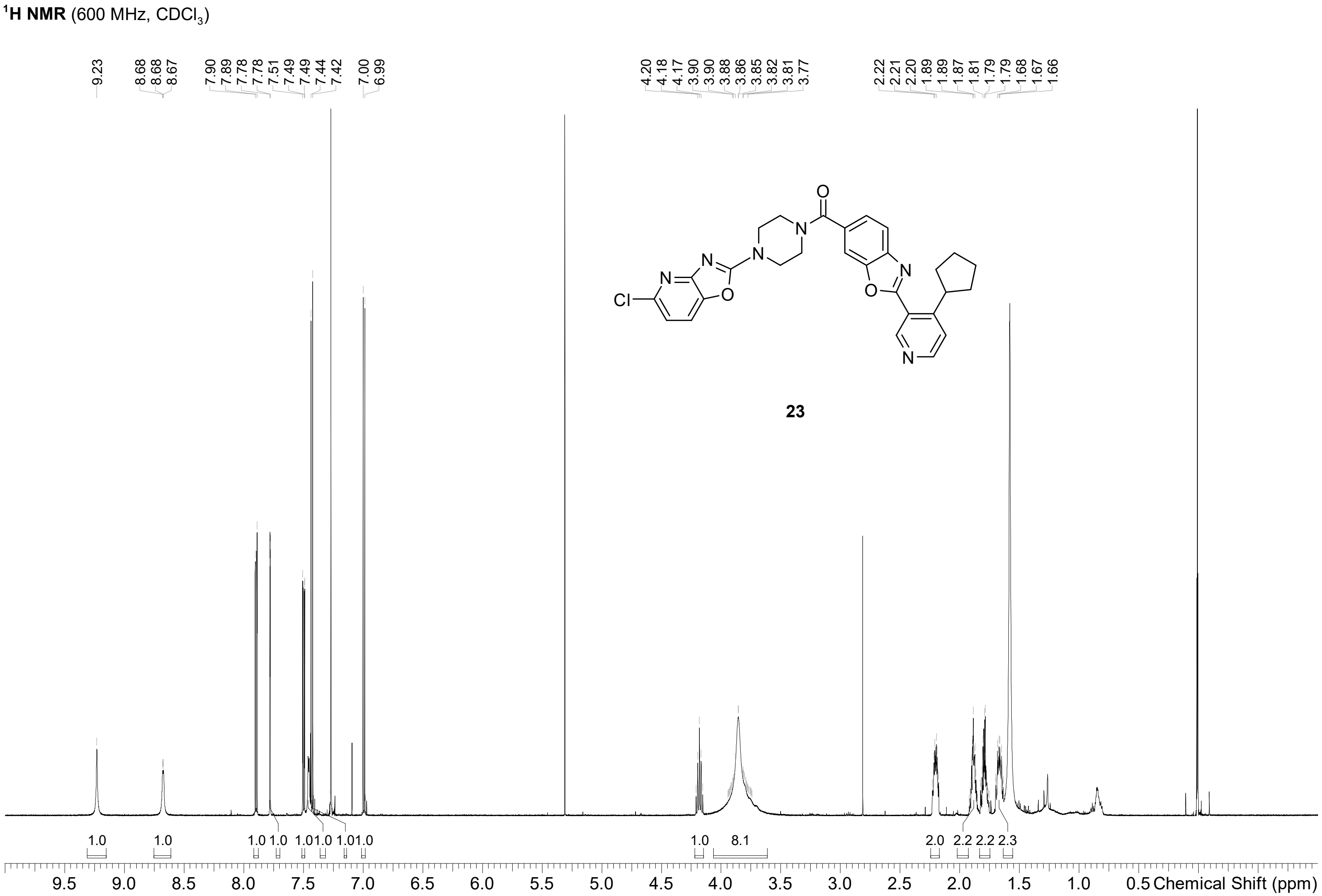

Figure S64: **23**, ^{1}H-NMR spectra.

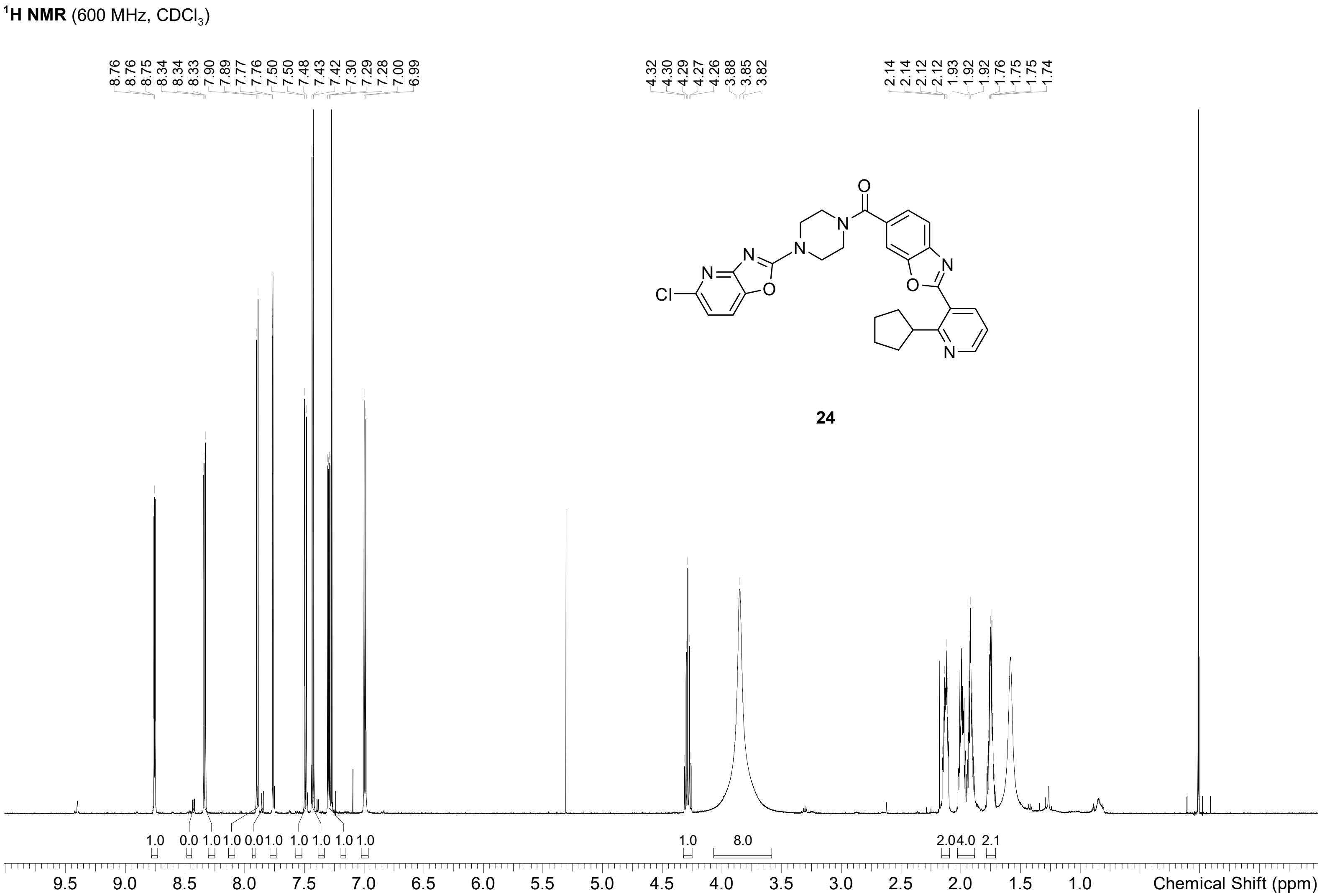

Figure S65: **24**, ^{1}H-NMR spectra.

^{1}H NMR (600 MHz, DMSO-d6)

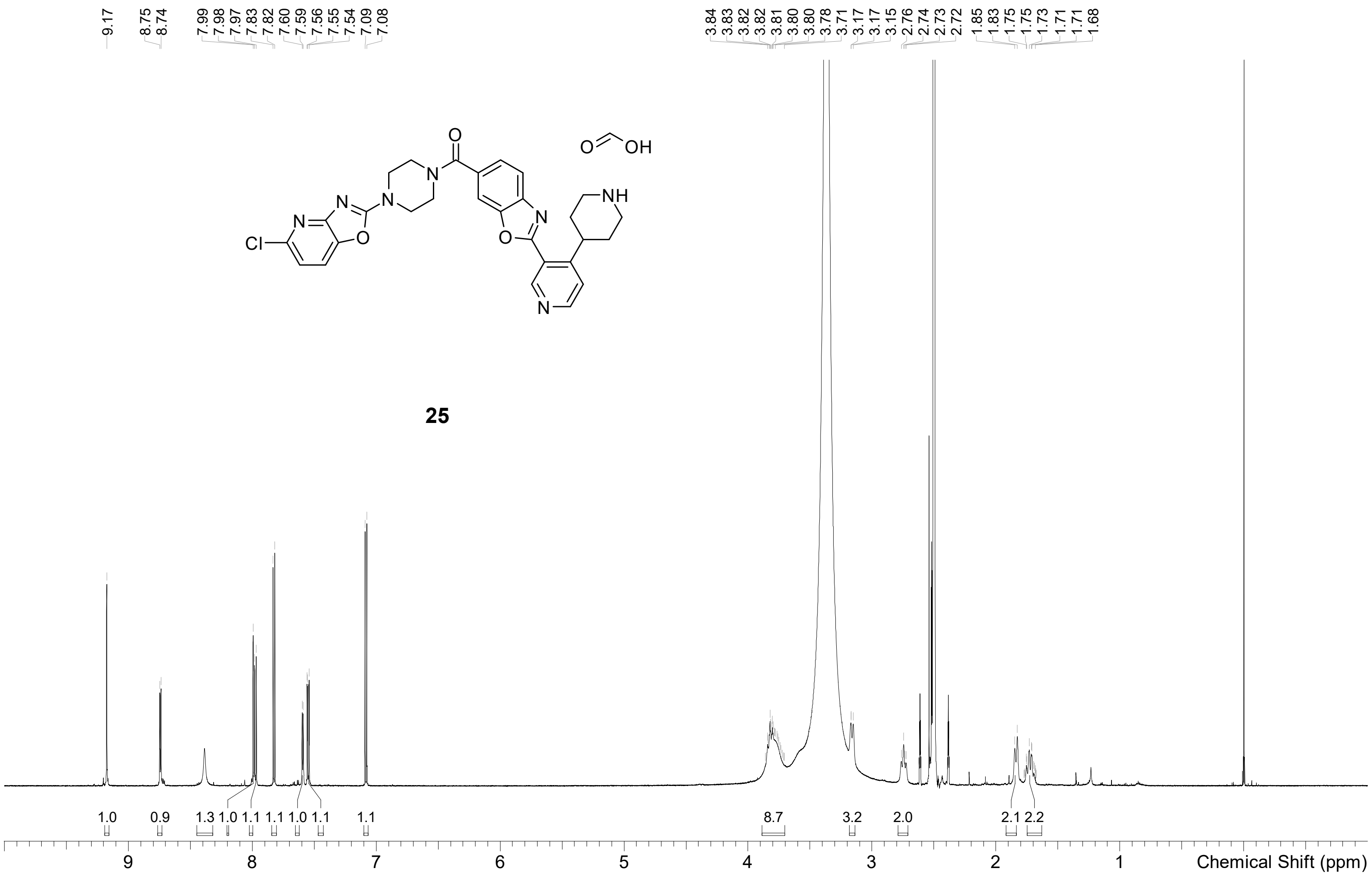

Figure S66: **25**, ^{1}H-NMR spectra.

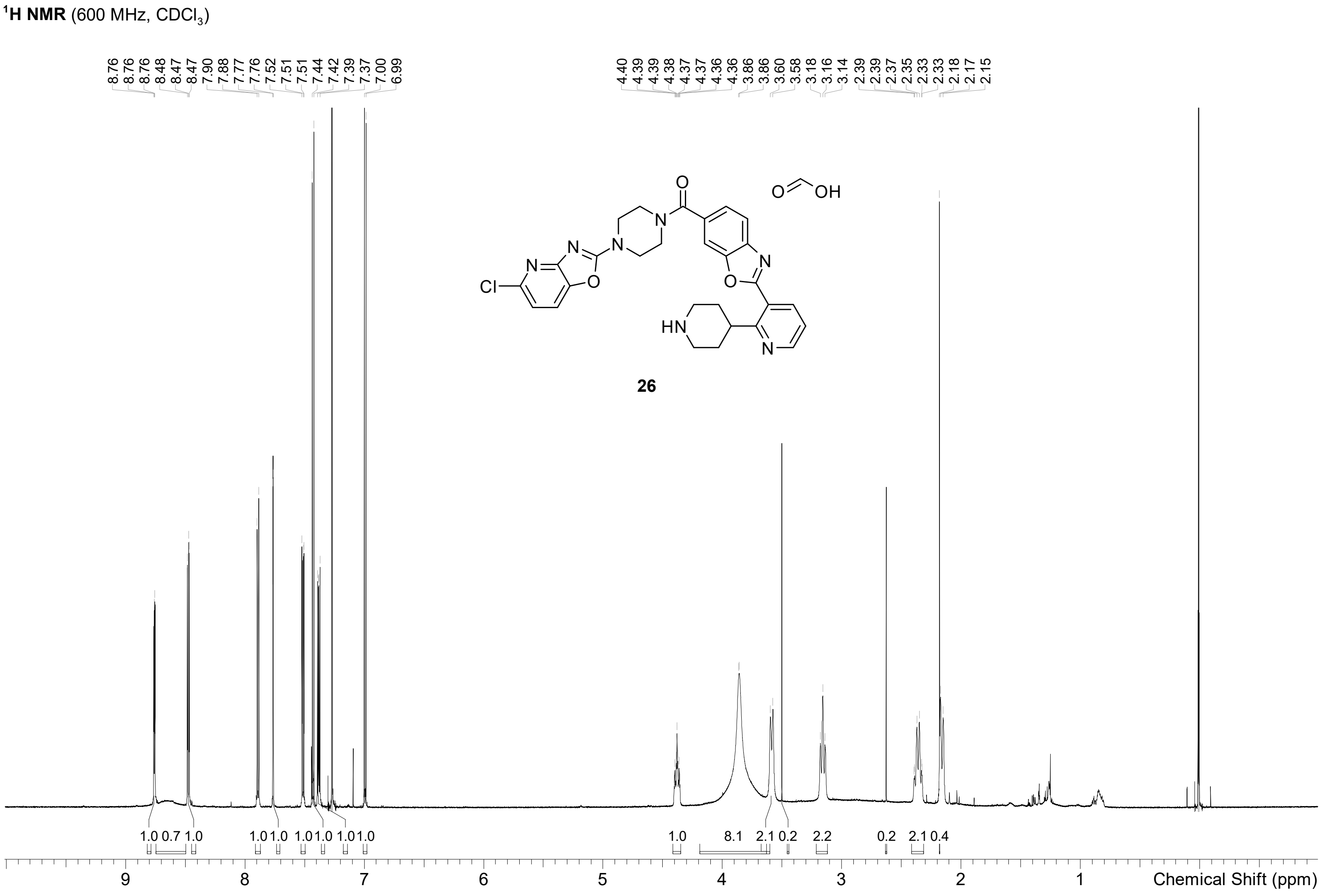

Figure S67: **26**, ^{1}H-NMR spectra.

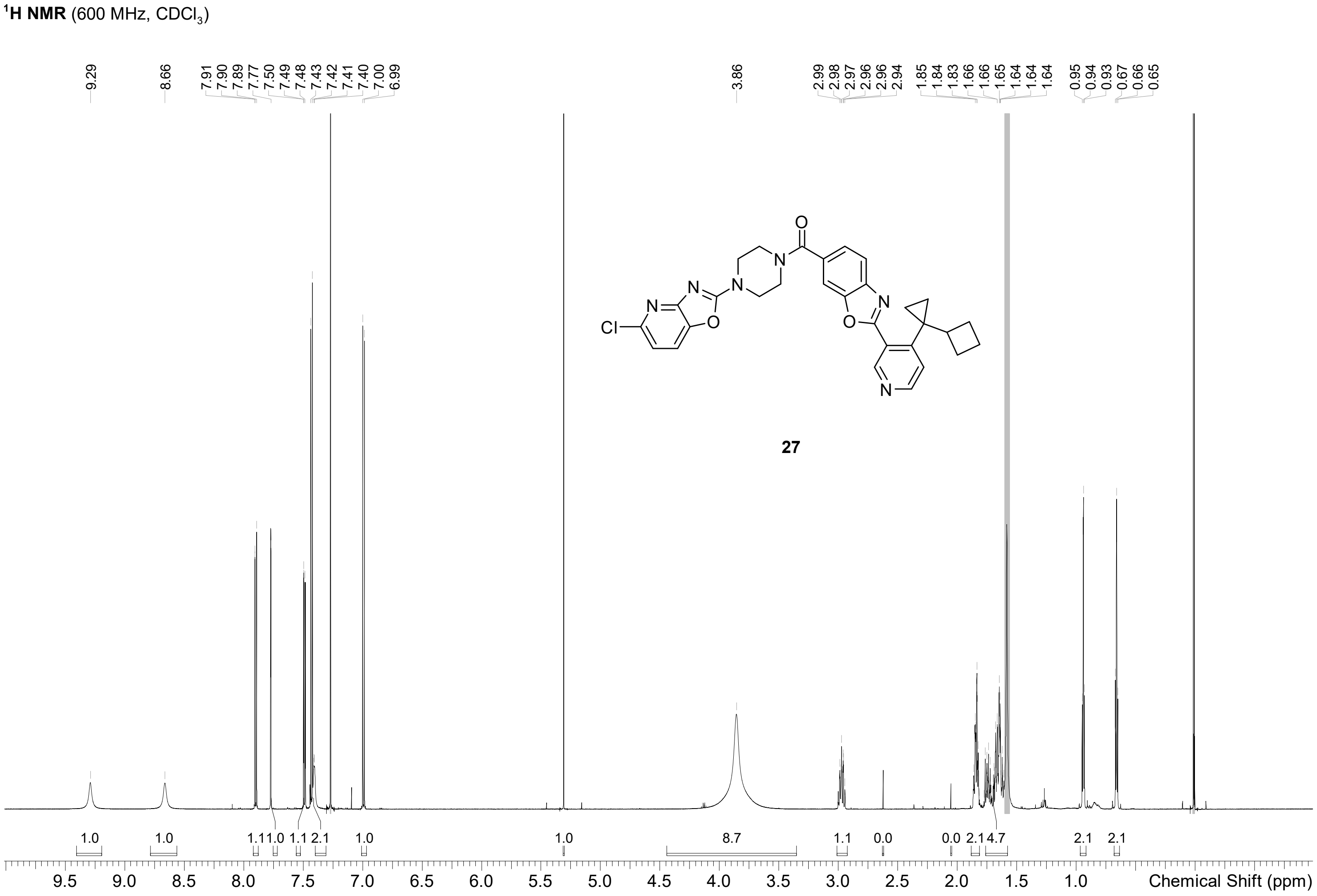

Figure S68: **27**, ^{1}H-NMR spectra.

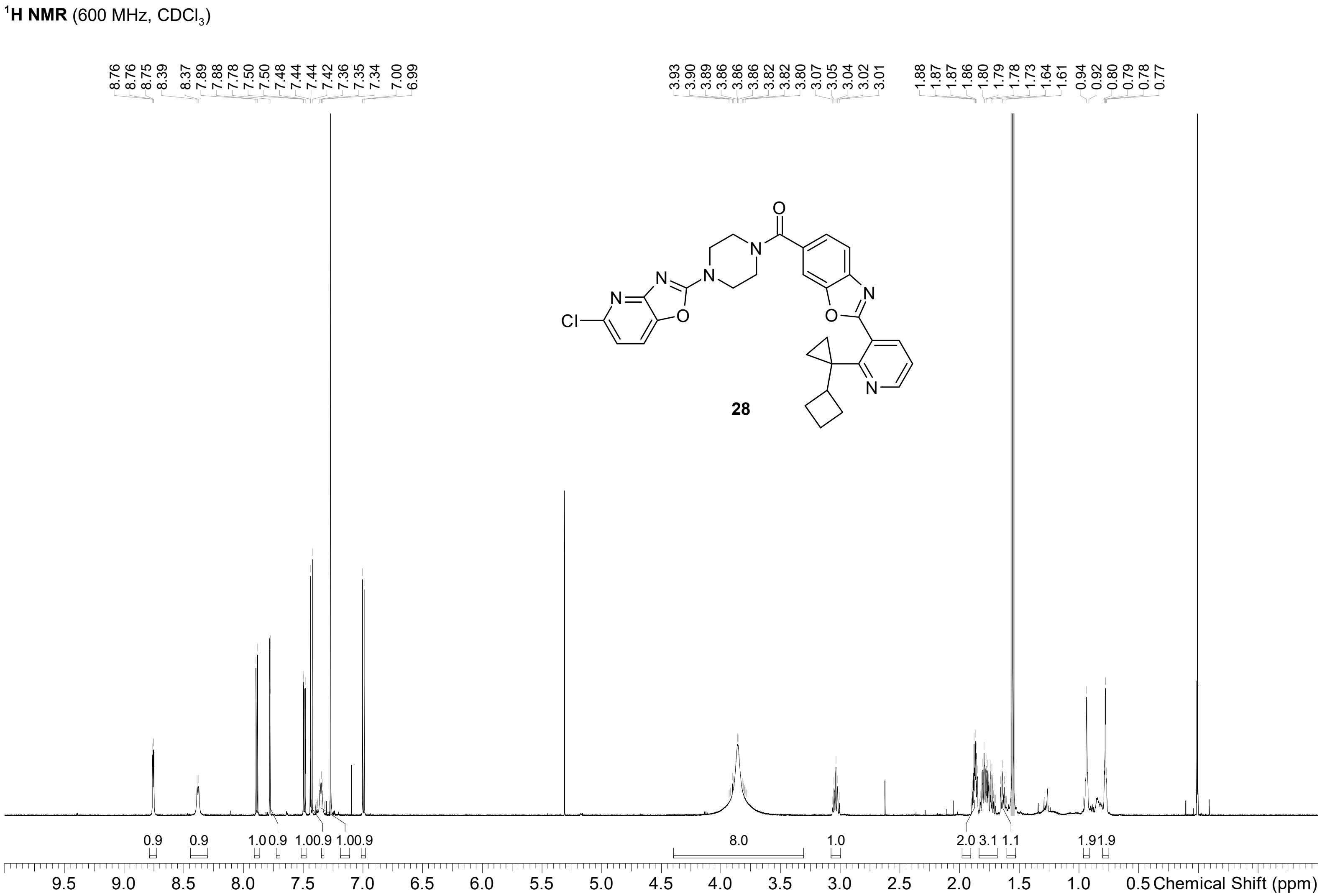

Figure S69: **28**, ^{1}H-NMR spectra.

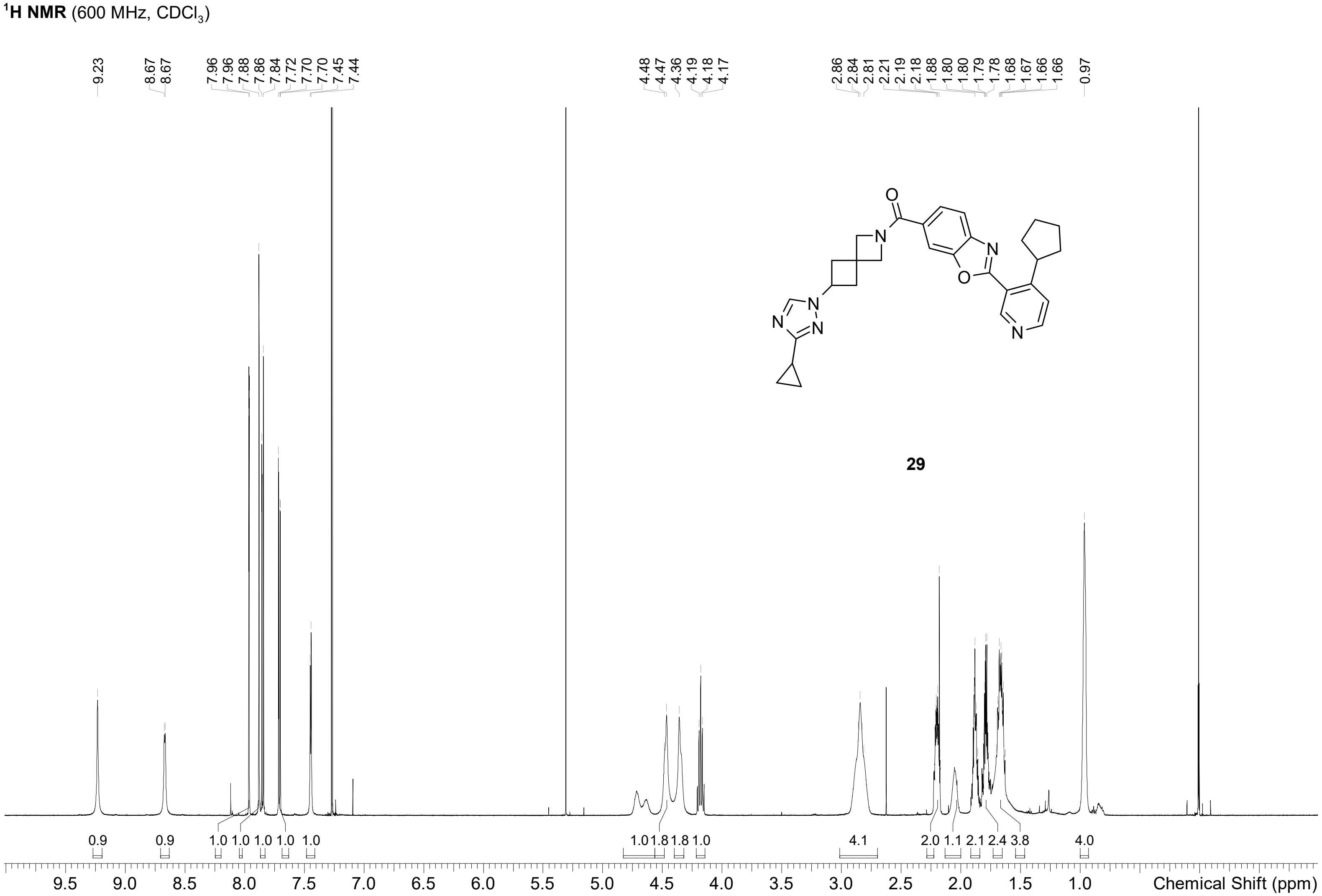

Figure S70: **29**, ^{1}H-NMR spectra.

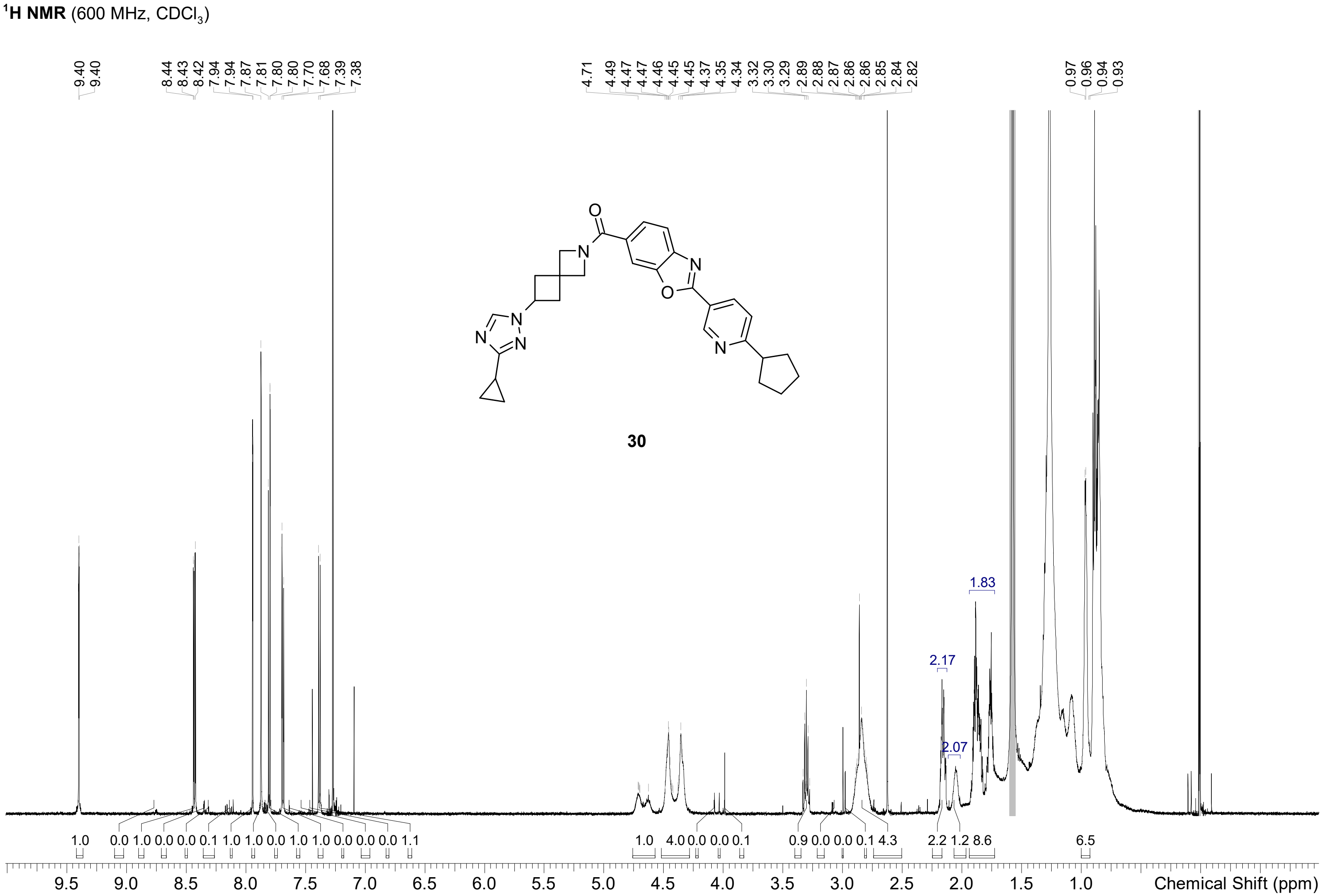

Figure S71: **30**, ^{1}H-NMR spectra.

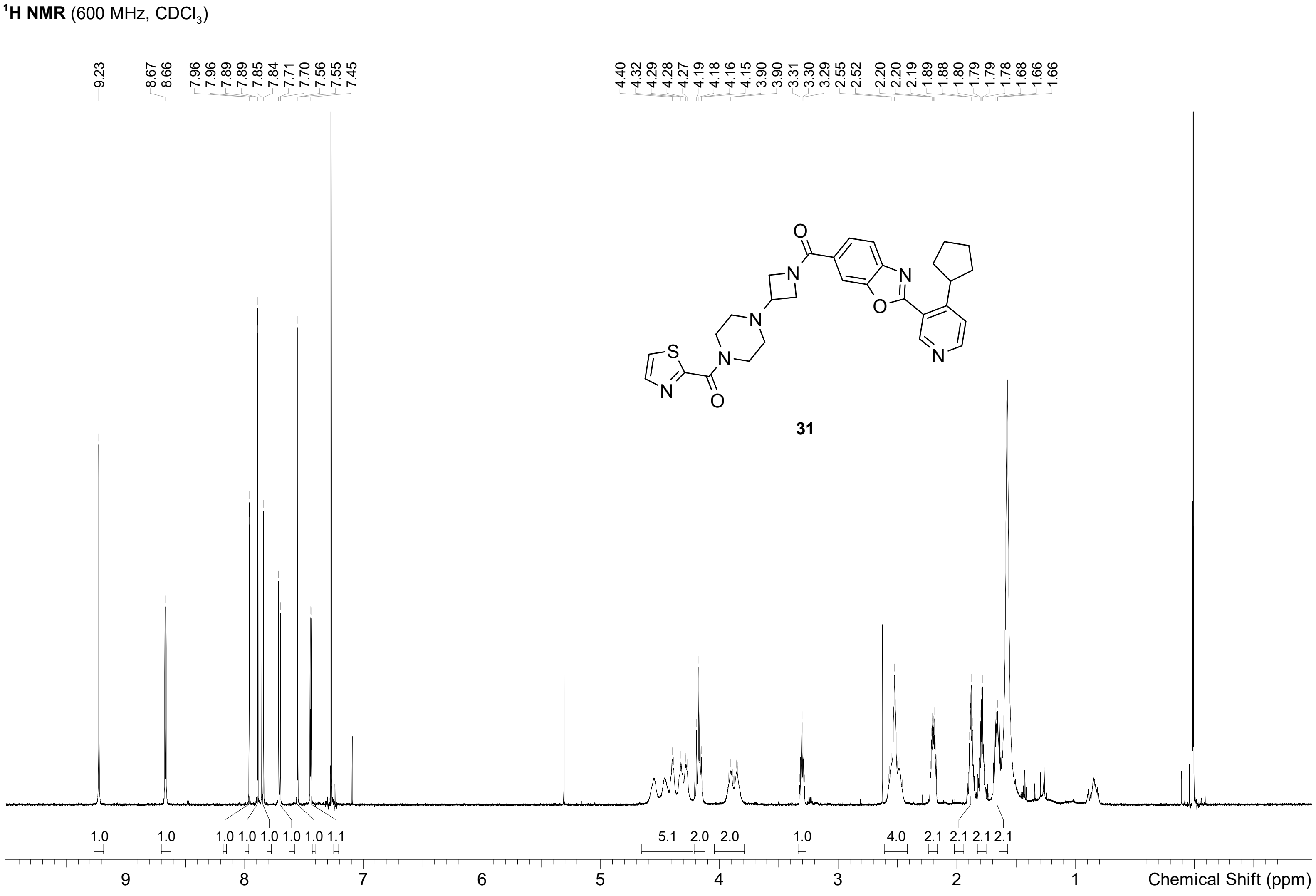

Figure S72: **31**, ^{1}H-NMR spectra.

References

1. Hermann, K. F. *et al.* Kinetics of lipid bilayer permeation of a series of ionisable drugs and their correlation with human transporter-independent intestinal permeability. *Eur. J. Pharm. Sci.* **104,** 150–161 (2017).
2. Fischer, H. *et al.* Calculation of an apical efflux ratio from P-glycoprotein (P-gp) in vitro transport experiments shows an improved correlation with in vivo cerebrospinal fluid measurements in rats: Impact on P-gp screening and compound optimization. *JPET* **376,** 322–329 (2021).
3. Di, L. *et al.* Mechanistic insights from comparing intrinsic clearance values between human liver microsomes and hepatocytes to guide drug design. *Eur. J. Med. Chem.* **57,** 441–448 (2012).
4. Meneses-Lorente, G. *et al.* In vitro and clinical investigations to determine the drug-drug interaction potential of entrectinib, a small molecule inhibitor of neurotrophic tyrosine receptor kinase (NTRK). *IND* **40,** 68–80 (2022).
5. Nippa, D. F. *et al.* Enabling late-stage drug diversification by high-throughput experimentation with geometric deep learning. *Nat. Chem.* **16,** 239–248 (2024).
