# Supplementary material for: Expediting hit-to-lead progression in drug discovery through reaction prediction and multi-dimensional optimization
Source: Nat Commun. 2025 Nov 26;16:11646. doi: 10.1038/s41467-025-66324-4 (PMC12749626; doi:10.1038/s41467-025-66324-4)
Supplement: Supplementary file 2 — Description of Additional Supplementary Files [file 41467_2025_66324_MOESM2_ESM.docx]

**Description of Additional Supplementary Files**

**Supplementary Dataset 1:** Acids used for enumeration
